# Targeting Rap1b signaling cascades with CDNF: Mitigating platelet activation, plasma oxylipins and reperfusion injury in stroke

**DOI:** 10.1016/j.ymthe.2024.09.005

**Published:** 2024-09-10

**Authors:** Jui-Sheng Wu, Helike Lõhelaid, Chih-Chin Shih, Hock-Kean Liew, Vicki Wang, Wei-Fen Hu, Yuan-Hao Chen, Mart Saarma, Mikko Airavaara, Kuan-Yin Tseng

**Affiliations:** 1 Department of Biology and Anatomy, National Defense Medical Center, Taipei 114, Taiwan; 2 Neuroscience Center, HiLIFE, University of Helsinki, 00014 Helsinki, Finland; 3 Faculty of Pharmacy, University of Helsinki, 00014 Helsinki, Finland; 4 Department of Pharmacology, National Defense Medical Center, Taipei 114, Taiwan; 5 Department of Neurological Surgery, Tri-Service General Hospital and National Defense Medical Center, Taipei 114, Taiwan; 6 PhD Program in Pharmacology and Toxicology, Tzu Chi University, 970 Hualien County, Hualien, Taiwan; 7 Neuro-Medical Scientific Center, Hualien Tzu Chi Hospital, Buddhist Tzu Chi Medical Foundation, 970 Hualien County, Hualien, Taiwan; 8 Department of Medical Research, Hualien Tzu Chi Hospital, Buddhist Tzu Chi Medical Foundation, 970 Hualien County, Hualien, Taiwan; 9 Institute of Biotechnology, HiLIFE, University of Helsinki, 00014 Helsinki, Finland

**Keywords:** ischemic stroke, distal middle cerebral artery occlusion, dMCAo, cerebral dopamine neurotrophic factor, CDNF, Rap1b, platelet activation, oxylipin metabolism

## Abstract

Cerebral reperfusion injury in stroke, stemming from interconnected thrombotic and inflammatory signatures, often involves platelet activation, aggregation and its interaction with various immune cells, contributing to microvascular dysfunction. However, the regulatory mechanisms behind this platelet activation and the resulting inflammation are not well understood, complicating the development of effective stroke therapies. Utilizing animal models and platelets from hemorrhagic stroke patients, our research demonstrates that human cerebral dopamine neurotrophic factor (CDNF) acts as an endogenous antagonist, mitigating platelet aggregation and associated neuroinflammation. CDNF moderates mitochondrial membrane potential, reactive oxygen species production, and intracellular calcium in activated platelets by interfering with GTP binding to Rap1b, thereby reducing Rap1b activation and downregulating the Rap1b-MAPK-PLA2 signaling pathway, which decreases release of the pro-inflammatory mediator thromboxane A2. In addition, CDNF reduces the inflammatory response in BV2 microglial cells co-cultured with activated platelets. Consistent with *ex vivo* findings, subcutaneous administration of CDNF in a rat model of ischemic stroke significantly reduces platelet activation, aggregation, lipid mediator production, infarct volume, and neurological deficits. In summary, our study highlights CDNF as a promising therapeutic target for mitigating platelet-induced inflammation and enhancing recovery in stroke. Harnessing the CDNF pathway may offer a novel therapeutic strategy for stroke intervention.

## Graphical abstract

**Figure:**
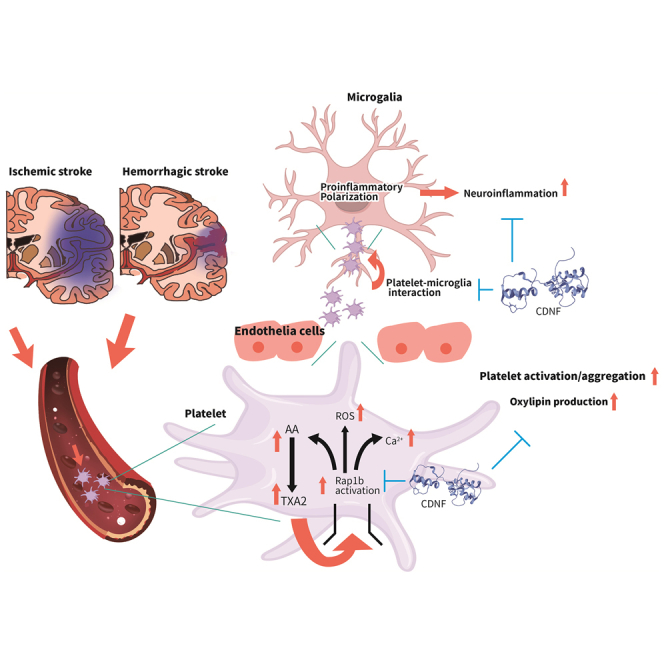

## Teaser

CDNF, a protein naturally residing in the endoplasmic reticulum, attenuates platelet activation and aggregation through specifically mitigating Rap1b activation and reducing TXA_2_ production in platelets when stimulated by arachidonic acid. This action helps attenuate thrombo-inflammation and elevated oxylipin levels after ischemic stroke.

## Introduction

Stroke is a significant global health issue, ranking among the top causes of mortality and adult disability worldwide.^1^ Currently, the primary pharmacological intervention worldwide is a recombinant tissue-type plasminogen activator used to recanalize the thrombosed artery.^2^ In addition, endovascular therapy, extending the therapeutic time window and improving reperfusion rates, has emerged as a significant advancement in stroke treatment.^3^ Despite the prompt restoration of blood vessel patency, many patients suffer from secondary infarct growth, ischemia/reperfusion injury, which paradoxically might even worsen the outcome and should be prevented.^4^

Growing evidence indicates that platelet-mediated thrombo-inflammatory response is a pivotal pathophysiological contributor to the reperfusion brain injury following an ischemia insult.^5^ Platelets, derived from megakaryocytes, are classically regarded as the significant actor of primary hemostasis. However, when platelets adhere to atherosclerotic plaque erosion or rupture, they become activated and aggregated to trigger the formation of blood clots, leading to severe atherothrombotic diseases, such as cerebral ischemia or myocardial infarction.^2^ Beyond their crucial roles in hemostasis and thrombosis, emerging evidence suggests that platelets play a vital role in inflammatory and immune responses.^6^^,^^7^ A close interaction of platelet receptors with other immune cells, such as neutrophils, monocytes, or brain endothelial cells portrays the mechanism of platelet-mediated thrombo-inflammation, which leads to the progression of neuronal damage in ischemic stroke.^8^^,^^9^^,^^10^ This interaction between activated platelets and immune cells induces macrophage pro-inflammatory cytokine secretion.^11^ Furthermore, several studies have demonstrated that platelet-generated lipids influence the regulation of hemostasis, vascular integrity, inflammation, and the development of pathologies such as arterial and deep vein thrombosis, as well as atherosclerosis.^12^^,^^13^ Hence, targeting platelet-mediated thrombosis, neuroinflammation and lipid metabolism could become an effective adjunct therapy to improve outcomes after ischemic stroke.

Cerebral dopamine neurotrophic factor (CDNF) and mesencephalic astrocyte-derived neurotrophic factor (MANF) have garnered attention due to their evolutionary conservation and widespread expression in the central nervous system (CNS), peripheral tissues, and even in innate immune cells.^14^^,^^15^ Both proteins, harboring C-terminal endoplasmic reticulum (ER) retention sequences, exhibit dual subcellular localization, predominantly within the ER, while also showing potential for secretion under ER stress conditions. In addition to ER calcium depletion, increased radical oxidative stress or aberrant protein glycosylation leads to the accumulation of misfolded proteins in the ER and subsequently triggers the unfolded protein response (UPR), and activation of MANF and CDNF genes.^16^^,^^17^ Several studies have demonstrated that MANF and CDNF, which are upregulated in response to ER stress, could alleviate UPR to attenuate ER stress-induced cell death.^18^ Beyond their role in modulating the UPR, a SAP-like (SAF-A/B, acinus, and PIAS) domain of MANF was shown to bind to pro-inflammatory transcription factor NF-κB p65 in immune cells to decrease pro-inflammation cytokine production.^19^ In the rodent brain, CDNF or MANF were shown to directly modulate the activation of innate immune cells to exert their anti-inflammatory effects within the nervous system.^20^^,^^21^^,^^22^^,^^23^

Apart from their role as negative regulators of inflammation, the presence of a SAPLIP domain in MANF and CDNF, which shares structural similarities with granulysin, was proposed to interact with lipids.^16^ MANF has been found to interact with a lipid sulfatide, a specific type of lipid present in the extracellular leaflet of cell membranes, suggesting that MANF may have a role in lipid metabolism involving sulfatide. Importantly, MANF was shown to directly interact with lipid kinase PIP4K2b, enhancing its ability to utilize GTP for phosphorylating PI-5-P to PI-4,5-P_2_, which is a critical lipid species involved in various cellular signaling processes.^24^ However, the lack of evidence demonstrating CDNF’s regulation of PIP4K2b activity and interaction with sulfatide, as observed for MANF, raises an intriguing question regarding whether CDNF binds to different types of kinases than MANF or if it binds to them in stressed condition. While CDNF level was shown upregulated in the infarcted cortex,^25^ a significant reduction in CDNF mRNA levels was observed in peripheral platelets following ischemic stroke in male patients.^26^ In addition, our previous study revealed that heme oxygenase-1 (Hmox1) is significantly downregulated in the hemorrhagic striatum of Cdnf^−/−^ mice, indicating that the absence of CDNF reduces the induction of Hmox1 after hemorrhagic stroke.^22^ However, impaired induction of Hmox1 has been linked to accelerated platelet-dependent arterial thrombosis,^27^^,^^28^ implying that endogenous CDNF may also play a role in circulating platelet activity. These intriguing findings lead us to question whether this corresponding change might be the underlying mechanism that drives platelet aggregation, induces inflammation, and exuberates reperfusion injury after stroke. Meanwhile, CDNF, as an anti-inflammatory protein likely linked to lipid metabolism and platelet activity, might exert a broader systemic effect, and is implicated in platelet-mediated pathological mechanisms following ischemic stroke.

**Figure 1:**
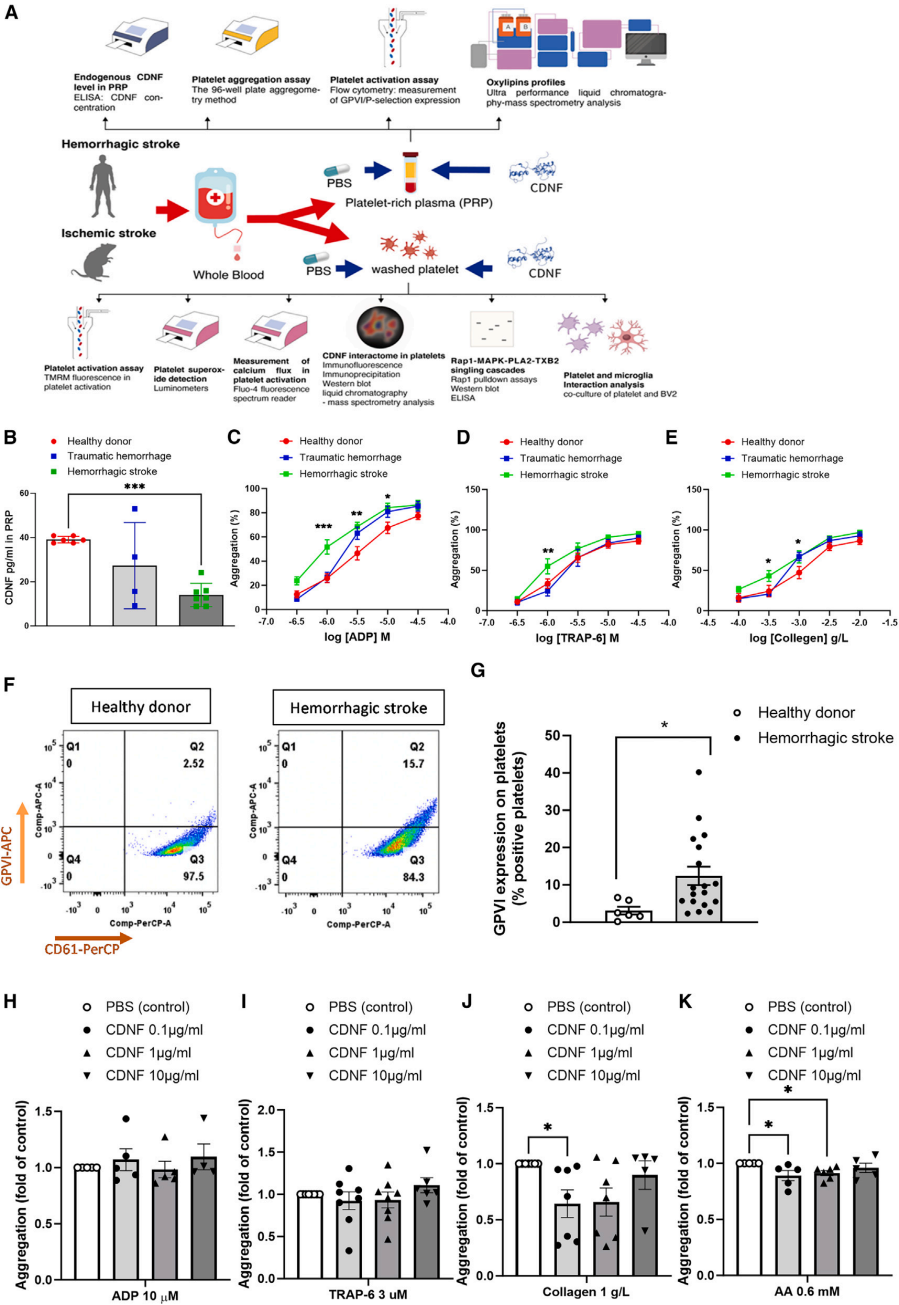
PRP derived from hemorrhagic stroke patients exhibits lower levels of CDNF, but higher aggregation responses compared with healthy controls Extracellularly added CDNF suppressed certain agonist-induced platelet aggregation in PRP. (A) A flow diagram illustrates the experimental design process used in this study. (B) CDNF concentrations in PRP were measured using ELISA in healthy controls, traumatic hemorrhage, and hemorrhagic stroke patients. ∗∗∗*p* < 0.0001 in comparison with the health donor. Data were analyzed by one-way ANOVA followed by Bonferroni corrections. (C–E) Representative the aggregation rates of healthy donor, traumatic hemorrhagic, and stroke patients’ PRP stimulated with various doses of ADP (C), TRAP6 (D), or collagen (E). Data were analyzed one-way ANOVA + post hoc Bonferroni test, ∗*p* < 0.05, ∗∗*p* < 0.01, ∗∗∗*p* < 0.0001 in comparison with the healthy controls. (F and G) Changes in platelet glycoprotein VI (GPVI) expression were observed in PRP obtained from healthy donors or stroke patients by flow cytometry. (G) Quantification of GPVI^+^/CD61^+^ expression in PRP (*n* > 6, each group) were analyzed as two-tailed Student’s t test. ∗*p* < 0.05 in comparison with the healthy donors. (H–K) Aggregation responses of PRP obtained from healthy controls and patients with traumatic brain hemorrhage or hemorrhagic stroke treated with different agonists. Collagen- (J)- and AA- (K) induced stroke patient’s PRP aggregation were significantly suppressed by CDNF treatment in an agonist dose-dependent manner. One-way ANOVA + original FDR method of Benjamini and Hochberg, ∗*p* < 0.05 in comparison with the PBS group. Mean ± SEM is shown.

To address these concerns, we designed a series of experiments to investigate the effects of CDNF on platelet activity, lipid mediator biosynthesis, and neuroinflammatory responses in stroke patients and rats (Figure 1A). Through the analysis of mitochondria membrane potential (TREM), superoxide levels, and calcium flux in washed human platelets, we demonstrated that the CDNF maintains the mitochondria homeostasis, decreases reactive oxygen species (ROS) production, and downregulates intracellular calcium transients during the process of arachidonic acid (AA)-induced platelet activation. Using a combination of immunoprecipitation (IP) and liquid chromatography mass spectrometry (LC-MS), we identified the CDNF interacting with small cytoplasmic GTP binding protein Rap1b in AA-treated platelets. By downregulating Rap1b activity, CDNF suppressed the ERK/cPLA_2_ signaling pathway, which subsequently decreased cPLA_2_ activation and thromboxane B_2_ (TXB_2_) production in AA-treated platelets. In addition, the co-culture of BV2 microglial cells with CDNF-treated platelets from stroke patients exhibited reduced polarization toward a pro-inflammatory phenotype. In rat cortical stroke model, subcutaneous (s.c.) delivery of CDNF was shown to suppress stroke-induced platelet activation, aggregation responses, and subsequent neuroinflammation. Notably, CDNF treatment not only reduced TXB_2_ production in platelets but also normalized the levels of stroke-upregulated COX, 12-lipoxygenase (LOX), and CYP450-derived oxylipins to baseline levels. Given that systemic treatment with CDNF reduced platelet-mediated inflammatory responses after cortical ischemic stroke, it demonstrated the capacity to decrease infarction volume, alleviate neurological deficits, and further mitigate ischemia-induced blood-brain barrier (BBB) disruption. These findings indicate that targeting platelet activation and its immune function with CDNF could be a promising strategy for the therapy in ischemic stroke.

## Results

### Lower CDNF protein expression is accompanied by elevated aggregation responses and increased activation of platelets in the platelet-rich plasma of stroke patients

This study first investigated the association between endogenous CDNF levels and platelet biological function within the 24-h interval following hemorrhagic stroke. Using the ELISA technique, we quantified the levels of endogenous CDNF protein in platelet-rich plasma (PRP) samples collected from healthy individuals and traumatic hemorrhage and hemorrhagic stroke patients. Our analysis revealed a significant decrease in the CDNF protein levels among patients with hemorrhagic stroke compared with healthy donors (Figure 1B). In addition, the human PRP samples were exposed to various platelet activators, such as thrombin receptor-activating peptide 6 (TRAP-6), adenosine diphosphate (ADP), or collagen. PRP aggregation of hemorrhagic stroke patients was significantly increased compared with both healthy donors and patients with traumatic hemorrhage (Figures 1C–1E), suggesting that platelets in the hemorrhagic stroke group exhibit an increased susceptibility to the activators, leading to enhanced aggregation responses. Furthermore, significant upregulation expression of glycoprotein VI (GPVI), a glycoprotein receptor for collagen in platelets, indicative of platelet activation, was observed in PRP obtained from stroke patients compared with healthy individuals (Figures 1F and 1G). These results suggest that CDNF level in PRP may play a crucial role in the process of platelet activation or aggregation.

### CDNF treatment suppresses the platelet aggregation responses induced by collagen or AA in PRP derived from hemorrhagic stroke patients

Since higher aggregation responses along with lower CDNF levels in PRP were found in the stroke group, we sought to investigate whether exogenous CDNF supplementation could potentially regulate PRP aggregation responses. Thus, we treated PRP samples from hemorrhagic stroke patients with various platelet activators (ADP, TRAP-6, collagen, or AA) and exposed them to different doses of CDNF (0.1, 1, or 10 μg/mL). While added CDNF exhibited no discernible effect on the aggregation responses of PRP induced by ADP or TRAP-6 (Figures 1H and 1I), the presence of 0.1 μg/mL of CDNF downregulated collagen-induced aggregation responses of PRP from stroke patients (Figure 1J). Notably, the addition of CDNF at either 0.1 or 1 μg/mL significantly mitigated AA-induced aggregation responses of PRP derived from stroke patients compared with the PBS group (Figure 1K). These findings highlight that the therapeutic potential of CDNF in attenuating PRP aggregation may be attributed to inhibition of collagen-mediated signaling transduction and AA metabolic pathways in platelets.

### CDNF hampers the reduction of mitochondrial transmembrane potential, minimizes ROS production, and decreases cytosolic Ca^2+^ concentration in human washed platelets when exposed to stimulation by platelet activators

**Figure 2:**
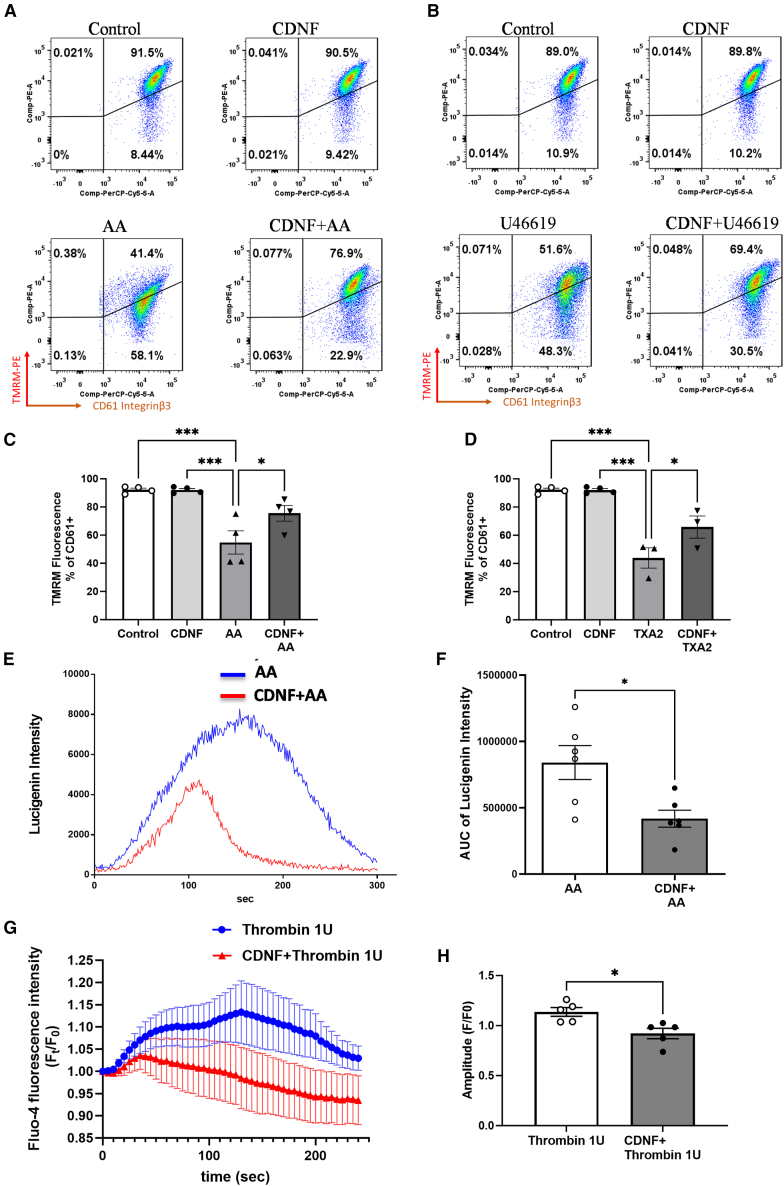
Extracellularly delivered CDNF hinders the decrease in mitochondrial membrane potential (TREM), reduces ROS production, and inhibits Ca^2+^ efflux in arachidonic acid-treated platelets (A–D) Changes in the level of mitochondrial membrane potential (TREM) of platelet stimulated by AA or TXA_2_ analog, U46619. Decreased TREM florescence was shown in AA- or TXA_2_-treated washed platelets. Pre-treatment of CDNF could maintain mitochondria membrane potential in human platelets in the presence of AA (0.6 mM) or TXA_2_ (30 μM). ∗*p* < 0.05, ∗∗*p* < 0.01, ∗∗∗*p* < 0.0001 by one-way ANOVA followed by Bonferroni corrections. Mean ± SEM is shown. Lucigenin (bis-N-methylacridinium nitrate) is the most used chemiluminescent probe for the detection of superoxide ROS in the cells and tissue. Pre-treatment of CDNF could significantly decrease O_2_^–^ production estimated by lucigenin method in human washed platelets in presence of AA. (E and F) Data were analyzed as two-tailed Student’s t test. ∗*p* < 0.05 (*n* = 6). Plate reader Fluo-4-loaded platelets were stimulated in black 96-well plates (FLUOStar Plate Reader; excitation, 485 nm; emission, 520 nm) to determine the concentration of cytoplasmic calcium. (G and H) Shown are (G) mean florescent intensity (Ft/F0) and (H) statistical analysis of platelets with responses reaching the threshold for detection relative to amplitude (F/F0) in response to 1 U/mL thrombin. Data were analyzed as two-tailed Student’s t test. ∗*p* < 0.05 (*n* > 40). When platelets were stimulated with the thrombin, these platelets had high Flur-4 fluorescence. However, pre-administration of CDNF could significantly reduce Fluo-4 florescent amplitude of the thrombin-treated washed platelets.

Given that CDNF treatment was shown to counteract AA-mediated PRP aggregation, we wanted to determine whether CDNF could directly affect certain cellular aspects of the platelet activation response, such as mitochondrial transmembrane potential (ΔΨm) and redox status. Using the cationic dye TMRM in combination with CD61 staining, our findings revealed that the stimulation of human washed platelets with AA or TXA_2_ analog (U46619) results in a marked loss of TMRM staining within 5 min (Figures 2A and 2B). In contrast, when washed platelets were solely stimulated with CDNF, no significant difference in TMRM staining was observed compared with the control group (Figures 2C and 2D). This observation is consistent with previous studies demonstrating that loss of ΔΨm, presented as a decrease in TMRM staining, occurs during activation of human platelets exposed to platelet activators.^29^^,^^30^ In contrast, the administration of CDNF in AA- or U46619-stimulated platelets could significantly rescue the decreased TMRM staining (Figures 2A–2D), implying that CDNF treatment dampens loss of ΔΨm during the process of platelet activation. Since changes in the redox status occur physiologically during platelet activation, detecting ROS levels is the available method to monitor platelet activation and its oxygen-radical generation.^31^ In this study, we utilized chemiluminescent (CL) dyes, Lucigenin (bis-N-methylacridinium nitrate), to detect platelet-released superoxide anion. The administration of CDNF effectively suppressed the AA-induced CL intensity (Figure 2E). The differences in total superoxide anion activities (AUC) during the 5-min period after AA treatment were compared between the AA and CDNF + AA groups (Figure 2F), suggesting that CDNF could significantly suppress AA-induced cellular ROS production during platelet activation. Apart from ROS and mitochondria, emerging evidence has highlighted the significant role of cytosolic calcium (Ca^2+^) concentrations in regulating platelet function, including activation, aggregation, and modulation of cellular signaling pathways.^32^ To investigate the change of cytosolic Ca^2+^ concentrations during platelet activation, washed platelets were loaded with Ca^2+^-sensitive fluorescent dye, Fluo-4, for 1 h, followed by stimulation with 1 U of thrombin.^33^ The intracellular Ca^2+^ transients induced by thrombin were analyzed by measuring the fluorescent intensity relative to the initial baseline, represented as *F/F0.*^34^ The thrombin-induced fluorescent intensity increased approximately by 15% and peaked after approximately 120 s with a total duration of 6 min (Figure 2G). However, pre-treatment of CDNF significantly lowered the Fluo-4 fluorescent intensity of platelets in the presence of thrombin (Figure 2G). The amplitudes, mean values of *F/F0* during the 6-min period after thrombin treatment, exhibited significant differences between the thrombin and CDNF + thrombin groups (Figure 2H), suggesting that extracellularly delivered CDNF could attenuate thrombin-induced calcium responses during platelet activation.

### CDNF, interfering with Rap1b activation, suppresses the AA-induced human washed platelet activation

**Figure 3:**
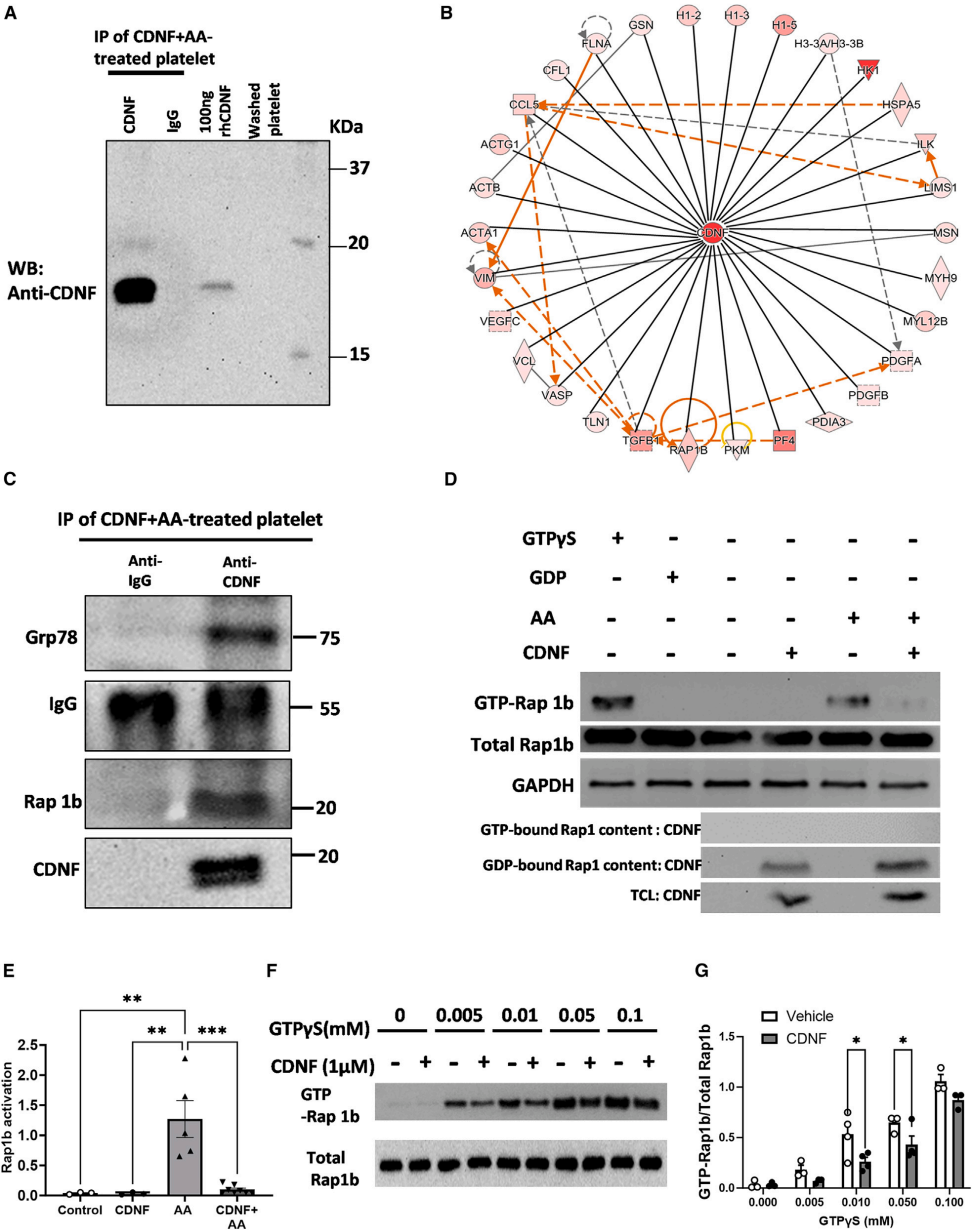
Exogenous CDNF interacts with Rap1b to interfere with GTP-bound Rap1b active form in AA-stimulated human platelets (A) Through immunoprecipitation and western blot analysis, it was shown that adding CDNF antibody to human platelets treated with CDNF exhibited greater efficacy in capturing CDNF compared with IgG. The CDNF served as the positive control in the experiment. (B) The protein interactome networks show CDNF and CDNF-interacted proteins. The representative protein interaction network was generated using IPA software from CDNF-treated human platelets in the presence of AA. (C) Through co-immunoprecipitation (coIP) and western blot analysis, the findings provided evidence of the interaction between CDNF and Rap1b in human platelets treated with AA and supplemented with CDNF. Consistent with the previous study, we also found that CDNF could interact with GRP78 (Bip). (D) The immunoblots show active Rap1b (Rap1-GTP) isolated by the pull-down assay with GST-RalGDS-RBD and the level of total Rap1b present in the lysates used for the Rap1b activation assays. A representative western blot was conducted to visualize Rap1b-GTP levels in human platelets under different conditions: untreated (alone), treated with CDNF, stimulated with AA alone, or treated with a combination of AA and CDNF. The addition of GTPγS and GDP was utilized as a positive control and negative control, respectively for assessment. (E) Quantitation of Rap1b activation as a ratio of Rap1b to GAPDH. Two-way ANOVA + post hoc Bonferroni test, ∗∗*p* < 0.01, ∗∗∗*p* < 0.0001 in comparison with the AA group. As shown in (F) and (G), CDNF could suppress accumulation of GTP-bound Rap1b in the GTPrS treatment with concentrations of 0.01, 0.05 mM (GTPrS 0.01 mM: ∗*p* < 0.05 vs. vehicle and vehicle + CDNF; GTPrS 0.05 mM: ∗*p* < 0.05 vs. vehicle and vehicle + CDNF), but not 0.1 mM, indicating that CDNF treatment could suppress Rap1b activation in washed platelets exposed to GTP in a concentration-dependent manner. TCL, total cell lysates.

To further investigate the regulatory mechanisms underlying CDNF-mediated platelet activation, we set out to characterize the protein-protein interactions of CDNF using a combination of IP assay with anti-human CDNF antibody to capture extracellularly applied CDNF, followed by LC-MS analysis. Human CDNF was immunoprecipitated using 4.8 μg of the recombinant rabbit monoclonal antibody to CDNF from both of AA- and CDNF-treated platelet extracts. We did detect CDNF antibody reactive bands in the CDNF-immunoprecipitated samples, with their size approximately corresponding to CDNF protein, but not in IgG control (Figure 3A). Therefore, having confirmed that the extracellularly added CDNF is biologically active in the AA-treated human platelets, LC-MS analysis was conducted to determine CDNF affinity purification of protein complexes in human AA-treated washed platelets (Table S1). To efficiently filter out the contaminating background proteins, the base2 logarithmized fold change ratios of each protein in CDNF + AA-treated platelets were plotted against their respective log10 (*p* values). Significantly enriched hits from CDNF + AA-treated platelets were shown on the volcano plot (Figure S1A). Furthermore, we observed 30 high-confidence protein-protein interactions in the CDNF interactome in CDNF + AA-treated platelets, excluding CDNF itself (Figure 3B; Table S2). In this interaction network consisting of CDNF interactomes from platelets, we found 10 proteins related to integrin signaling including actin α1 (ACTA1), actin β (ACTB), actin γ1 (ACTG1), gelsolin (GSN), integrin-linked kinase (ILK), myosin regulatory light chain 12B (MYL12B), platelet-derived growth factor subunit B (PDGF-B), Ras-related protein 1b (Rap1b), talin 1 (TLN1), and vasodilator-stimulated phosphoprotein (VASP). Furthermore, we performed Gene Ontology Biological Process (GO BP) term overrepresentation analysis of the CDNF interactomes from CDNF + AA-treated platelets. Statistically significant overrepresented GO BP, Cellular Component, and Molecular Function (GO BP, CC, and MF) terms were organized. The two most enriched GO terms from the CDNF interactome were platelet aggregation and alpha granule lumen (Figure S1B), highlighting the potential involvement of CDNF in regulating platelet activation. Since Rap1b serves as in integral mediator in the process of platelet activation, aggregation, and granule secretion,^35^ we used western blot analysis to further verify the interaction of Rap1b with CDNF (Figure 3C). In addition, GRP78, previously identified as one of the CDNF interactors in the HEK293 and INS1 cell lines,^36^ was shown to interact with extracellularly applied CDNF in platelets exposed to AA (Figure 3C). Finally, to explore that the participation of CDNF in Rap1b activity, we investigated whether extracellularly added CDNF could regulate Rap1b activation in platelets exposed to AA. The levels of active Rap1b were analyzed in the lysates of washed platelets treated with/without CDNF in the presence of AA. The addition of GTPrS in platelets was used as positive control for Rap1b activation, while the supplement of GDP in platelets served as a negative control in the Rap1b protein pull-down assay. Figures 3D and 3E show an increase in Rap1b-GTP levels in AA-treated platelets. In contrast, pre-treatment with CDNF decreased upregulation of Rap1b-GTP levels induced by AA (Figures 3D and 3E), suggesting the negative effect of CDNF on Rap1b activity. Through immunoblotting assays, it was observed that the expression of CDNF is exclusively present in the medium enriched with GDP-bound Rap1b from CDNF-treated platelets (Figures 3D and 3E). Thus, to further investigate the adverse effect of CDNF on Rap1b activation associated with its interference with GTP-Rap1b interaction, platelets treated with/without CDNF were subjected to various concentrations of GTPγS. Figures 3F and 3G show that the inhibitory effect of CDNF on Rap1b is predominantly evident at lower concentration of GTPγS, but this effect diminishes when GTPγS concentration reaches 0.1 mM. Overall, these findings reinforce the notion that extracellularly added CDNF, through attenuating AA-induced Rap1b activation, as evidenced by decreased GTP-Rap1b interaction, mitigates platelet aggregation.

### CDNF inhibits AA-induced cPLA_2_ and ERK phosphorylation, suppressing cPLA_2_ activation and thromboxane A_2_ production in human washed platelets

**Figure 4:**
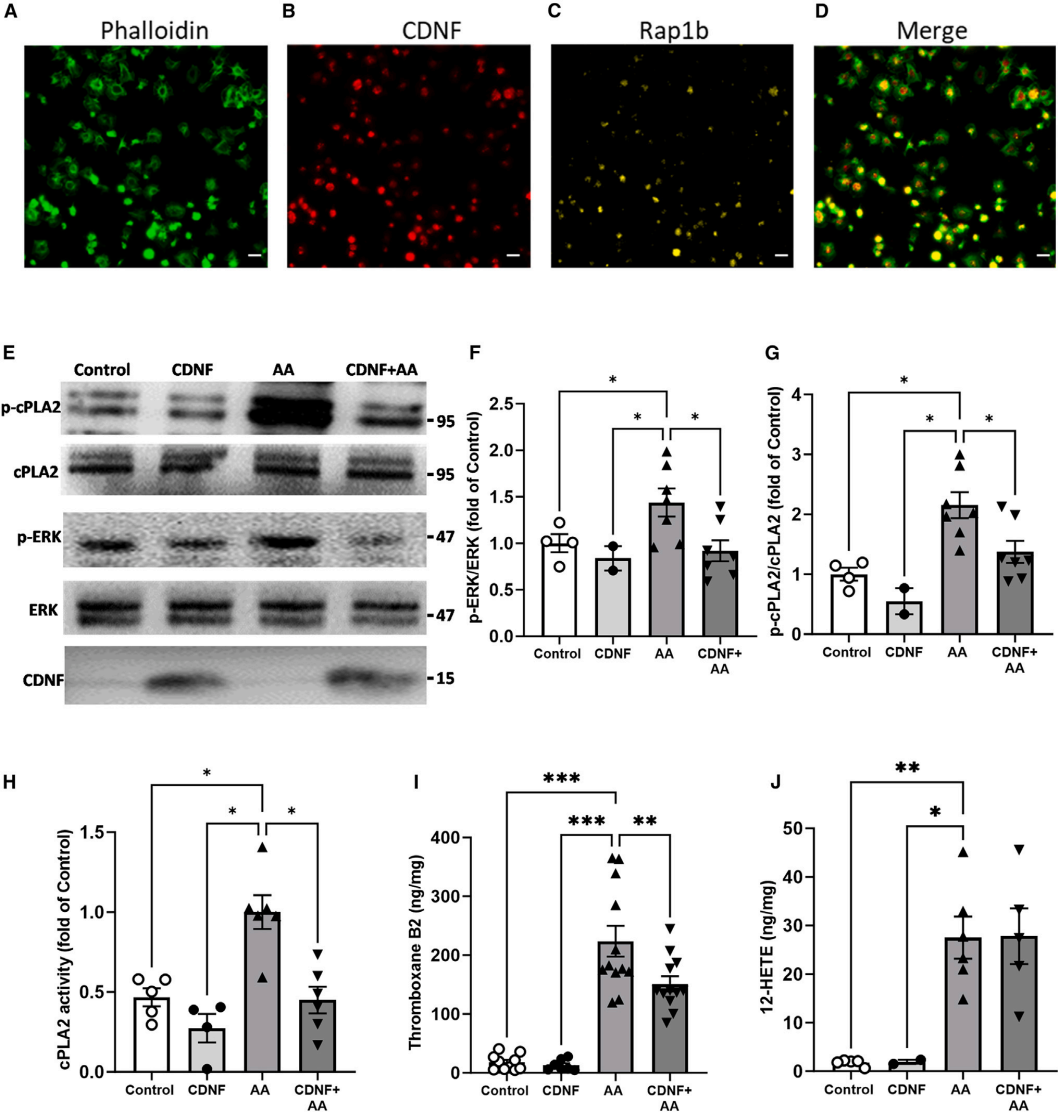
CDNF inhibits the AA-induced phosphorylation of cPLA2 and ERK, resulting in the suppression of cPLA2 activation and thromboxane B2 production, but not 12-HETE synthesis in platelets (A–D) Fluorescently labeled CDNF is detected in phalloidin-stained washed human platelets (A and B). Alexa Fluro 647-labeled-CDNF was co-localized in Rap1b-expressed cells (B and C). A yellowish color in the merge (D) indicates co-localization of CDNF expression in phalloidin and Rap1b-double-positive platelets. Scale bars, 2 μm. The effect of CDNF on AA-induced cPLA2 and ERK phosphorylation in platelets was investigated. Washed human platelets were stimulated with AA in the presence or absence of CDNF. (E–G) The western blot images presented here display the changes in phospho-cPLA_2_, cPLA_2_, phospho-ERK, ERK, and CDNF levels in platelets subjected to treatment with CDNF or PBS, in the presence or absence of AA. The quantification of phosphorylation levels for ERK (F) and cPLA_2_ (G) was represented as a fold change relative to the control group. ∗*p* < 0.05, ∗∗*p* < 0.01 indicates comparison with the AA group with two-way ANOVA and Tukey’s post hoc test. The data represent mean ± SEM. (H) The activity of cPLA_2_ was measured using the cPLA_2_ ELISA assay, revealing that CDNF effectively inhibited the AA-induced increase in cPLA_2_ activity of washed platelets. (I) Thromboxane B_2_ levels were quantified using the thromboxane B_2_ ELISA assay, and the results showed that CDNF effectively reduced the AA-induced increase in platelet thromboxane B_2_ production. (J) The addition of extracellular CDNF did not influence the AA-induced increase in platelet production of 12-HETE. ∗*p* < 0.05, ∗∗*p* < 0.01, ∗∗∗*p* < 0.001 by Tukey’s multiple comparisons test, following one-way ANOVA.

To demonstrate the direct interaction between exogenous CDNF and Rap1b in human washed platelets, we introduced Alexa Fluor 647-labeled CDNF into human washed platelet cultures. Thirty minutes after addition of fluorescently labeled CDNF, the labeled CDNF (Figure 4B) co-localized with Rap1b (Figure 4C) in the phalloidin-expressed platelets (Figures 4A–4D), indicating that extracellularly added CDNF is not only internalized by platelets, but directly bound with Rap1b. Since Rap1b activation participates in ERK and cPLA_2_ signaling pathways,^35^ we evaluated the possibility that extracellularly added CDNF downregulates the transient activation of ERKs and cPLA_2_ in AA-treated platelets. Therefore, we analyzed their phosphorylation status in platelets treated with AA for 1 min in the presence of CDNF. There were no significant differences in the levels of ERK and cPLA_2_ phosphorylation between the platelets treated with PBS (control group) and CDNF, in concordance with the finding that the addition of CDNF does not induce platelet activation (Figure 4E). However, when platelets were stimulated with AA for 1 min, phosphorylation of ERK and cPLA_2_ were induced, which was suppressed by the addition of CDNF, as depicted in Figures 4F and 4G. Next, considering the increased activity of cPLA_2_ following its phosphorylation, we used the ELISA to determine the levels of cPLA_2_ activity in platelets in the presence of AA or CDNF. In line with above data, the analysis of cPLA_2_ activity in platelets revealed that AA participating in the phosphorylation of ERKs and cPLA_2_, leading to activation of cPLA_2_, was suppressed by CDNF treatment (Figure 4H).

The above results indicate that CDNF interferes with the Rap1b-ERK-cPLA_2_ pathway in the process of AA-induced platelet activation. Since this pathway is also responsible for the synthesis of TXA_2_ through the activation of cPLA_2_, we investigated whether extracellularly applied CDNF plays a role in the regulation of TXA_2_ production. ELISA analysis of AA-stimulated platelets revealed that the levels of TXB_2_ (the stable form of TXA_2_) were significantly higher in AA-treated platelets than in the PBS-treated platelets. Indeed, CDNF treatment significantly suppressed the levels of TXB_2_ in the platelets exposed to AA (Figure 4I). While 12-HETE production is also induced through the activation of cPLA_2_ coupled to LOX, another AA-dependent cascade in platelets, it was not blocked by CDNF treatment (Figure 4J), suggesting that the CDNF-mediated suppression of cPLA_2_ activation mitigates the metabolic pathway of AA specifically by inhibiting the coupling of the lipase to COX-1. In summary, our findings indicate that the suppression of Rap1b activation by CDNF effectively inhibits the phosphorylation of cPLA_2_ and ERK, thereby ameliorating the activation of cPLA_2_ and subsequent production of TXA_2_ in AA-treated human washed platelets.

### Inflammatory responses in BV2 microglial cells were suppressed when they were exposed to CDNF-treated platelets derived from hemorrhagic stroke patients

**Figure 5:**
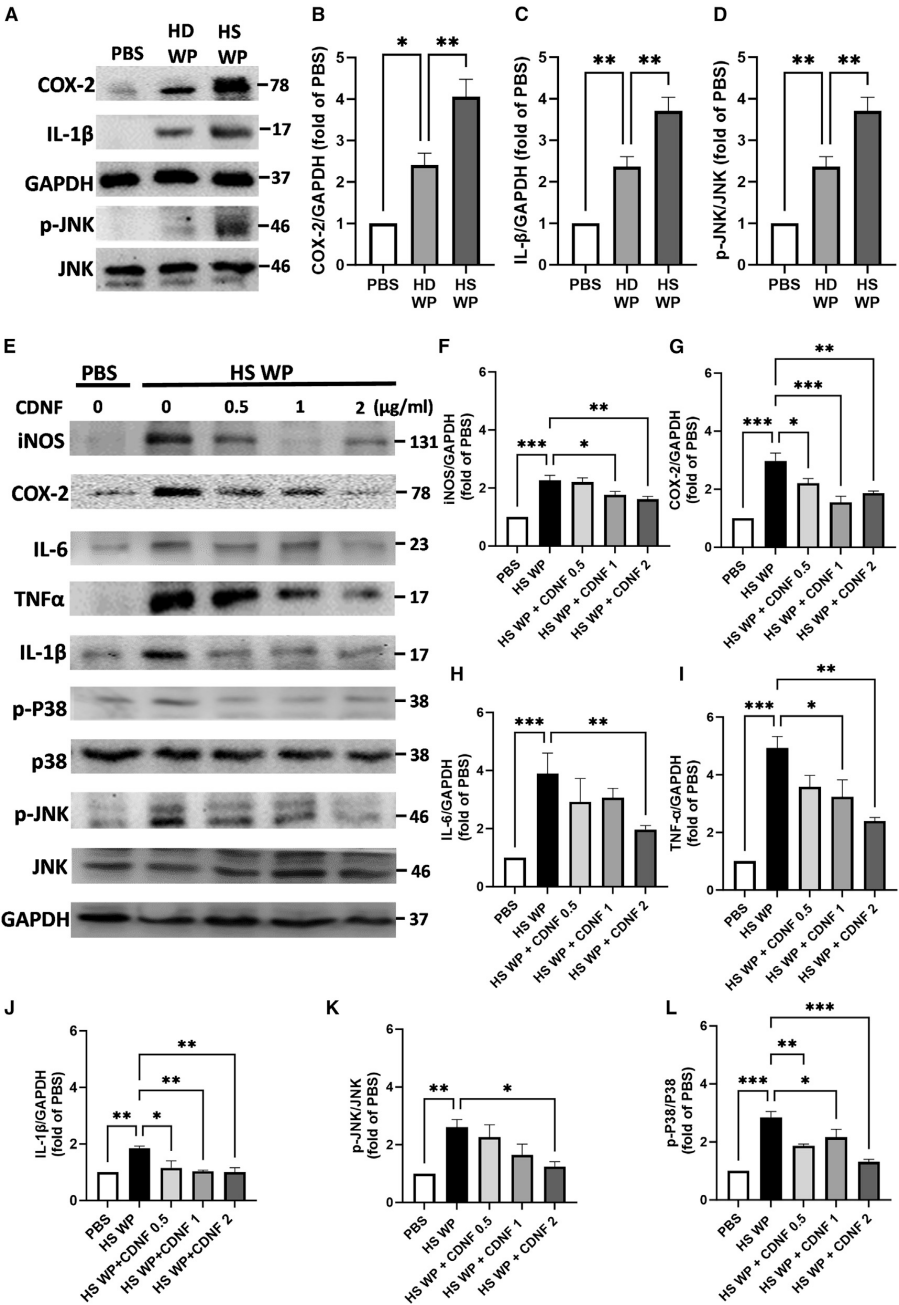
Pretreatment of CDNF attenuated the p-JNK and p-p38 signaling pathway, thereby suppressing the upregulation of iNOS, COX-2, IL-6, TNF-α, and IL-1β in BV2 microglia cells co-cultured with platelets from stroke patients (A–D) BV2, a microglia cell line, was treated with PBS, normal human washed platelets, or washed platelets obtained from patients with hemorrhagic stroke. Western blot assays were employed to determine the expression levels of COX-2 (B), IL-1β (C), and the upstream regulator p-JNK (D), with GAPDH serving as the internal control. The findings revealed that washed platelets from hemorrhagic stroke patients further boost inflammatory responses in BV2 cells, as evidenced by higher levels of COX-2, IL-1b, and p-JNK compared with cells treated with PBS or washed platelets from healthy donors. Data are shown as mean ± SEM (*n* > 3; ∗*p* < 0.05, ∗∗*p* < 0.01 by Tukey’s multiple comparisons test, following one-way ANOVA). (E–L) The addition of washed platelets from hemorrhagic stroke into BV2 microglial cells for 6 h elicited upregulated expressions of iNOS, IL-6, COX-2, IL-1β, TNF-α, p-JNK, and p-P38. When CDNF-treated washed platelets from hemorrhagic stroke patients were co-cultured with BV2 microglial cells, a dose-dependent inhibitory effect was observed on the expression of pro-inflammatory cytokines and their upstream regulators. The quantitative analysis of all the obtained results present as follows (F–L). ∗*p* < 0.05, ∗∗*p* < 0.01 indicates comparison with HS WP group with one-way ANOVA and Tukey’s post hoc test. The data represent mean ± SEM. HD WP, healthy donor washed platelets; HS WP, hemorrhagic stroke washed platelets. CDNF 0.5, 0.5 μg/mL; CDNF 1, 1 μg/mL; CDNF 2, 2 μg/mL.

To delineate the contribution of platelets to neurovascular inflammation following brain injury, BV2 microglial cells were co-cultured with washed platelets for 6 h and then collected to determine the levels of microglia-related pro-inflammatory mediators. Initially, we analyzed the levels of COX-2, IL-1β, and the upstream mediator JNK in BV2 cells cultured alone (PBS group) and in the presence of platelets derived from either healthy donors or hemorrhagic stroke patients. Western blot analysis revealed that there were significantly increased expressions of COX-2 (4 ± 0.34-fold increase), IL-1β (3.7 ± 0.26-fold increase), and phospho-JNK (p-JNK) an upstream regulator of the inflammation signaling pathway (4 ± 0.3-fold increase) in BV2 cells co-cultured with healthy platelets, compared with the cultured alone. In addition, when BV2 cells were co-cultured with platelets derived from hemorrhagic stroke patients, the levels of COX-2, IL-1β, and p-JNK were found to be higher compared with BV2 cells in the presence of healthy platelets (Figures 5A–5D), suggesting that platelets derived from hemorrhagic stroke patients might induce a greater shift of microglia toward a pro-inflammatory phenotype. Next, to investigate whether extracellularly added CDNF could mitigate platelet-induced pro-inflammatory responses in BV2 microglia, CDNF (1 μg/mL) was administered in the co-culture of BV2 microglial cells and platelets derived from stroke patients. Nevertheless, CDNF treatment did not affect the upregulated expression of iNOS or COX-2 in the BV2 cells co-cultured with platelets derived from stroke patients (Figures S2A–S2C). Considering the direct suppressive effect of pre-treating platelets with CDNF on AA-induced platelet activation, the therapeutic protocol was adapted to involve the pre-treatment of platelets from hemorrhagic stroke patients with CDNF at concentrations of 0.5, 1, or 2 μg/mL for 10 min prior to co-culturing with BV2 cells for 6 h. Remarkably, pre-treatment of CDNF significantly attenuated the platelet-induced upregulation of inflammatory mediators, including iNOS, COX-2, IL-6, TNF-α, and IL-1β in the co-culture of BV2 cells in a dose-dependent manner (Figures 5E–5J). The reduction in pro-inflammatory mediator production observed in the co-culture of BV2 cells and platelets treated with CDNF could be attributed to the downregulation of p-JNK and phosphorylated p38 (p-P38) (Figures 5K and 5L). In short, these data reveal that platelets particularly derived from stroke patients could upregulate the production of pro-inflammatory cytokines in BV2 microglial cells. When interacting with BV2 cells, CDNF-treated platelets could hamper pro-inflammatory polarization of these cells. Conversely, when BV2 microglial cells have already interacted with platelets from stroke patients, CDNF treatment fails to reverse the pro-inflammatory polarization of these BV2 microglial cells. These discrepancies might be attributed to CDNF predominantly suppressing the biological activity of platelets derived from hemorrhagic stroke patients.

### CDNF mitigates platelet activation, reduces aggregation responses, and inhibits TXA_2_ synthesis following dMCAo

Based on the above results of CDNF’s influence on platelet activity and prior data demonstrating the capacity of systemic administration of CDNF to modulate inflammatory responses,^22^ we hypothesized that s.c. injection of CDNF may exert regulatory control over the activation of circulating platelets. At first, we wanted to determine that extracellularly added CDNF has similar regulatory effects on the aggregation responses of washed platelets from rats. In line with the findings observed in human data, the addition of CDNF at 0.1 and 1 μg/mL significantly mitigated AA-induced, and collagen-induced aggregation responses of rat WP, respectively (Figures S3A and S3B). Furthermore, flow cytometry analysis revealed that extracellularly added CDNF was able to decease the expression of p-selectin on CD61-positive platelets exposed to AA or collagen stimulation (Figures S4A–S4D), indicating that CDNF could regulate the activation and aggregation responses of rat platelets. Next, considering that the limited bioavailability duration observed with intravenous delivery of CDNF, which was less than 6 h, we collected PRP from rats to assess the platelet aggregation at 6 h post-s.c. administration. While there was no difference in the aggregation response of PRP induced by ADP, P4, or collagen between each group (Figures S5A–S5D), AA-induced aggregation of PRP derived from CDNF-treated rats was significantly decreased than that from both PBS-treated (control group) or MANF-treated rats (Figure S5B).

**Figure 6:**
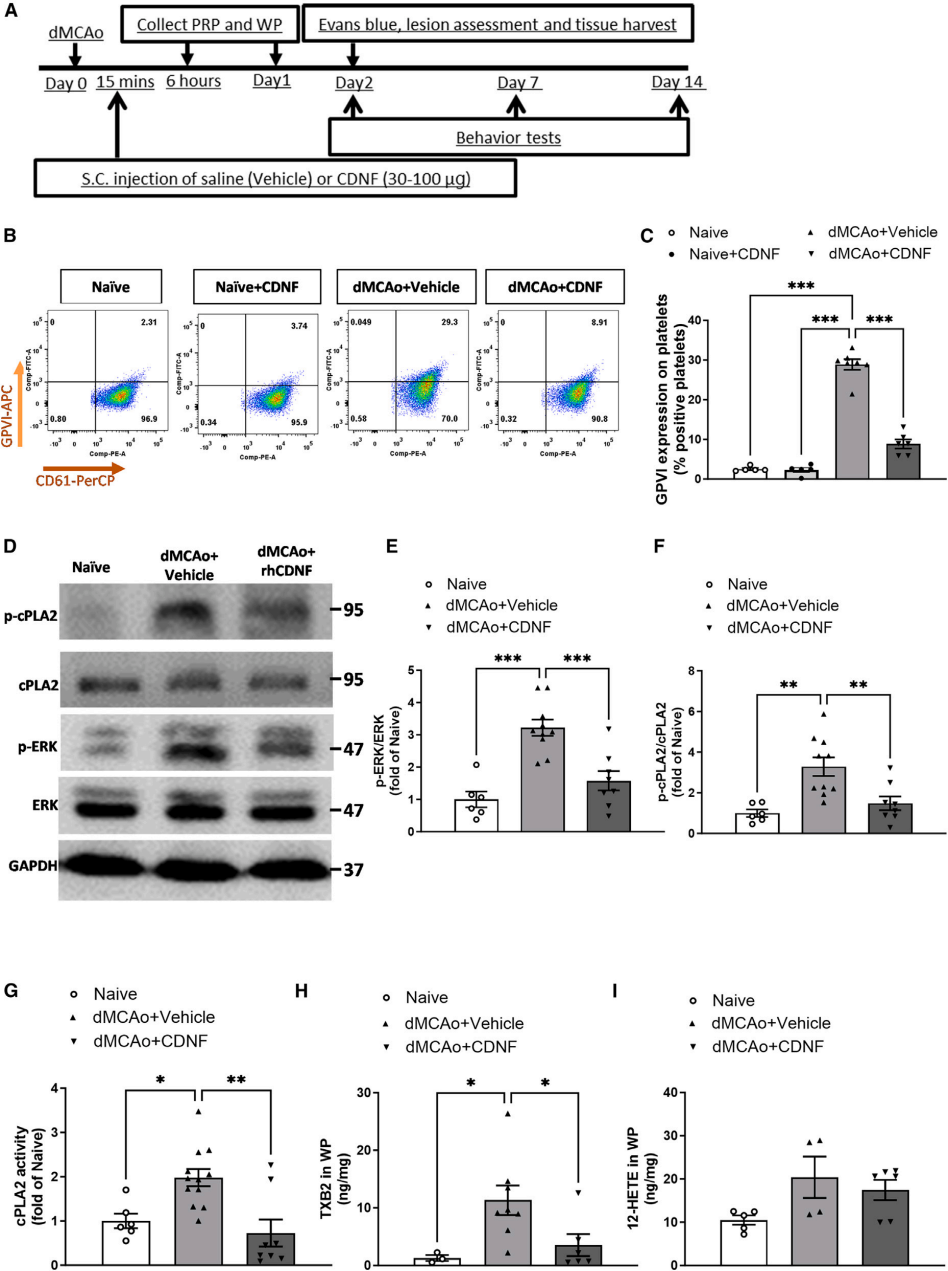
Post-stroke systemic administration of CDNF reduced GPVI expression in PRP and downregulated ERK-cPLA_2_-TXA_2_ signaling transduction in circulating platelets (A) Timetable of experiment. The rats underwent dMCAo surgery, and they were divided into two groups randomly. Fifteen minutes after reperfusion, the animals received an s.c. injection of either CDNF or PBS (vehicle). PRP or washed platelet samples were collected at 6 h and 1 day post-surgery. Behavioral functions were evaluated on days 2, 7, and 14 post-stroke. Rats were sacrificed for analysis at different time points. (B and C) By using flow cytometry, it was observed that CDNF treatment suppressed the elevated expression of GPVI^+^/CD61^+^ in PRP at 6 h after dMCAo. (C) Quantitation of GPVI^+^/CD61^+^ expression in PRP derived from naive, CDNF alone, dMCAo + vehicle and dMCAo + CDNF groups. *n* = 5–6 per group. ∗∗∗*p* < 0.001 by Tukey’s multiple comparisons test, following one-way ANOVA. (D–F) Western blot analysis revealed that systemic administration of CDNF suppressed the upregulated expressions of p-ERK (E) and p-cPLA_2_ (F) in washed platelets at 6 h post-stroke. cPLA_2_ or ERK was used as an internal control for normalization. The statistical analysis of the results is presented as a fold change relative to the naive group. ∗∗*p* < 0.01, ∗∗∗*p* < 0.001 indicates comparison with the dMCAo + vehicle group by Dunnett’s multiple comparisons test, following one-way ANOVA. (G–I) The activity of p-cPLA_2_ as well as production of TXB_2_ and 12-HETE in washed platelets at 6 h after dMCAo were measured using ELISA. The findings revealed that CDNF treatment suppressed stroke-upregulated cPLA_2_ activity (G) and the production of TXB_2_ by platelets (H), while it had no effect on the synthesis of platelet 12-HETE (I). ∗*p* < 0.05, ∗∗*p* < 0.001, Holm-Šidák multiple comparisons test, following one-way ANOVA. The mean ± SEM of three independent experiments is shown.

Given that ischemia/reperfusion injury following ischemic stroke can lead to platelet activation, our objective was to explore whether systemic administration of CDNF has the potential to modulate the aggregation responses or activation of circulating platelets after distal middle cerebral artery occlusion (dMCAo)-induced ischemic stroke. As a proof-of-principle, rats underwent a single s.c. injection of either CDNF at different doses or 500 μL saline (vehicle) 15 min after the dMCAo reperfusion (Figure 6A). Then, PRP aggregation was analyzed at 6 and 24 h post-stroke, respectively. Consistent with the aforementioned findings, s.c. administration of CDNF exhibited a significant reduction in AA-induced PRP aggregation in rats subjected to ischemic stroke (Figures S6C and S6G). However, this negative effect on the agonist-induced PRP aggregation was not observed in the presence of ADP, P4, or collagen (Figures S6B, S6D–S6F, S6H, and S6I). In accordance with the aggregation response findings, a decrease in AA-induced, but not collagen- or P4-induced upregulation of P-selectin expression was observed in PRP obtained from rats that had experienced ischemic stroke and were treated with CDNF (Figures S7A and S7B). However, this effect was not observed in the case of collagen- or P4-induced upregulation of P-selectin expression in the same PRP samples (Figures S7C–S7F). Furthermore, it was observed that the level of the GPVI receptor, a critical mediator of platelet activation, exhibited an upregulation at 6 h after dMCAo when compared with the naive or the naive + CDNF group. The s.c. injection of CDNF was found to significantly suppress the elevated expression of the GPVI receptor in PRP at 6 h following dMCAo (Figures 6B and 6C), suggesting that post-treatment with CDNF has the potential to inhibit circulating platelet activation, as indicated by the suppression of GPVI receptor expression, following ischemic stroke. The observed inhibitory effect on platelet activation following ischemic stroke appears to be associated with a reduction in the phosphorylation levels of ERK and cPLA_2_, along with the downregulation of cPLA_2_ activity in platelets (Figures 6D–6G) at 6 h post-stroke. This suggests that CDNF’s negative impact on platelet activation following ischemia/reperfusion injury may be linked to the Rap1b-ERK-cPLA_2_ pathway, as previously observed in *in vitr*o studies. Given that cPLA_2_ activation is a pivotal factor in AA metabolism within platelets, it is worth noting that CDNF treatment effectively suppressed the production of TXB_2_ (the stable form of TXA_2_) in rat platelets at 6 h following dMCAo (Figure 6H). However, CDNF did not show a similar effect on the production of 12-HETE in platelets at the same time point (Figure 6I). Overall, these findings suggest that CDNF has the potential to regulate the activated phenotype of platelets, thereby mitigating the aggregation responses of PRP in the context of ischemic stroke.

### Plasma oxylipin levels increase after dMCAo and decrease on systemic administration of CDNF

**Figure 7:**
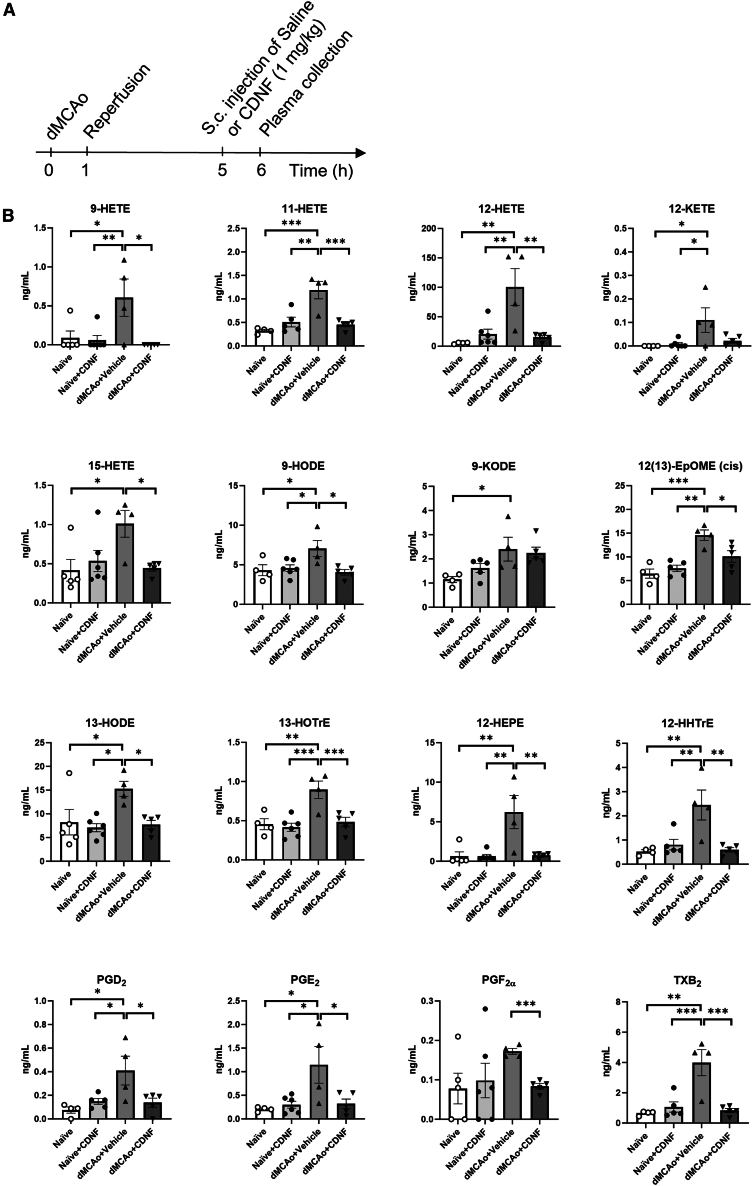
The levels of oxylipins in plasma increase 5 h after ischemic stroke in a rat model and s.c. administration of CDNF reduces the levels down to the baseline (A) Timetable of the experiment. The rats were divided into four groups randomly (*n* = 4–6 animals/group). Two control groups without stroke received an s.c. injection of either CDNF or saline (vehicle). Two stroke groups underwent dMCAo surgery with 60 min occlusion and 4 h after reperfusion the animals received an s.c. injection of either CDNF or saline (vehicle). Rat plasma was collected 1 h after injection. (B) Significantly increased oxylipins after dMCAo surgery and decreased after CDNF treatment: 9-HETE, (±)9-hydroxy-5*Z*,7*E*,11*Z*,14*Z*-eicosatetraenoic acid; 9-HODE, (±)9-hydroxy-10*E*,12*Z*-octadecadienoic acid; 9-KODE, 9-oxo-10*E*,12*Z*-octadecadienoic acid; 11-HETE, 11-hydroxy-5*Z*,8*Z*,11*E*,14*Z*-eicosatetraenoic acid; 12-HEPE, (±)12-hydroxy-5*Z*,8*Z*,10*E*,14*Z*,17*Z*-eicosapentaenoic acid; 12-HETE, (±)12-hydroxy-5*Z*,8*Z*,10*E*,14*Z*-eicosatetraenoic acid; 12-HHTrE, 12*S*-hydroxy-5*Z*,8*E*,10*E*-heptadecatrienoic acid; 12-KETE, 12-oxo-5*Z*,8*Z*,10*E*,14*Z*-eicosatetraenoic acid; 12(13)-EpOME(*cis*), (±)12,13-epoxy-9*Z*-octadecenoic acid; 13-HODE, (±)13-hydroxy-9*Z*,11*E*-octadecadienoic acid; 13-HOTrE, 13*S*-hydroxy-9*Z*,11*E*,15*Z*-octadecatrienoic acid; 15-HETE, (±)15-hydroxy-5*Z*,8*Z*,11*Z*,13*E*-eicosatetraenoic acid; PGD_2_, 9α,15*S*-dihydroxy-11-oxo-prosta-5*Z*,13*E*-dien-1-oic acid; PGE_2_, 9-oxo-11α,15*S*-dihydroxy-prosta-5*Z*,13*E*-dien-1-oic acid; PGF_2α_, 9α,11α,15*S*-trihydroxy-prosta-5*Z*,13*E*-dien-1-oic acid; TXB_2_, 9α,11,15*S*-trihydroxythromba-5*Z*,13*E*-dien-1-oic acid. ∗*p* < 0.05, ∗∗*p* < 0.01, ∗∗∗*p* < 0.001 by Tukey’s multiple comparisons test, following one-way ANOVA. Mean ± SEM is shown.

In platelets, CDNF inhibited cPLA_2_ activity and TXB_2_ synthesis but not 12-HETE generated by COX and LOX pathways, respectively. To clarify the systemic effect of CDNF on lipid mediator biosynthesis we investigated the presence of oxygenated lipid mediators, oxylipins, derived from different PUFAs in rat plasma after dMCAo (Figure 7A). Multiple oxylipins formed from LA (ω:6), AA (ω:6), a-LA (ω:3), and EPA (ω:6) were monitored (Table S3). Indeed, the upregulation of the COX pathway after dMCAo was confirmed by significant increase of AA-derived PGD_2_, PGE_2_, TBX_2_, and 12-HHTrE levels while CDNF reduced the amount of PGD_2_, PGE_2_, PGF_2α_, TBX_2_, and 12-HHTrE detected in plasma (Figure 7B). In parallel, the LOX and CYP450 pathways were also activated after ischemic stroke based on the significant increase of 9-HODE, 9-KODE, 13-HODE, 12-HETE, 12-KETE, 15-HETE, 13-HOTrE, 12-HEPE, and 9-HETE, 11-HETE, 12(13)-EpOME, respectively (Figures 7B and S8). The administration of CDNF reduced their levels down to the baseline. In summary, oxylipin profiling suggests that systemic administration of CDNF after ischemic stroke blocks the production of oxylipins derived by all biosynthesis pathways including COX, LOX, and CYP450s matching with reduced cPLA_2_ activity.

### Systemic administration of CDNF reduces infarction volume, improves neurobehavioral functions, and modulates neuroinflammatory responses after ischemia/reperfusion injury

**Figure 8:**
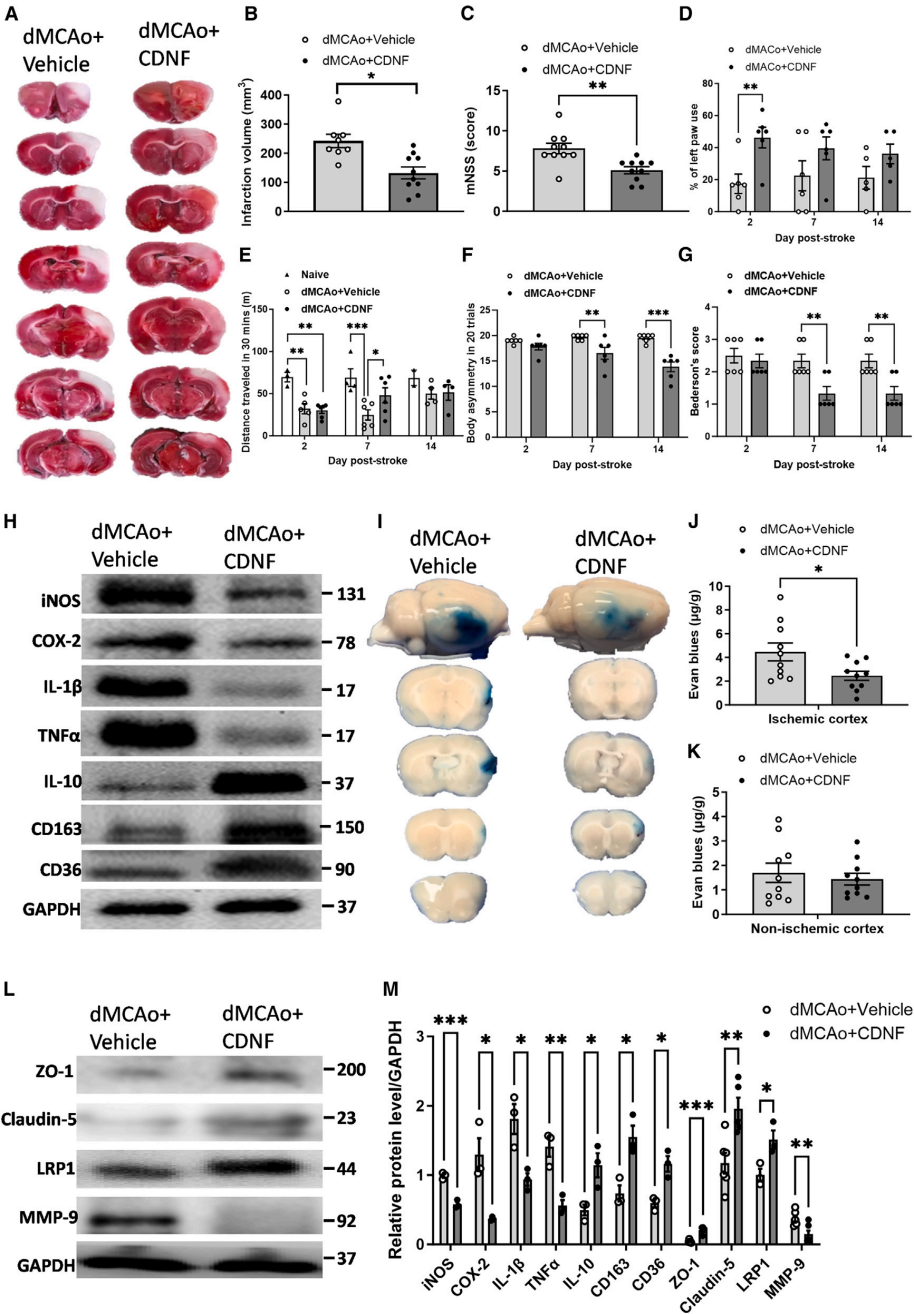
Systemic administration of CDNF decreased infarction volume, accelerated neurobehavioral recovery, attenuated neuroinflammation, and preserved BBB integrity and junction protein expressions in the lesioned cortex after dMCAo (A) Representative images of brain sections stained with TTC showing infarction area. (B) Quantification of infarction volume using TTC staining after 48 h of reperfusion. *n* = 8–10 per group; ∗*p* < 0.05 vs. dMCAo + vehicle; Student’s t test was used for the analysis of statistical significance. (C) Modified neurological severity scores (mNSS) were examined at 2 days post-dMCAo *n* = 8–10 per group. ∗∗*p* < 0.01 vs. dMCAo + vehicle; Student’s t test was used for the analysis of statistical significance. (D) Forepaw use bias of the rats was assessed in the cylinder test at 2, 7, and 14 days after dMCAo. *n* = 5–6 per group. ∗∗*p* < 0.01 by Fisher’s LSD test, following two-way ANOVA (effect of treatment: F(1,28) = 11.77, *p* < 0.0019). (E) Horizontal distance traveled for 30 min on days 2, 7, and 14 post-dMCAo. ∗*p* < 0.05 by original FDR method of Benjamini and Hochberg test, following two-way ANOVA (effect of treatment: F(2,31) = 11.97, *p* < 0.0001). (F and G) Body asymmetry test and Bederson’s score were analyzed on days 2, 7, and 14 after dMCAo, ∗∗*p* < 0.01, ∗∗∗*p* < 0.001 indicate comparison with vehicle with Bonferroni’s post hoc test following two-way ANOVA. (H) Western blot bands of iNOS, COX-2, IL-1β, TNF-α, IL-10, CD163, CD36, and GAPDH at 2 days after dMCAo. (I) Representative brain coronal sections (2 mm thickness) show Evans blue extravasation on day 2 post-dMCAo. (J and K) Comparison of dye concentrations in the ipsilateral (J) and contralateral (K) cortex between dMCAo + vehicle and dMCAo + CDNF groups. Dye concentration is presented as μg/g of tissue weight and calculated from a standard curve obtained from known amounts of dye. ∗*p* < 0.05, paired t test. Mean ± SEM is shown. (L) Western blotting showed the levels of tight junction proteins ZO-1, claudin-5, LRP-1, and 92 kDa type IV collagenase, MMP-9, within the peri-infarct cortical area. (M) At 2 days post-stroke, the decreased levels of iNOS, COX-2, IL-1β, TNF-α, and MMP-9, but increased levels of IL-10, CD163, CD36, ZO-1, Claudin-5, and LRP-1 were detected in the ischemic cortex of CDNF treatment group. ∗*p* < 0.05, ∗∗*p* < 0.01, ∗∗∗*p* < 0.001 by multiple unpaired t test. Mean ± SEM is shown.

Based on the compelling *in vitro* and *in vivo* findings, the next logical step was to explore whether the attenuation of ischemia-induced platelet activation and oxylipin biosynthesis by CDNF could potentially mitigate ischemic brain damage following dMCAo. Following the previously mentioned experimental protocol (Figure 6A), we selected three dosages of CDNF and determined that s.c. injection of CDNF at a dosage of 50 μg/500 μL was effective in reducing the infarction volume, as indicated by TTC staining, at the 2-day mark following dMCAo (Figures S9A and S9B). While systemic administration of CDNF does not affect physiological parameters in healthy rats (Figure S10A), it leads to a reduction in the number of lymphocytes at day 1 after dMCAo compared with the vehicle group (Figure S10B). Neurological function was assessed using the modified neurological severity score (mNSS) at day 2 after dMCAo. Compared with the vehicle group, rats receiving s.c. injection of CDNF exhibited a significantly accelerated recovery (Figure 8C). Furthermore, rats treated with CDNF showed less severe injury-induced behavioral deficits in the cylinder test, specifically in the left forepaw use at day 2 post-dMCAo (Figure 8D). To examine the potential effects of CDNF on the locomotor activity impaired by ischemia/reperfusion injury, open-field tests were conducted. Rats that experienced ischemia/reperfusion injury displayed a notable decrease in both the distance traveled at both the 2- and 7-day time points following dMCAo. However, post-treatment with CDNF promoted the locomotor activity of the experimental rats at the 7-day mark following dMCAo (Figure 8E). In addition, on days 7 and 14 post-stroke CDNF post-treatment decreased both the body asymmetry and Bederson’s score compared with the vehicle-treated group (Figures 8F and 8G), suggesting that CDNF could accelerate neurofunctional recovery after ischemic stroke.

Since ischemia-induced neuroinflammation has been shown to increase the magnitude of cerebral damage and neurological deficits, we wanted to determine the levels of pro-inflammatory mediators and anti-inflammatory cytokine in the lesioned cortex. Quantitative immunoblotting analysis revealed that, on day 2 following dMCAo, the CDNF-treated groups exhibited decreased expression of iNOS, COX-2, IL-1β, and TNF-α, while displaying increased levels of IL-10 within the infarcted cortex compared with the saline-treated group (Figures 8H and 8M). Notably, it resulted in an upregulation of scavenger receptors, particularly CD163 and CD36, primarily within microglia/macrophages located in the infarcted cortex (Figures 8H and 8M), suggesting that systemic delivery of CDNF has the potential to enhance the clearance of necrotic debris and tissue repair following ischemia/reperfusion injury. Thus, this collective effect might contribute to the reduction of neuroinflammation and secondary brain injury in the context of ischemic stroke.

### Systemic administration of CDNF attenuates ischemia-related BBB disruption after dMCAo

Since platelet-mediated thrombo-inflammation plays a crucial role in the breakdown of the BBB upon stroke, the final aim was to explore whether s.c. injection of CDNF, which mitigates circulating platelet activation and aggregation responses, has the potential to preserve the integrity of BBB in rats following dMCAo. We examined the cerebral microvasculature permeability in the Evans blue extravasation assay at 48 h of reperfusion. Tissue leakage of Evans blue exhibited a distinct, diffuse blue discoloration of varying intensities related to ischemia, as depicted in Figure 8I. In the CDNF-treated group, there was a reduced Evans blue extravasation in ischemic hemispheres (2.45 ± 1.22 μg/g tissue) compared with the vehicle group (4.47 ± 2.40 μg/g tissue), as illustrated in Figure 8J. However, there was no notable difference in dye concentration observed in the contralateral cortex between the two groups, as shown in Figure 8K. These findings suggest that post-treatment with CDNF specifically mitigated ischemia-induced BBB leakage, aligning with the observed decrease in MMP-9 levels in the CDNF-treated group (Figures 8L and 8M) when compared with vehicle-treated group. Moreover, a decreased in the tight junction proteins Claudin-5, LRP-1, and ZO-1 was rescued in infarcted cortex by CDNF treatment (Figures 8L and 8M). These results highlight the systemic administration with CDNF, through mitigating thrombo-inflammatory responses in lesioned cortex, could further ameliorate BBB impairment after dMCAo-induced brain injuries.

## Discussion

This study is the first to report that patients with acute hemorrhagic stroke have lower CDNF levels in PRP compared with age-matched healthy controls. This finding aligns with a previous study that demonstrated decreased CDNF gene expression in the platelets of patients with neurodegenerative diseases.^26^^,^^37^ However, the relationship between CDNF expression in platelets and the underlying pathophysiology of nervous system disorders remains unclear and requires further investigation. Stroke, including ischemic and hemorrhagic type, is a leading cause of morbidity and mortality in the realm of neurological disorders. Despite their distinct underlying causes, both types of stroke result in a decrease in blood supply to the brain and subsequent neuroinflammation.^2^ While prompt recanalization through thrombolysis or mechanical thrombectomy offers benefits to selected patients, a considerable number of patients still encounter secondary infarct growth or hemorrhagic transformation after vessel recanalization, which leads to the concept of reperfusion injury.^38^ Recently, it has become clear that ischemia/reperfusion involving thrombotic and inflammatory pathways leads to secondary brain injuries following ischemic or hemorrhagic stroke. One of the major contributors to the thrombotic and inflammatory responses after stroke is pathologic platelet activation.^7^ In cases of vascular injury, platelets adhere to exposed von Willebrand factor by binding their platelet glycoprotein Ibα receptor.^3^ Upon platelet adhesion, binding of collagen to GPVI induces platelet activation by initiating a cascade of tyrosine phosphorylation events.^39^ This sequence results in swift alterations in platelet shape, mobilization of Ca^2+^, and the inside-out activation of integrins, which induces platelet aggregation and the granule release.^40^ Therefore, the higher expression of GPVI has functioned as an indicator of an increased presence of activated circulating platelets in patients with ischemic or hemorrhagic stroke.^41^ Moreover, sustained GPVI activation was demonstrated as an inducer to produce procoagulant microparticles, pro-inflammatory mediators, and inorganic polyphosphate, an activator of coagulation factor XII.^10^ In our study, PRP obtained from stroke patients exhibited lower CDNF levels, while concurrently displaying an increased expression of GPVI and higher aggregation in response to collagen. These findings led us to hypothesize whether CDNF might have a negative effect on platelet activation in response to specific stimuli. Indeed, externally added CDNF was shown to effectively mitigate collagen- or AA-induced aggregation in PRP derived from stroke patients. It has been demonstrated that collagen and AA signaling pathways lead to enhanced ROS generation via NADPH oxidase (NOX) during platelet activation.^42^ Specifically, collagen binding to GPVI triggers the interaction of tumor necrosis factor receptor-associated factor 4 with NOX, resulting in increased ROS production.^43^^,^^44^^,^^45^ During the conversion of AA to TXA_2_ in activated platelets, cyclooxygenase-1 catalyzes the formation of the intermediate prostaglandin H_2_, which generates ROS as a by-product.^46^ Furthermore, TXA_2_ has been implicated in further stimulating ROS production in platelets.^47^^,^^48^ These findings indicate that CDNF treatment suppresses platelet aggregation responses induced by collagen or AA primarily through the reduction of ROS generation. Also, these findings reveal a novel role of CDNF in suppressing the platelet activation process, and the underlying mechanism may be attributed to its involvement in the AA metabolic pathway. During platelet activation, COX-1 transforms AA to TXA_2_, which interacts with TP receptor and functions to amplify platelet activation leading to enhanced aggregation and thrombosis.^13^^,^^49^ In line with earlier findings, a swift reduction in TMRM fluorescence was observed when platelets were exposed to the TXA_2_ synthetic analog, U46619.^50^ Significantly, this effect was reversed when platelets were pre-treated with CDNF, implying that CDNF may directly engage in the downstream signaling pathway of TXA_2_-TXA_2_ receptor (TP), rather than interfering with the catalytic function of COX-1.

In the TXA_2_/TP signal transduction, phosphorylated PLCβ3 hydrolyzes phosphatidylinositol 4,5-biphosphate (PIP_2_) into inositol 1,4,5-triphosphate (IP_3_) and diacylglycerol (DAG).^51^ In the calcium ions environment, DAG acts as a stimulatory cofactor and activates PKC, which subsequently triggers the downstream signaling Rap1b/Rap1-GTP interacting adapter protein to Talin, ultimately leading to the upregulation of integrin αIIbβ3 fibrinogen receptors.^52^^,^^53^ Since the molecular mechanism of CDNF’s action remains elusive, in this study, we employed co-immunoprecipitation of the human CDNF protein and analyzed its interactions in AA-treated platelets using LC-tandem MS (LC-MS/MS). For our interactome screening, we used mammalian tissues/cell lines originating from human species. We report that the CDNF interactome in AA-treated platelets consists of 30 proteins that are associated with actin cytoskeleton and integrin signaling pathways. GO term overrepresentation analysis of the CDNF-interacting proteins revealed platelet aggregation and platelet alpha granule lumen related to the proteins in the CDNF interactome. This indicates that the CDNF-interacting proteins behind these GO terms represent the biological effect of CDNF on platelet activation.

Since LC-MS/MS captures protein complexes, it is unclear how many of the proteins of the CDNF interactome represent proteins interacting with CDNF directly in platelets. Based on the findings of the negative effects of CDNF on the platelet aggregation in our *in vivo* and *in vitro* data, CDNF is more likely to be interacting with aggregation-related proteins, such as Rap1b, ACTB, ACTG1, and TLN1. Rap1 is a soluble cytosolic G-protein that consists of two isoforms, Rap1a and Rap1b, sharing approximately 95% sequence identity.^54^ While Rap1 proteins are ubiquitously expressed in tissues, Rap1b is the predominant Rap1 isoform in platelets. The majority of Rap1b exists in a GDP-bound state within the cytoplasm of platelets. It is only when Rap1b couples with its respective guanine nucleotide exchange factors (GEFs) at the plasma membrane that it transitions into the GTP-bound active form.^55^ The GTP-bound Rap1b active form promotes ERK activation, granule secretion, and unmasking talin-1’s integrin binding site to induce integrin activation crucial for platelet aggregation.^56^ In this work, we demonstrated that extracellularly delivered CDNF could dampen AA-induced Rap1b activation. Importantly, CDNF suppresses the activation of Rap1b in a manner that depends on the concentration of GTPγS that is a non-hydrolysable or slowly hydrolysable G-protein-activating analog of GTP. These findings imply that CDNF, by interacting with Rap1b, either dampens the trafficking of Rap1b to the plasma membrane or hinders the coupling of Rap1b with GEFs, thereby interfering with the exchange of GTP to GDP on Rap1b. Nonetheless, the transcellular propagation of CDNF and the identification of its specific motifs interacting with Rap1b, leading to the downregulation of AA-induced Rap1b activation, require further elucidation.

In addition to acting as an effector of Ca^2+^-induced CalDAG-GEFI in platelet integrin activation, Rap1b has been implicated in the regulation of sarcoendoplasmic reticulum Ca^2+^-ATPases (SERCA) to affect intracellular Ca^2+^ pools.^57^^,^^58^ Recent studies demonstrated that PKA-mediated phosphorylation at serine 179 on Rap1b interferes with the prenylation of Rap1b, leading to decrease its membrane localization, subsequently preventing the coupling of Rap1b with GEFs, but induces SERCA3b activity. This increase in SERCA3b activity enhances the filling state of the SERCA-associated Ca^2+^ ER pool, ultimately leading to platelet inhibition.^59^ This crosstalk between Rap1b and Ca^2+^ signaling may provide an explanation why extracellularly added CDNF, which is shown to interfere with GTP-bound Rap1b active form, can subsequently reduce the cytosolic concentration of Ca^2+^ during the platelet activation process.

Beyond their importance in hemostasis and thrombosis, increasing evidence highlights a pivotal role of platelets for inflammatory and immune responses. More recently, platelets were reported to assemble inflammasomes and may serve as significant cellular sources of cytokines.^60^^,^^61^^,^^62^ In line with our findings, co-culture with platelets has been shown to increase the production of cytokines from innate immune cells.^61^ Crucially, our research revealed that platelets obtained from stroke patients boost production of IL-1β by BV2 cells accompanied by upregulated levels of COX-2 and p-JNK in comparison with co-cultures with platelets from healthy donors. These findings provide further evidence to support that activated platelets have the capacity to skew microglia into a pro-inflammatory phenotype. While earlier studies showcased CDNF’s anti-inflammatory effects on primary glial cells, our study found that co-treatment of CDNF with washed platelets did not mitigate the levels of IL-1β and COX-2 in BV2 microglial cells. Conversely, when CDNF was pre-treated in washed platelets, it effectively dampened the pro-inflammatory phenotypes of BV2 microglial cells and led to a reduction in their production of pro-inflammatory cytokines. These findings reinforce the notion that CDNF, by modifying platelet activation, can prevent the platelet-boosting effects on microglial polarization. However, platelet-induced intracellular signaling in polarization of monocytes or macrophages is still incompletely understood. Also, it remains to be determined whether the influence of platelets on microglia is contingent on direct cell contact or involves platelet-derived nucleic acids, lipid mediators, or purines. Furthermore, the exact manner in which CDNF affects this process requires further investigation.

In correspondence with previous studies, the levels of many oxylipins, e.g., 12-HETE and prostaglandins, were increased after ischemic stroke.^63^ As previous studies have focused on AA (ω:6)-derived metabolites, the elevation of 9-HODE, 9-KODE, 12(13)-EpOME, and 13-HODE formed from linoleic acid (ω:6), 13-HOTrE formed from α-linolenic acid (ALA, ω:3), and 12-HEPE from eicosapentaenoic acid (EPA, ω:3) after ischemic stroke have not been described before. We determined the oxylipin profile in plasma, while we cannot claim the origin of oxylipins directly. However, platelet ALOX12 (platelet 12*S*-LOX), TBXAS1 (TXA_2_ synthase), and PTGS1 (COX-1) have similar protein expression rate,^64^ potentially indicating the origin of the abundant 12-HETE and TXA_2_ in platelets. Furthermore, EPA is a good substrate for platelet 12-LOX and also converts ALA into 12-HEPE and 13-HOTrE, respectively,^65^ explaining the cellular source of those oxylipins. Moreover, pneumonia is one of the common complications of stroke. In our study, post-treatment of CDNF also inhibited the formation of leukotoxins (*cis* and *trans* 12(13)-EpOME) by CYP450 (Figure S8); thus, in future CDNF could be used as a potential therapy to enhance resilience to stroke-induced pneumonia.

There are a few limitations in our study. First, this study utilized the dMCAo model of stroke to recapitulate the process of platelet activation or aggregation observed in stroke patients. However, the pathophysiological mechanisms of activated platelet in animal models may not fully replicate those in human, leading to discrepancies in correlation between platelet activation, disease progression, and response to treatments.^66^ In addition, there is a U-shaped relationship between the dose of CDNF and the effect on AA- or collagen-induced PRP aggregation. The rationale underlying such a dose-response relationship is complex and may be attributed to receptor affinity and selectivity, dual pathway activation, and toxicity at high doses. Since no specific CDNF receptor or dose-limiting toxic effects of CDNF have been identified, we propose that the U-shaped dose-response curve is due to platelet adaptation to CDNF treatment. Therefore, before directly applying the CDNF therapeutic strategy in clinical studies, we must first assess the safety and tolerability of s.c. CDNF injection in stroke patients. Furthermore, we need to establish a platform to illustrate how administered CDNF interacts with platelets and endothelial cells and its potential biological responses to circulating platelets.

The second limitation is that the xenogeneic co-culture of human platelets with murine BV2 microglial cells might trigger xenograft-induced inflammatory responses, although we used healthy platelets as the observed group. While the extracellularly added CDNF effectively mitigated these reactive platelet-induced pro-inflammatory phenotypes of BV2 microglial cells, we cannot exclude the possibility that CDNF may also reduce the inflammatory responses in BV2 cells induced by xenogeneic co-culture. In future studies, we will develop allogeneic co-culture model to evaluate interaction between human microglia and platelets, thereby closely mimicking the pathological microenvironment following ischemic brain injury.

Finally, our work challenges these findings with an animal model of ischemic stroke that demonstrate systemic administration of CDNF, by attenuating platelet activation, aggregation responses, and TXA_2_ production, it therefore mitigates ischemic-induced brain injury and neuroinflammation. In our previous study, CDNF was identified to promote hematoma-scavenging functions of brain myeloid cells to accelerate resolution of hematoma after collagenase-induced ICH.^22^ While the bioavailability of exogenous CDNF does not extend beyond 6 h, it has the potential to facilitate myeloid cells acquiring CD36 and CD163 expression, which are crucial in hematoma clearance and tissue repair processes.^22^ Myeloid cells encompass a variety of cell types and, among them, platelets function as regulators in both hemostasis and immune responses. In the context of vascular injury, platelet GPIa and GPVI receptors bind to exposed subendothelial collagen to stabilize thrombus formation.^67^ In addition to these classic roles, GPVI-mediated thrombo-inflammation is a key player in the neuronal damage that occurs following cerebral reperfusion in the MCAo model of stroke.^2^ The delivery of anti-GPVI antibody, JAQ1, was shown to mitigate inflammatory response, possibly by reducing IL-1β and polyphosphates and decreasing inflammatory cell recruitment after ischemic stroke.^68^ The blocking of platelet GPVI and GPIb was demonstrated to decrease ischemic-induced BBB hyperpermeability through inactivation and downregulation of macrophage antigen-1 (MAC-1) and P-selectin.^69^^,^^70^ In addition to upregulating scavenger receptor expression within the lesioned cortex, as previously observed, systemic administration of CDNF resulted in the downregulation of GPVI/P-selectin receptor expression in PRP, inhibited TXA_2_ synthesis in circulating platelets, and consequently reduced the production of pro-inflammatory cytokines after dMCAo. These findings reinforce the idea that CDNF has the capability to attenuate both thrombotic and inflammatory processes, which leads to reduced infarct area, mitigates BBB disruption, and promotes neurofunctional recovery following ischemic stroke. Although we still need to elucidate whether this anti-inflammatory effect is a consequence of regulating platelet activation or a mechanism involved in cytoprotection to provide a permissive microenvironment, the effectiveness of systemic administration without significant untoward effects offers potential for CDNF in clinical applications.

In conclusion, we discovered that CDNF functions as a hamper of platelet activation, aggregation, and the ensuing thrombo-inflammation that typically follows a stroke insult. These effects are made possible through CDNF’s direct binding with Rap1b, which subsequently suppresses the activation of downstream signaling molecules such as ERK and cPLA_2_ phosphorylation, as well as the production of TXA_2_. In addition, our study offers innovative avenues for tackling the excessive activation of platelets, overproduction of oxylipins, and the resulting inflammatory responses triggered by stroke. Considering these findings, CDNF emerges as a promising therapeutic candidate that could complement existing treatments in mitigating platelet-mediated thrombosis and inflammation processes, which, in turn, could reduce secondary brain injury and promote functional recovery in patients afflicted with ischemic stroke.

## Materials and methods

### Ethics approval and experimental design

Subjects were recruited through Tri-Service General Hospital and National Defense Medical Center. Ethics approval was obtained by Institutional Review Board of Tri-Service General Hospital (project no. B202105006). For this study, inclusion criteria for the subjects of this project involved any patients who suffered from a confirmed spontaneous intracerebral hemorrhage (hemorrhagic stroke) or traumatic intracerebral hematoma (traumatic hemorrhage). Subjects who were diagnosed with hemorrhagic brain tumor or arteriovenous malformation bleeding were excluded from this study. Blood samples were collected from the subjects' arm using phlebotomy and venous techniques within 24 h after either hemorrhagic stroke or traumatic brain injury. CDNF protein expressions in PRP extracted from whole blood of patients and their respective age-matched control were measured by using a human CDNF Elisa Kit (ab260071, Abcam) according to the manufacturer’s protocol.

### Production of recombinant CDNF proteins (drugs)

Recombinant human CDNF, obtained from Icosagen (cat. no. P-100-100, Tallinn, Estonia), was used in this study. The CDNF was produced in mammalian cells, and its biological activity was validated through testing on cultured sympathetic and dopamine neurons,^36^ as well as by evaluations in animal models of Parkinson’s disease^18^ and stroke.^22^

### Preparation of human platelets

The use of healthy human blood samples was approved by Tri-Service General Hospital Research Ethics Committee (project no. C202105122). Blood was collected into sodium citrate tubes (0.32% final) by venipuncture from healthy donors and stroke patients. PRP was obtained by centrifuging the whole blood (100 × *g*, 15 min, 25°C), and platelet-poor plasma was obtained by centrifuging the fraction of red blood cell (10,000 × *g*, 3 min, 25°C). Washed platelets were prepared by further centrifuging PRP (500 × *g*, 10 min, 25°C) and resuspended in modified Tyrode’s buffer (20 mM HEPES, 134 mM NaCl, 2.9 mM KCl, 0.34 mM Na_2_HPO_4_, 12 mM NaHCO_3_, 1 mM MgCl_2_, 0.1% glucose, and 0.35% bovine serum albumin [pH 7.4]) with apyrase (0.02 U/mL) and prostaglandin I_2_ (1 μg/mL). Washed platelets were then adjusted to 2 × 10^5^ platelets/μL and allowed to rest for at least 1 h.

### Preparation of rat platelets

The use of animal blood samples was approved by the Institutional Animal Care and Use Committee of the National Defense Medical Center, Taiwan, R.O.C. (protocol no. 16-258). Rat blood samples were collected into acid citrate dextrose solution (1.5 mM citric acid, 8.5 mM sodium citrate, and 13.6 mM dextrose) by cardiac puncture. PRP was obtained by centrifuging the whole blood (200 × *g*, 15 min, 25°C), and platelet-poor plasma was obtained by centrifuging the fraction of red blood cells (10,000 × *g*, 3 min, 25°C). Washed platelets were prepared by further centrifuging PRP (700 × *g*, 10 min, 25°C) and resuspended in modified Tyrode’s buffer (20 mM HEPES, 134 mM NaCl, 2.9 mM KCl, 0.34 mM Na_2_HPO_4_, 12 mM NaHCO_3_, 20 mM HEPES, 1 mM MgCl_2_, 0.1% glucose, and 0.35% bovine serum albumin [pH 7.4]) with apyrase (0.02 U/mL) and prostaglandin I_2_ (1 μg/mL). Washed platelets were then adjusted to 3 × 10^5^ platelets/μL and allowed to rest for at least 1 h.

### Measurement of platelet aggregation

The 96-well plate aggregometry method was used to assess platelet aggregation according to a previous study.^71^ In brief, platelet samples were placed into individual wells of a 96-well plate and activated by adding different concentrations of ADP (0.3–30 μM), epinephrine (10^−8^ to 10^−4^ M), thrombin receptor activator peptide SFLLRN (TRAP-6) (0.3–30 μM), collagen (0.1–10 μg/mL), or AA) (0.01–0.6 mM). The 96-well plate was then shaken at 1,200 rpm for 5 min at 37°C by a microplate shaker (BioShake IQ, Quantifoil Instruments, Germany) and the absorbance at 595 nm detected using a multiplate spectrometer (PowerWare 340; BioTek, Winooski). Platelet aggregation percentage was analyzed with reference to the absorbance of PPP or modified Tyrode’s buffer as a surrogate for 100% aggregation.

### Flow cytometry

Flow cytometry was used to investigate the expression of GPVI, P-selectin, and TMRM on platelets. In brief, isolated platelets from stroke patients were incubated with anti-GPVI-APC (1:100– Invitrogen) or anti-CD61-PerCP (1:150– BD Bioscience) for 30 min in the dark and fixed with 0.1% formaldehyde. The labeled platelets were then analyzed using the flow cytometer (FACSCanto II, BD Bioscience, CA). In addition, PRP or washed platelets were stimulated with AA (0.1–0.6 mM) after incubation (15 min) with CDNF (0.01–1 μg/mL). The platelet samples were then labeled with anti-P-selectin-PE (1:100 for human sample– BD Biosciences), anti-TMRM-PE (1:100 for human sample– BD Invitrogen), anti-CD61-PerCP (1:150 for human sample– BD Biosciences), anti-P-selectin-PE (1:100 for animal sample– Bio-Rad), or anti-CD61-FITC (1:100 for animal sample– Invitrogen) for 30 min in the dark and fixed with 0.1% formaldehyde. Expression of P-selectin and TMRM on platelets was measured using a flow cytometer (FACSCanto II, BD Bioscience, CA).

### Dye loading and measurement of calcium flux

For the measurement of calcium flux, 100 μL of washed platelets was incubated with 1 μL of Fluo-4AM dye (Fluo-4, AM, cell permeant Invitrogen Fluo-4, AM, cell permeant F14201) in the Nunc FluoroNunc 96-well plate at room temperature for 1 h. During this incubation time, CDNF (1 μg/mL) or PBS could be added to the plate. After a 15-min drug incubation, an additional 10 μL of thrombin was introduced to each well, resulting in a final concentration of 1 U. This step was performed to induce platelet activation in the experimental samples. Subsequently, the plate was shaken at 700 rpm for 1 min. Fluorescence measurements (excitation 345 nm/emission 494 nm) were analyzed using the CLARIOstar Plus 96-well fluorescence spectrum reader, with readings taken every 5 s for a total duration of 240 s. The time courses of fluorescence measurements can be presented as normalized to the basal fluorescence values acquired before the stimulation, represented as F/F0. This normalization method allows for the comparison of changes in fluorescence intensity relative to the initial baseline, enabling a clearer visualization and analysis of the effects of the stimulation on the experimental samples.

### Platelet superoxide detection

Chemiluminescence (CL) was assessed using luminometers (Hidex Chameleon luminometers-LSC Microplate Reader) and LumiNunc 96-well plates were used in this assay. Washed platelet (2 × 10^8^/mL) were pre incubated with PBS or CDNF for 15 min. ROS was recorded by peak height, corrected for spontaneous CL, and time to peak was analyzed. Readings were started immediately on addition of agonists and reagents and were continuous for up to 10 min (before read, shaking 700 rpm before each cycle). Lucigenin at a final concentration of 250 μM was added immediately before the analysis.

### Co-immunoprecipitation and immunofluorescence

Human platelets treated with CDNF or with PBS were lysed in pre-cooled lysis buffer containing protease inhibitor at room temperature for 10 min, and then centrifuged at 12,000 × *g* for 10 min to collect the supernatant. Immunoprecipitation was performed using magnetic beads and a Pierce MS-Compatible magnetic IP Kit (Pierce, Rockford, IL) according to the manufacturer’s protocol. In brief, the cell lysates (2.4 mg) were incubated with protein A/G-conjugated agarose beads at 4°C for 1 h to avoid nonspecific binding. Thereafter, the lysate was reacted with rabbit monoclonal human CDNF antibody (4.8 μg, ab253138, Abcam) or IgG (4.8 μg, ab276304, Abcam) at 4°C overnight, and then with protein A/G-conjugated agarose beads for 3 h to absorb the antibodies. The beads were washed and loaded into the loading buffer and boiled for 5 min to elute the protein and subjected to western blot analysis. For immunofluorescent staining, 2 × 10^5^ human washed platelets/μL was plated into a 12-well fibrinogen-coated plate and incubated for 10 min before drug treatments. CDNF was labeled with Alexa Fluor 647 dye using a Microscale Protein Labeling Kit (A30006, Invitrogen) according to the manufacturer’s instructions. The Alexa Fluor 647 dye-labeled CDNF (3 μg/100 μL) was administered in the washed platelet culture. After incubation at 37°C for 30 min, the unengulfed CDNF were removed by washing with Dulbecco’s PBS 3 times, followed by double-staining washed platelets with phalloidin (1:1,200, FITC) and Rap1b (1:200– Cell Signaling Technology, 8825S). Then, CDNF-labeled washed platelets were observed by fluorescent microscopy (THUNDER Imaging Systems).

### Rap1 activity assay

Rap1 pull-down assays were performed using the Rap1 activation kit (ab212011, Abcam) according to the manufacturer’s instructions with modifications. In brief, human platelets were lysed with the buffer contained in the activation assay kits. After lysis on ice and centrifugation at 12,000 × *g* for 10 min at 4°C, supernatants were collected. For affinity precipitation of activated Rap1, 1.8 mg of cell lysates was incubated with 50 μL of glutathione resin slurry in the presence of 10 μg of RalGDS-RBD protein for 1 h at 4°C. The beads were pelleted and washed three times with lysis buffer. The beads were finally resuspended in 40 μL of 2× sodium dodecyl sulfate (SDS) sample buffer and boiled for 5 min. Whole-cell lysates and pull-down samples were subjected to western blotting, utilizing antibodies specific to Rap1 for detection.

### Cell culture and treatment

Murine BV-2 microglial cells were gifted from Dr. Mei-Jen Wang^72^ and maintained in Dulbecco’s modified Eagle’s medium containing 5% fetal bovine serum, 2 mM L-glutamine, 100 μg/mL streptomycin, and 100 U/mL penicillin (all from Gibco, Thermo Fisher Scientific, Waltham, MA) at 37°C under humidified 95% O_2_ and 5% CO_2_. The cells were then seeded into 24-well plates at a density of 1 × 10^5^/well and maintained at 37°C under humidified 95% O_2_ and 5% CO_2_. Human washed platelets (WPs) were pretreated with 1 mg/mL CDNF or PBS (as vehicle control) for 30 min. Then, PBS-treated or CDNF-treated WPs (3 × 10^7^ platelets) were co-cultured with BV2 cells (1.67 × 10^6^/mL) at 37°C for 6 h.

### Western blot analysis

Total protein was extracted using RIPA lysis buffer containing protease and phosphatase inhibitor cocktail (Abcam, Cambridge, Cambs, UK). Protein concentrations were determined by Bradford assay (Bio-Rad, Hercules, CA). Proteins (30 μg) were separated by 12% SDS-PAGE and transferred onto polyvinylidene fluoride (PVDF) membranes. After blocking with 5% non-fat milk in Tris-buffered saline with 0.1% Tween 20 (TBST) for 0.5 h at room temperature, the membrane was incubated with antibodies against i-NOS (1:500, GeneTex, GTX130246), COX-2 (1:500, GeneTex, GTX100656), IL-1β (1:500, Santa Cruz, SC-12742), IL-6 (1:1,000, Santa Cruz, SC-57315), TNF-α (1:1,000, Santa Cruz, SC-12744), IL-10 (1:500, Santa Cruz, SC-32815), CD36 (1:500, Novus, NB400-144), CD206 (1:500, Santa Cruz, sc-376232), CD163 (1:250, Abcam, ab182422), p-P38 (1:500, Cell Signaling Technology, 9211S), p-JNK (1:500, Cell Signaling Technology, 9251S), P38 (1:1,000, Cell Signaling Technology, 9212S), JNK (1:1,000, Cell Signaling Technology, 9252S), p-Akt (1:500, Cell Signaling Technology, 9217S), Akt (1:1,000, Cell Signaling Technology, 9272S), p-ERK (1:1,000, Cell Signaling Technology), ERK (1:2,000, Cell Signaling Technology), and GAPDH (1:1,000, Abcam, ab8245) at 4°C overnight. After 3 times washes with TBST, the blots were incubated with HRP goat anti-rabbit IgG (H + L) (GeneTex, Irvine, CA) or HRP-labeled goat anti-mouse IgG (H + L) (GeneTex) for 1 h at room temperature. The bands were visualized using the enhanced CL (ECL) reagents (Thermo Scientific, Rockford, IL) and were quantified with ImageJ software. The protein expression levels were expressed as the percentage of GAPDH. The western blots were captured with a digital camera, and the intensities were quantified with NIH ImageJ.

### Network and protein interaction analysis

The differentially expressed proteins, which were included with the most significant signal pathway (*p* < 0.001), were selected for network analysis by Ingenuity Pathway Analysis (IPA) software (QIAGEN, USA). The IPA program (QIAGEN’s Ingenuity Pathway Analysis, IPA software, Redwood City, https://www.qiagen.com/ingenuity) included the Ingenuity Pathway Knowledge Base, which is organized to analyze protein function and interaction. After joining 31 proteins into My Pathway tool, a network of 31 proteins were connected to each other by the IPA database, and each protein was labeled with a different color by gene expression level (up with red, down with green, and no expression level with white). Different shapes of genes were indicated different function in cell.

### Animals

A total of 157 male adult Sprague-Dawley rats (300–350 g; 36 sham, 23 sham + CDNF, 50 dMCAo + vehicle, 48 dMCAo + CDNF), based on previous experience involving similar experiment settings, were used for this study. Those animals were housed at the National Defense Medical Center’s Animal Center with a 12-h light/dark cycle, temperature of 25°C ± 2°C, 55% humidity, two to four animals per cage, and *ad libitum* standard diet and water. The experimental protocol was approved by the Institutional Animal Care and Use Committee (protocol no. 16-258) of the National Defense Medical Center, Taiwan, R.O.C., which is accredited by the Association for Assessment and Accreditation of Laboratory Animal Care International. All experiments were performed in a blinded manner, and the experimental results are reported according to the ARRIVE guidelines.

### Distal middle cerebral artery occlusion

Each rat underwent a cortical stroke induction by occluding the distal middle cerebral artery (dMCA) and bilateral common carotid arteries (CCAs).^73^ Anesthesia was induced using 4% chloral hydrate injected intraperitoneally at a dose of 400 mg/kg, and lidocaine was used as a local anesthetic. The surgical procedure was conducted as previously described. In brief, the CCAs were separated via cervical dissection, followed by a small craniotomy on the right side of the skull to ligate the right dMCA directly using a 10-0 suture. The CCAs were occluded simultaneously with non-traumatic arterial clips. After 90 min of ischemia, the suture around the MCA and the arterial clips were removed to restore blood flow (reperfusion). Throughout the procedures and until recovery from anesthesia, each rat’s body temperature was maintained at 37°C, after which the rat was returned to its home cage.

### Ultra-performance LC-MS analysis of oxylipins

The use of animal blood samples was approved by (ESAVI/13959/2019). Rat blood samples were collected into 3 mL K2EDTA tubes (BD Vacutainer) by cardiac puncture, centrifuged at 2,000 × *g* for 15 min at 4°C and plasma stored at −80°C in aliquots. For oxylipin extraction, rat plasma samples were thawed at 4°C for 30 min, 95 μL plasma was spiked with 10 μL oxylipin internal standard mixture (Cayman Chemical/Larodan, in-house prepared), and solid-phase extraction was performed on an HLB Oasis cartridges (Water, Milford) using an Extrahera Classic automated extraction system (Biotage, Uppsala, Sweden) according to the manufacturer’s instructions. Oxylipins were eluted from the cartridge with LC-MS methanol, dried extracts were reconstituted in MeOH/H_2_O (6:1, v/v), filtered and stored at −80°C until sample analysis (maximum 2 days). Samples were analyzed on an ACQUITY UPLC System (with an ACQUITY BEH C_18_ 2.1 × 100 mm column with a 1.7 μm particle size), coupled to a Waters Xevo TQ-S triplequadrupole system (Waters, Milford, MA), equipped with an electrospray ion source operating in the negative ion mode. Run with the solvent system of A (0.1% acetic acid in Milli-Q water) and B (acetonitrile/isopropanol 90:10, v/v): 0–2.1 min 35% B, 2.1–3.5 min 40% B, 3.5–5.5 min 42% B, 5.5–11.5 min 50% B, 11.5–11.7 min 72.5% B, 11.7–13.5 min 100% B, 13.5–13.6 min 35% B, 13.6–15 min 35% B at a flow rate of 425 μL/min. Identity of compounds was confirmed by matching retention times and at least one selected reaction monitoring transition with oxylipin standards.^74^

### Functional neurobehavioral tests

Assessment of neurological abnormalities by a mNSS test (normal score, 0; maximal deficit score, 18) and cylinder test were carried out as described.^21^^,^^75^ Evaluation was performed by an investigator blinded to the experimental treatment scheme. The mNSS is a composite test of motor, sensory, and balance functions. The assessment was performed on day 2 post-dMCAo. The body asymmetry test was conducted following established protocols.^76^ In this test, the rats were lifted above the testing table by their tails, and the frequency of initial turnings of the head or upper body contralateral to the ischemic side was counted. This procedure was repeated 20 times in total. Neurological deficits in all rats were assessed using the modified Bederson’s score.^77^ The cylinder test, as described in previous studies,^78^ was also performed. The rats were placed inside a clear plastic cylinder with a diameter of 35 cm and observed for 5 min. The number of times the rats raised their front paws to touch the inner wall of the cylinder was counted. Locomotor activity was measured using an infrared activity monitor for 1 h (Med Associates, St. Albans, VT).

### BBB disruption with Evans blue extravasation

The integrity of the BBB was assessed using a modified Evans blue extravasation method before sacrifice.^75^ In brief, 72 h post-dMCAo, rats were anesthetized with sodium pentobarbital (50 mg/kg, i.p.) and infused via the right femoral vein with 37°C Evans blue dye (2% in 0.9% normal saline, 4 mL/kg) over a period of 5 min. After a 2-h interval, the rats were perfused with 300 mL of normal saline to remove any remaining dye from the blood vessels. The brains were then removed and sectioned to a thickness of 2 mm using a rodent brain matrix. Coronal brain sections were taken from +2 to −2 mm from bregma. The evaluation of BBB permeability was performed in the ipsilateral and contralateral striatum, as well as the cerebellum, with the cerebellum serving as an internal control. Each section was immediately weighed and homogenized in 1 mL of 0.9% normal saline. To precipitate proteins, 1 mL of 60% trichloroacetic acid solution was added, and the mixture was vortexed for 2 min. After cooling for 30 min, centrifugation was carried out at 1,500 × *g* and 4°C for another 30 min. The absorbance of Evans blue in the supernatant was then measured at 610 nm using a spectrophotometer (Molecular Devices OptiMax). Dye concentration was expressed as μg/g of tissue weight and calculated based on a standard curve obtained from known amounts of dye.

### Immunoblotting of brain tissue

The area composed of the peri-infarct cortex extending 1 mm from the infarction border was collected and rapidly frozen in liquid nitrogen at −80°C until further analysis. In brief, equal amounts of protein (20 μg) were loaded onto 10% (SDS-PAGE) gel and then electrophoresed, followed by blotting of protein onto a PVDF membrane (Immobilon-P PVDF membrane, Millipore, Bedford, MA) blocked with 5% non-fat milk in 0.05% Tween-Tris-buffered saline. The membrane was probed with primary antibodies overnight at 4°C with gentle rotation. Primary antibodies and concentrations were used as follows: iNOS (1:500, GeneTex, GTX130246), CD163 (1:250, Abcam, ab87099, ab182422), CD91 (1:1,000, Abcam, ab92544), CD36 (1:500, Novus, NB400-144), GAPDH (1:1,000, Abcam, ab181602), IL-10 (1:500, Santa Cruz, SC-32815), CD11b (1:1,000, GeneTex, GTX134493), TNF-α (1:500, Santa Cruz, SC-12744), IL-1β (1:500, Santa Cruz, SC-12742), ZO-1 (1:250, Santa Cruz, SC-33725), Claudin-5 (1:500, Invitrogen, 35–2500), and MMP-9 (1:250, Abcam, ab19016). Following washing and incubation with respective secondary antibodies (AP307P and AP308P; EMD Millipore, Billerica, MA, 1:20,000) for 1 h at room temperature, membranes were then reacted with chemiluminescent ECL Plus western blotting detection system (RPN2133; Amersham Biosciences, Little Chalfont, UK). Bands were detected by exposure to X-ray films (34091; Kodak, Rochester, NY). Band intensities were quantified using densitometric analysis (GS-800 Calibrated Densitometer, Bio-Rad, Hercules, CA) and calculated as the optical density × area of the band.

### Statistical analysis

All graphs and statistics were performed in GraphPad Prism 10. The results of lesion volume, Evans blue extravasation, ROS lucigenin intensity, Fluo-4 fluorescent amplitude, GPVI expression, western blot of tight junction proteins, and pro-inflammatory/anti-inflammatory mediators, were analyzed using the two-tailed Student’s t test. The statistical comparisons among multiple groups were made using one-way ANOVA, and multiple time points by two-way ANOVA were followed by Holm-Šidák, Bonferroni, or Dunnett’s multiple comparisons post hoc test. In all instances, *n* refers to the number of animals in a particular group. A *p* value of <0.05 is considered statistically significant.

## Data and code availability

The dataset(s) supporting the conclusions of this article is(are) included within the article (and its additional file(s)).

## Acknowledgments

We acknowledge the Karolinska Institute Small Molecule Mass Spectrometry Core Facility (KI-SMMS) for support in the sample analyses. Supported by the 10.13039/501100004663Ministry of Science and Technology of Taiwan, ROC, MOST 108-2314-B-016 -058-MY2, MOST 107-2314-B-303-022, and MOST 110-2314-B-016-034; Medical Research Project grants TSGH-E110-213 from the 10.13039/501100010425Tri-Service General Hospital of Taiwan; the 10.13039/100022871National Defense Medical Center. 10.13039/501100006306Sigrid Jusélius Foundation, and Neuroscience Center, HiLIFE, UH (to M.A. and H.L.). 10.13039/501100002341Academy of Finland mobility fellowship grant 323858 (to M.A. and H.K.L.). 10.13039/501100002341Academy of Finland 322757 (to H.L.). 10.13039/501100004012Jane and Aatos Erkko Foundation and 10.13039/501100002341Academy of Finland 1310891 (to M.S.).

## Author contributions

Conceptualization, K.-Y.T. and M.A.; performance of experiments, H.L., H.-K.L., K.-Y.T., V.W., W.-F.H., J.-S.W., and C.-C.S.; data analysis, J.-S.W., H.L., Y.-H.C., H.-K.L., K.-Y.T., and C.-C.S.; writing – original draft, K.-Y.T., J.-S.W., C.-C.S., H.L., and M.A.; guidance, Y.-H.C. and M.S.

## Declaration of interests

M.S. is one of inventors of the CDNF-related patent (7452969), which is owned by the Herantis Pharma Company (Espoo, Finland).

## Supplemental information

Document S1. Figures S1‒S10, Tables S1‒S3, and supplemental materials

Document S2. Article plus supplemental information
