# Supplementary material for: Targeting Rap1b signaling cascades with CDNF: Mitigating platelet activation, plasma oxylipins and reperfusion injury in stroke
Source: Mol Ther. 2024 Sep 10;32(11):4021–44. doi: 10.1016/j.ymthe.2024.09.005 (PMC11573613; doi:10.1016/j.ymthe.2024.09.005)
Supplement: Document S1. Figures S1‒S10, Tables S1‒S3, and supplemental materials [file mmc1.pdf]

YMTHE, Volume *32*

Supplemental Information

Targeting Rap1b signaling cascades with CDNF: Mitigating platelet activation, plasma oxylipins and reperfusion injury in stroke

Jui-Sheng Wu, Helike Lõhelaid, Chih-Chin Shih, Hock-Kean Liew, Vicki Wang, Wei-Fen Hu, Yuan-Hao Chen, Mart Saarma, Mikko Airavaara, and Kuan-Yin Tseng

Supplemental Materials

Protein in-solution digestion and peptide TMT labelling

The samples were reduced with 10 mM dithioerythreitol in 25 mM ammonium bicarbonate and 8M urea in 25 mM ammonium bicarbonate, pH 8.5, at 37 °C for 1 hr, and subsequently alkylated with 25 mM iodoacetamide in 25 mM ammonium bicarbonate, pH 8.5, at room temperature in dark for 1 hr. Then the reaction was quenched with 25 mM dithioerythreitol in 25 mM ammonium bicarbonate. The samples were digested with 1:50 enzyme to protein ratio of mass spectrometry grade Lys-C (Wako, 125-05061) in 25 mM ammonium bicarbonate, pH 8.5, digested at 37 °C for 3 hr with urea concentration lower than 4M. Following Lys-C digestion, same amount of sequencing grade trypsin (Promega, Madison, WI, USA) in 25 mM ammonium bicarbonate, pH 8.5, was added and digested at 37 °C for 16 hr while urea concentration was lower than 1M. The digestion reaction was quenched with 0.1% formic acid then dried in Speedvac. The peptide mixture was aliquoted, desalted, and concentrated on a C18-ZipTip (Millipore), and eluted with 50 % acetonitrile in 0.1% formic acid. Peptide samples were dried with Speedvac and 2 μg of each peptide samples were re-suspended in 100 μL 100mM TEAB. TMT10plex reagent (Thermofisher, #90111) were re-suspended in 41 μl anhydrous ACN. The appropriate amount of TMT reagent was added to each sample, mixed, and incubated for 1 hr at room temperature with periodic mixing. The labeled samples were quenched with 8 μl of 5% hydroxylamine. Each labeled sample were combined with equal volume and desalted on a C18-ZipTip (Millipore).

Liquid chromatography - mass spectrometry and proteome discoverer analysis

NanoLC−nanoESI-MS/MS analysis was performed on an EASY-nLC™ 1200 system connected to an Thermo Orbitrap Fusion Luoms mass spectrometer (Thermo Fisher

Scientific, Bremen, Germany) equipped with a Nanospray Flex™ ion source (Thermo Fisher Scientific, Bremen, Germany). Peptide mixtures were loaded onto a 75 μm ID, 25 cm length PepMap C18 column (Thermo Fisher Scientific) packed with 2 μm particles with a pore with of 100 Å and were separated using a segmented gradient in 120 min from 5% to 45% solvent B (80 % acetonitrile with 0.1 % formic acid) at a flow rate of 300 nl/min. Solvent A was 0.1% formic acid in water. The mass spectrometer was operated in the data-dependent multi-notch synchronous precursor selection (SPS) method. Briefly, survey scans of peptide precursors from 400 to 1600 m/z were performed at 120K resolution with a 2×105 ion count target. Tandem MS was performed by isolation window at 0.7 Da with the quadrupole, CID fragmentation with normalized collision energy of 35, and rapid scan MS analysis in the ion trap. The MS2 ion count target was set to 1×104 and the max injection time was 35 ms. Only those precursors with charge state 2–6 were sampled for MS2. The instrument was run in top speed mode with 3 s cycles; the dynamic exclusion duration was set to 15 s with a 10-ppm tolerance around the selected precursor and its isotopes. Monoisotopic precursor selection was turned on. The MS3 scans were done by isolating the 10 most intense fragment ions from CID fragmentation over a 400–1600 m/z range, excluding 50 Da below and 5 Da above the precursor ion. These fragment ions were sent to the HCD cell using the SPS waveform with an isolation width of 2.0 Da. HCD fragmentation with normalized collision energy of 55, and MS3 scan analysis at 30K resolution in the orbitrap. The MS3 ion count target was set to 1.5×105 and the max injection time was 86 ms. The MS and MS/MS raw data were processed by Proteome Discoverer (v 2.5.0.400; Thermo Scientific, Waltham, MA, USA) and searched against Swiss-Prot protein sequence database and cRAP contaminate database with the Mascot

server (v.2.8.0; Matrix Science, Boston, MA, USA). Taxonomy was set as *Homo sapiens.* Search criteria used were trypsin digestion, static modifications set as carbamidomethyl (C) and TMT10plex (K & N-Term), variable modifications set as oxidation (M) and Acetylation (Protein N-term), allowing up to 2 missed cleavage, mass accuracy of 10 ppm for the parent ion and 0.6 Da for the fragment ions mass tolerance. Reporter ion quantification was based on MS3 spectrum and performed without normalization. Protein ratio was calculated by Proteome Discoverer with protein abundance-based calculation. Proteins with single peptide hit were removed. The relative quantification of protein was based on the sum of peptide peak area, detected using Minora algorithm. The differential proteins were selected using mean ± 2×SD of log2 transformed ratio as cut-off value. The LC-MAS data have been deposited in the peptide database available via ProteomeXchange with identifier PXD047721.

Bioinformatics

The differential proteins were then determined using threshold: mean of log2 transformed ratio ± 2 × standard deviation of log2 transformed ratio, assuming normalized ratio distributed normally. The Gene ontology and Pathway of differential proteins were then enriched using DAVID database (https://david.ncifcrf.gov).

Evaluation of physiological parameters

Under urethane (1.0 g/kg body weight, i.p., Sigma-Aldrich) anesthesia, a femoral artery was cannulated with a PE-50 polyethylene tube for fluid supplementation and monitoring of arterial blood pressure and blood gas. Arterial blood pressure and heart rate were recorded through an amplifier (MP36, BIOPAC system, CA, USA) and stored in a PC. Body temperature (rectal temperature) was automatically maintained at 37.5 ± 0.5°C by a

rectal temperature sensor and a heating pad (CMA-150, Sweden). Blood cell counts including white blood count (WBC), hemoglobin (Hgb), platelets, neutrophil, lymphocyte, monocyte, Eosinophil and Basophil were measured 10 minutes pre- (Pre-treatment/operation) and 2, 3, and 6 hours post-s.c. CDNF injection or 6, and 24 hours post-dMCAo.

Statistical analysis

Values are presented as mean ± S.E.M. Unpaired t-test, and one-or two-way analysis of variance (ANOVA) with post hoc Bonferroni tests were used for statistical analysis. A statistically significant difference was defined as $p<0.05$.

Supplemental Figures

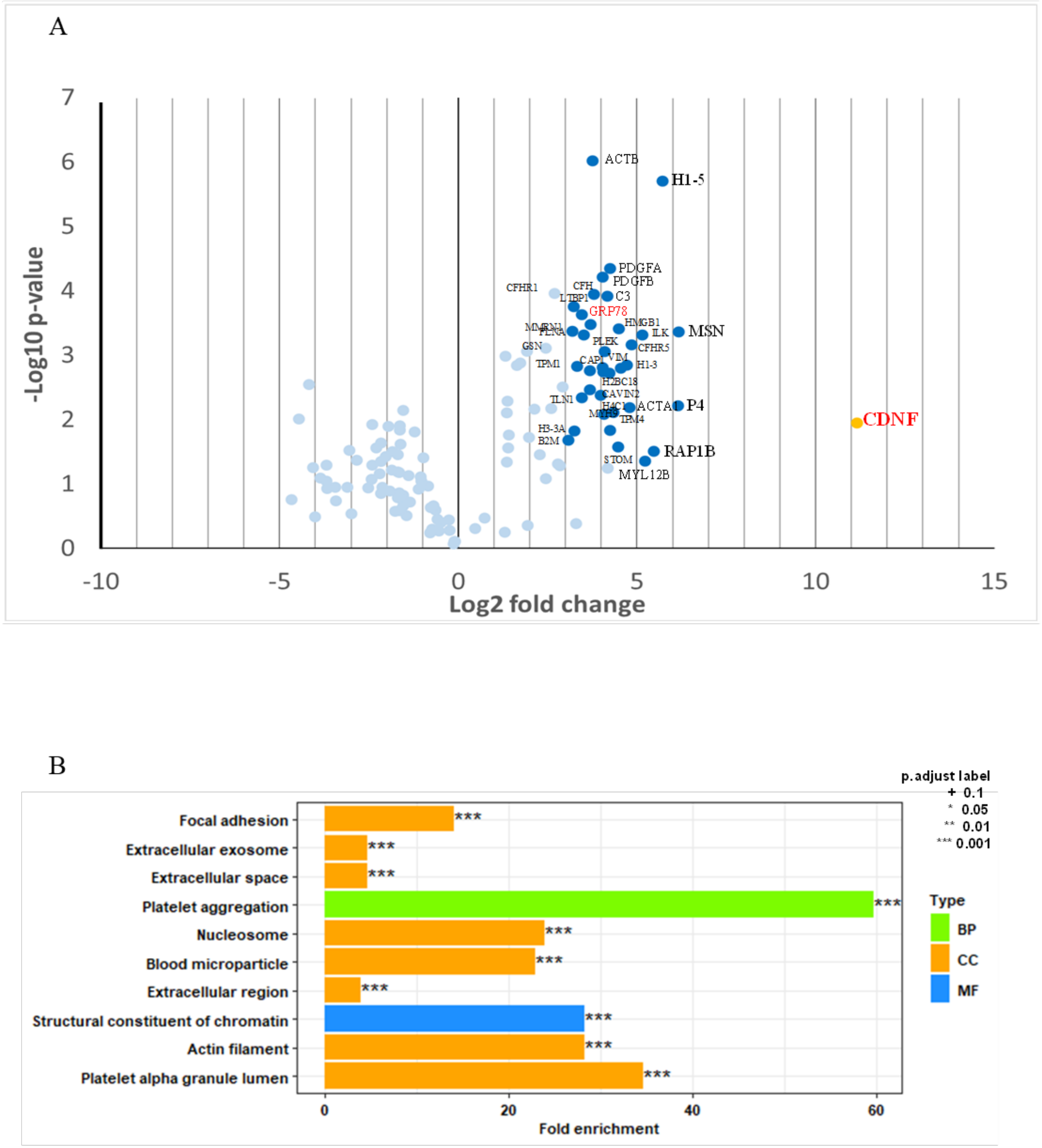

Fig. S1. **The CDNF interactome in CDNF-treated platelets in the presence of AA.** Volcano plot of peptide expression profiles in washed platelets, showing distribution of significance [-log10(P value)] vs. fold change [log2(fold change)] for all proteins. IgG-immunoprecipitated proteins from CDNF- and AA-treated platelets in LC-MS/MS peptide

data serves as negative controls. (A) CDNF interactomes consisting of significantly enriched proteins are shown as blue dots, indicating significantly differentially expressed peptides compared to negative control (log2fold change >3; p-value < 0.05). GO biological processes/cellular components/Molecular functions overrepresentation analysis of CDNF interaction from CDNF-treated platelets in the presence of AA. The top 10 significantly overrepresented (P adjust value < 0.05) terms are listed in the fold enrichment values.

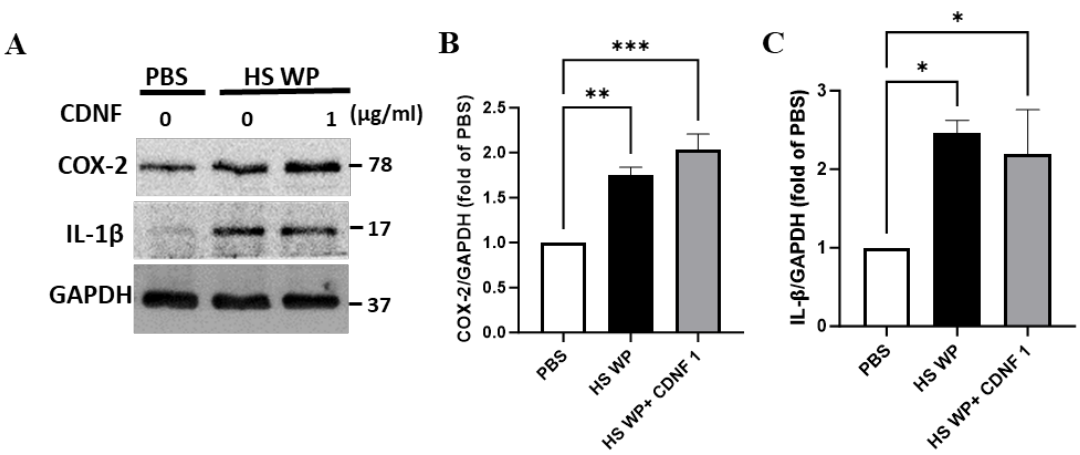

Fig. S2. **Co-treatment of CDNF and stroke patient's platelets still induces the inflammatory responses in BV2 microglial cells. (A)** Lysates from BV2 cells treated with PBS or stroke patient's platelets in the presence or absence of CDNF were immunoblotted to analyze the levels of COX-2, IL-1β, and GAPDH. (B-C) Protein levels of COX-2 and IL-1β were quantified in relation to levels of GAPDH, a housekeeping protein (n = 3, mean ± S.E.M). * p<0.05, ** p < 0.01, *** p < 0.001 by Tukey's multiple comparisons test, following one-way ANOVA. HS WP: Hemorrhagic stroke washed platelets.

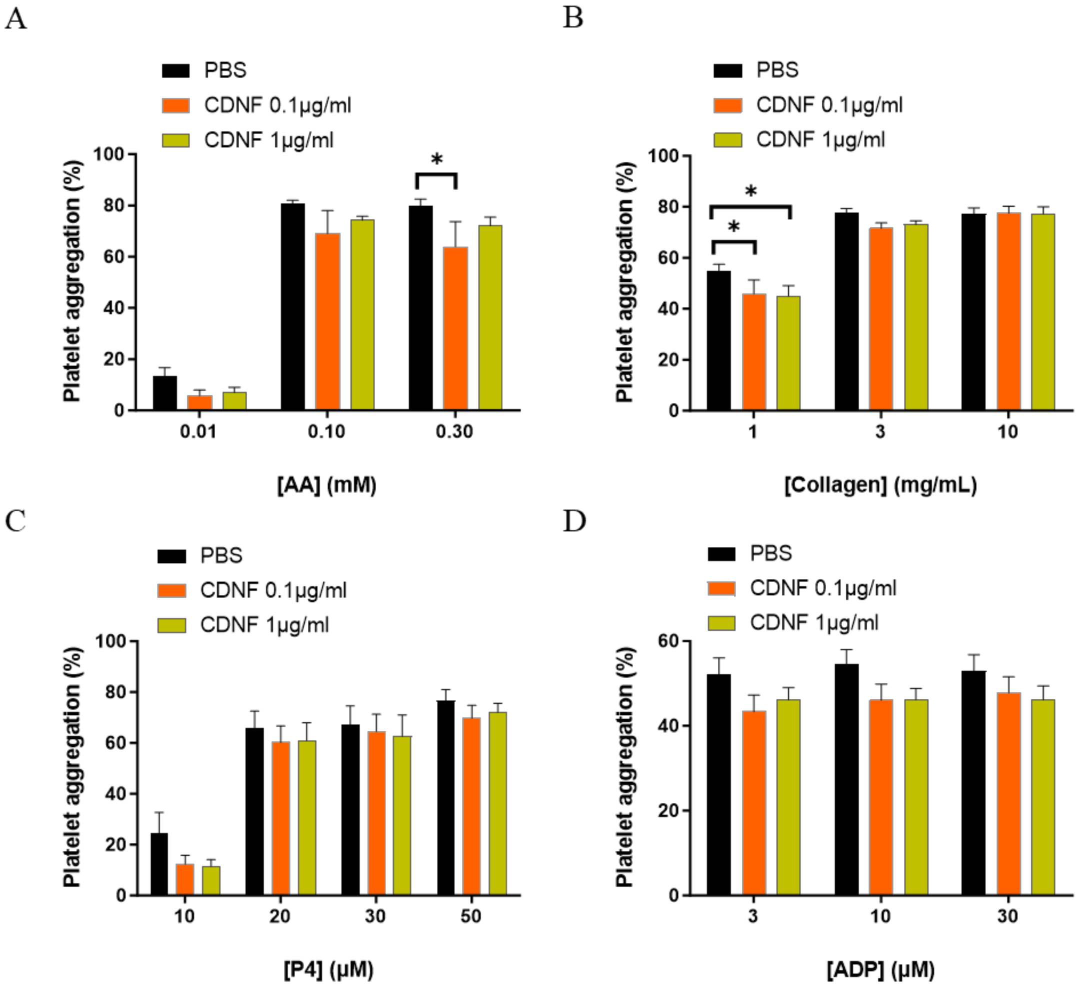

Fig. S3. **Extracellularly added CDNF suppressed the aggregation of rat washed platelets in the presence of AA or collagen.** To investigate the effects of CDNF on the aggregation responses of washed platelets obtained from naïve rats to various agonists. **(A-D)** The representative the maximum aggregation rates of WP stimulated with AA (A), collagen (B), P4 (C), ADP (D). *$p < 0.05$, **$p < 0.01$ indicate comparison with PBS with Bonferroni's post hoc test following two-way ANOVA. The results demonstrated that CDNF exhibited inhibitory effects on AA and collagen-induced washed platelet aggregation. However, no significant effect of CDNF was observed on P4 and ADP-induced platelet aggregation.

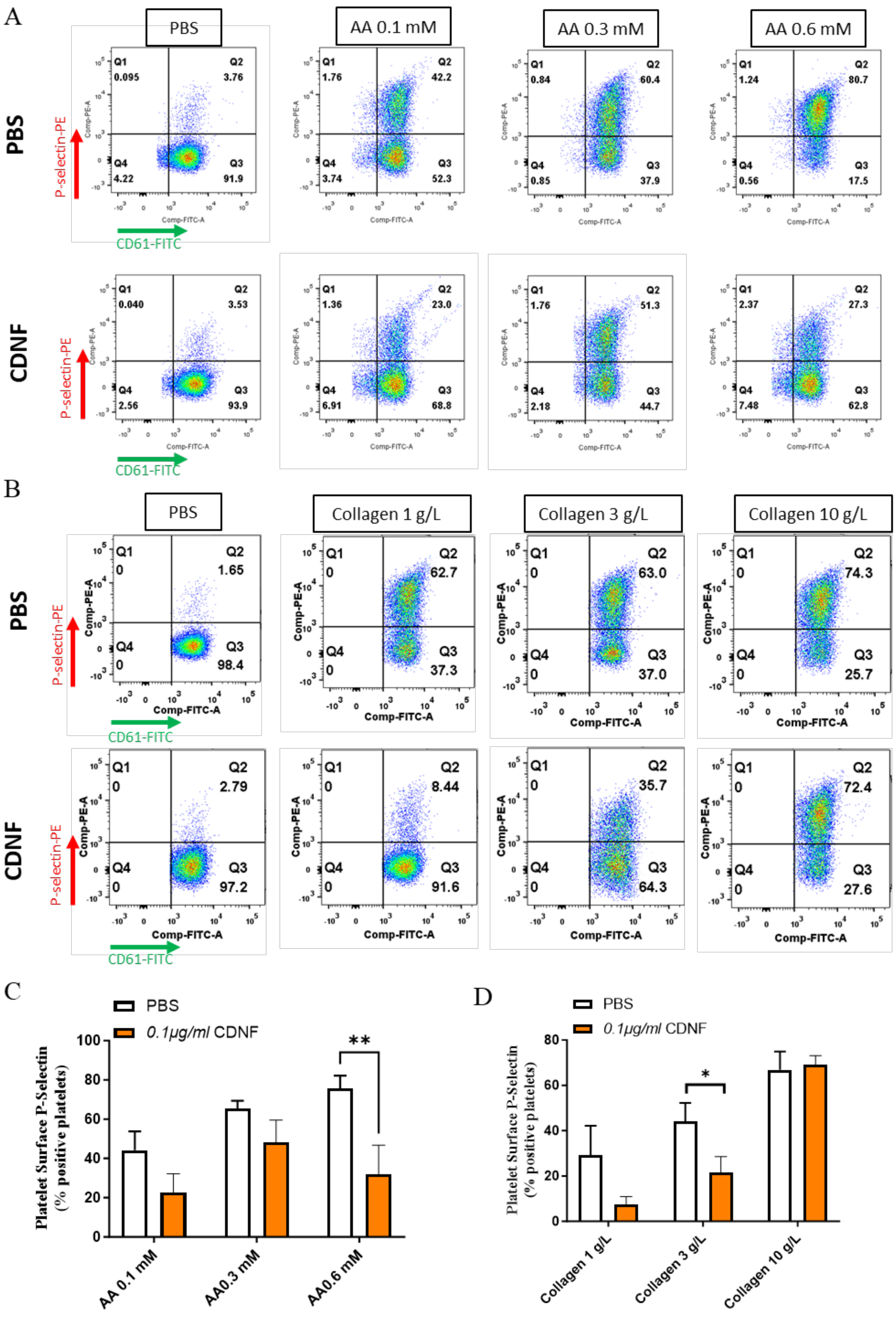
A
PBS
AA 0.1 mM
AA 0.3 mM
AA 0.6 mM
PBS
CDNF
P-selectin-PE
CD61-FITC
Comp-PE-A
Comp-FITC-A
Q1 0.095
Q2 3.76
Q4 4.22
Q3 91.9
Q1 1.76
Q2 42.2
Q4 3.74
Q3 52.3
Q1 0.84
Q2 60.4
Q4 0.85
Q3 37.9
Q1 1.24
Q2 80.7
Q4 0.56
Q3 17.5
Q1 0.040
Q2 3.53
Q4 2.56
Q3 93.9
Q1 1.36
Q2 23.0
Q4 6.91
Q3 68.8
Q1 1.76
Q2 51.3
Q4 2.18
Q3 44.7
Q1 2.37
Q2 27.3
Q4 7.48
Q3 62.8
B
PBS
Collagen 1 g/L
Collagen 3 g/L
Collagen 10 g/L
Q1 0
Q2 1.65
Q4 0
Q3 98.4
Q1 0
Q2 62.7
Q4 0
Q3 37.3
Q1 0
Q2 63.0
Q4 0
Q3 37.0
Q1 0
Q2 74.3
Q4 0
Q3 25.7
Q1 0
Q2 2.79
Q4 0
Q3 97.2
Q1 0
Q2 8.44
Q4 0
Q3 91.6
Q1 0
Q2 35.7
Q4 0
Q3 64.3
Q1 0
Q2 72.4
Q4 0
Q3 27.6
C
PBS
0.1μg/ml CDNF
Platelet Surface P-Selectin (% positive platelets)
AA 0.1 mM
AA0.3 mM
AA0.6 mM
**
D
PBS
0.1μg/ml CDNF
Platelet Surface P-Selectin (% positive platelets)
Collagen 1 g/L
Collagen 3 g/L
Collagen 10 g/L
*

Fig. S4. **Effects of CDNF on P-selectin expression of AA-, or collagen-treated washed platelets obtained from rats.** By Using flow cytometry to measure the expression of p-selectin/CD61^{+} in washed platelets and observe whether CDNF treatment affect the expression of p-selectin on platelets exposed to different concentrations of AA (A) and collagen (B). (C & D) The quantitative analysis reveals the ratios of the P-selection/CD61-positive platelets to CD61-positive platelets, indicating that CDNF treatment could suppress AA-induced or collagen-induced platelet activation in a dose-dependent manner. The mean ± SEM is represented by each vertical bar. (*p < 0.05, **p < 0.01, by Fisher's LSD test, following two-way ANOVA). In C and D Platelet surface not Platelet **S**urface.

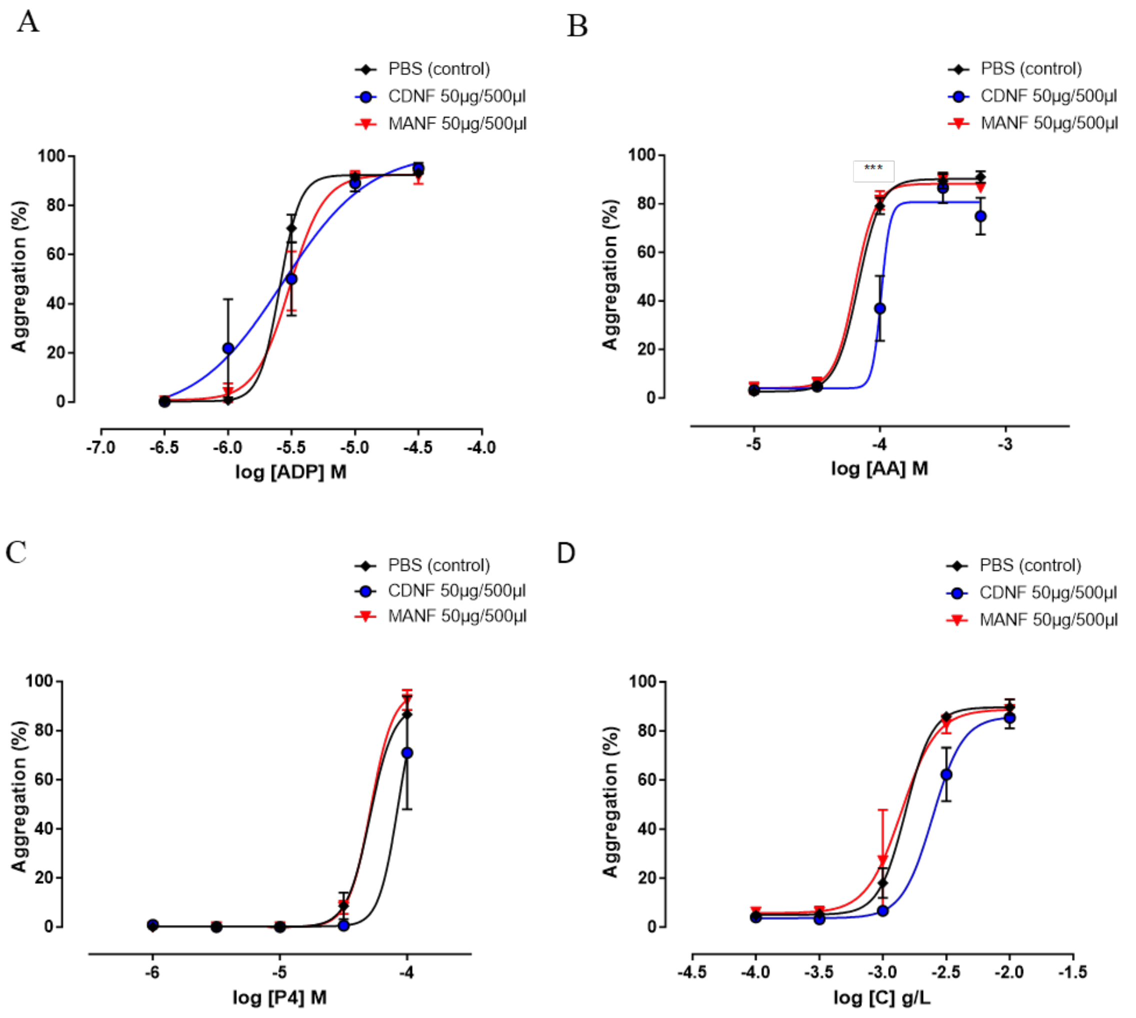

Fig. S5. The effect of subcutaneous injection of CDNF on PRP aggregation responses to different agonists. To explores the biological effects of CDNF or its homologous protein, rhMANF, on agonist-induced aggregation responses of rat PRP, the representative dose-response curve for PRP aggregation rates induced by various agonists. Each point represents the Mean ± SEM from separate 4-5 experiments. (A-D) ***$p < 0.001$ by Bonferroni's multiple comparisons test, following two-way ANOVA, compared with the control group. The findings indicate that only the subcutaneous injection of CDNF (50µg/500µl) suppresses the aggregation response of rat PRP stimulated by 0.1mM AA.

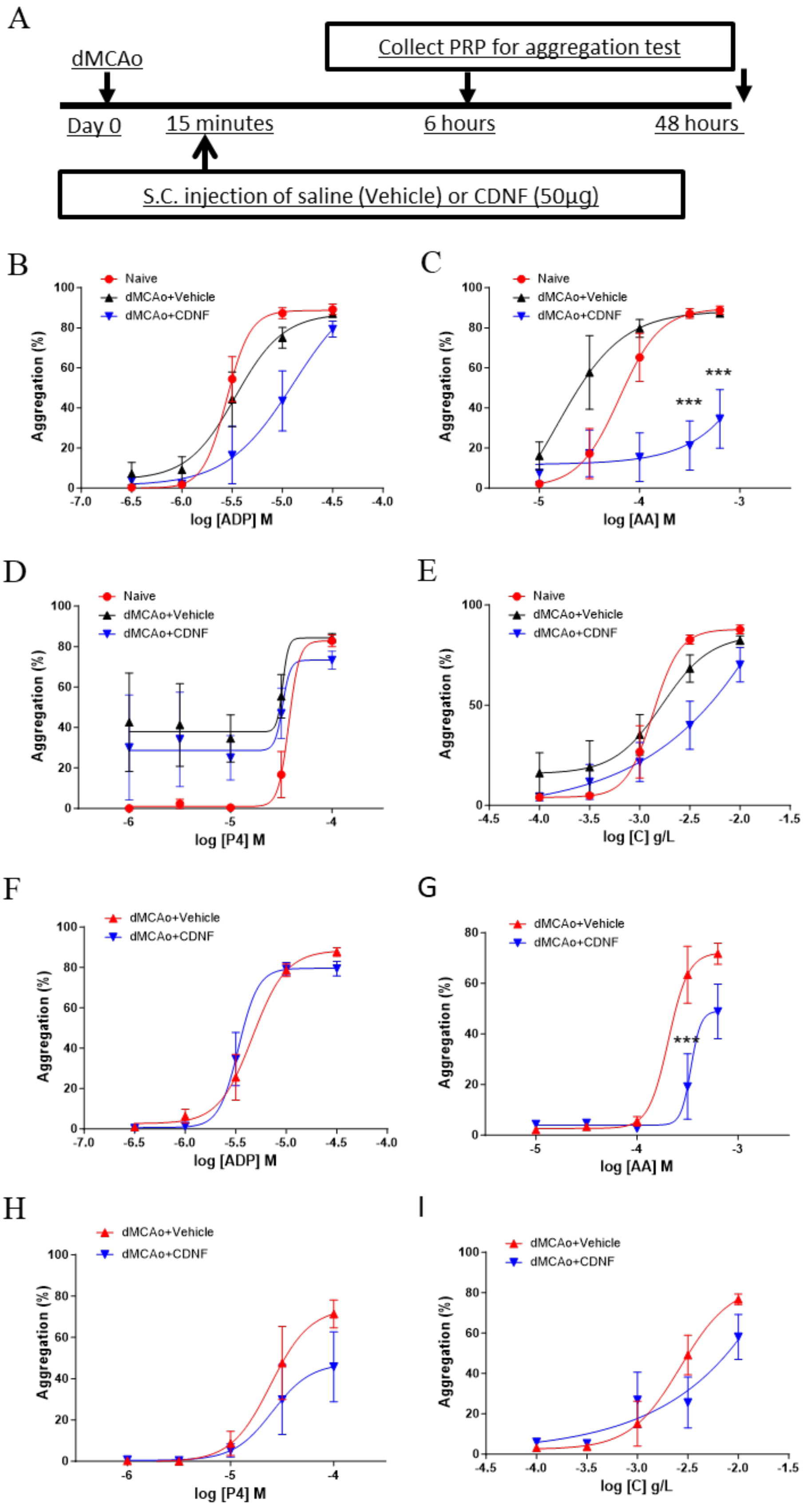
A
dMCAo
Collect PRP for aggregation test
Day 0
15 minutes
6 hours
48 hours
S.C. injection of saline (Vehicle) or CDNF (50μg)
B
Naive
dMCAo+Vehicle
dMCAo+CDNF
Aggregation (%)
log [ADP] M
C
Naive
dMCAo+Vehicle
dMCAo+CDNF
Aggregation (%)
log [AA] M
***
***
D
Naive
dMCAo+Vehicle
dMCAo+CDNF
Aggregation (%)
log [P4] M
E
Naive
dMCAo+Vehicle
dMCAo+CDNF
Aggregation (%)
log [C] g/L
F
dMCAo+Vehicle
dMCAo+CDNF
Aggregation (%)
log [ADP] M
G
dMCAo+Vehicle
dMCAo+CDNF
Aggregation (%)
log [AA] M
***
H
dMCAo+Vehicle
dMCAo+CDNF
Aggregation (%)
log [P4] M
I
dMCAo+Vehicle
dMCAo+CDNF
Aggregation (%)
log [C] g/L

Fig. S6. **Effects of systemic administration of CDNF in rats on aggregation responses of PRP stimulated by variable agonists. (A)** Timetable of experiment. The rats underwent dMCAo surgery, and they were divided into naïve, dMCAo+ vehicle (saline), and dMCAo+ CDNF randomly. At 15 minutes after reperfusion, the animals received a subcutaneous injection of either CDNF or saline once. PRP samples were collected at 6 and 48 hours post-dMCAo. Different concentrations of agonists (ADP, AA, P4 and collagen) was administered to PRP samples derived from rats at 6 hours (B-E) and 48 hours (F-I) after dMCAo. Then, the absorbance of PRP samples was detected by a multiplate spectrometer. ***$p<0.001$ by Bonferroni's multiple comparisons test, following two-way ANOVA, compared with the dMCAo+Vehicle group.

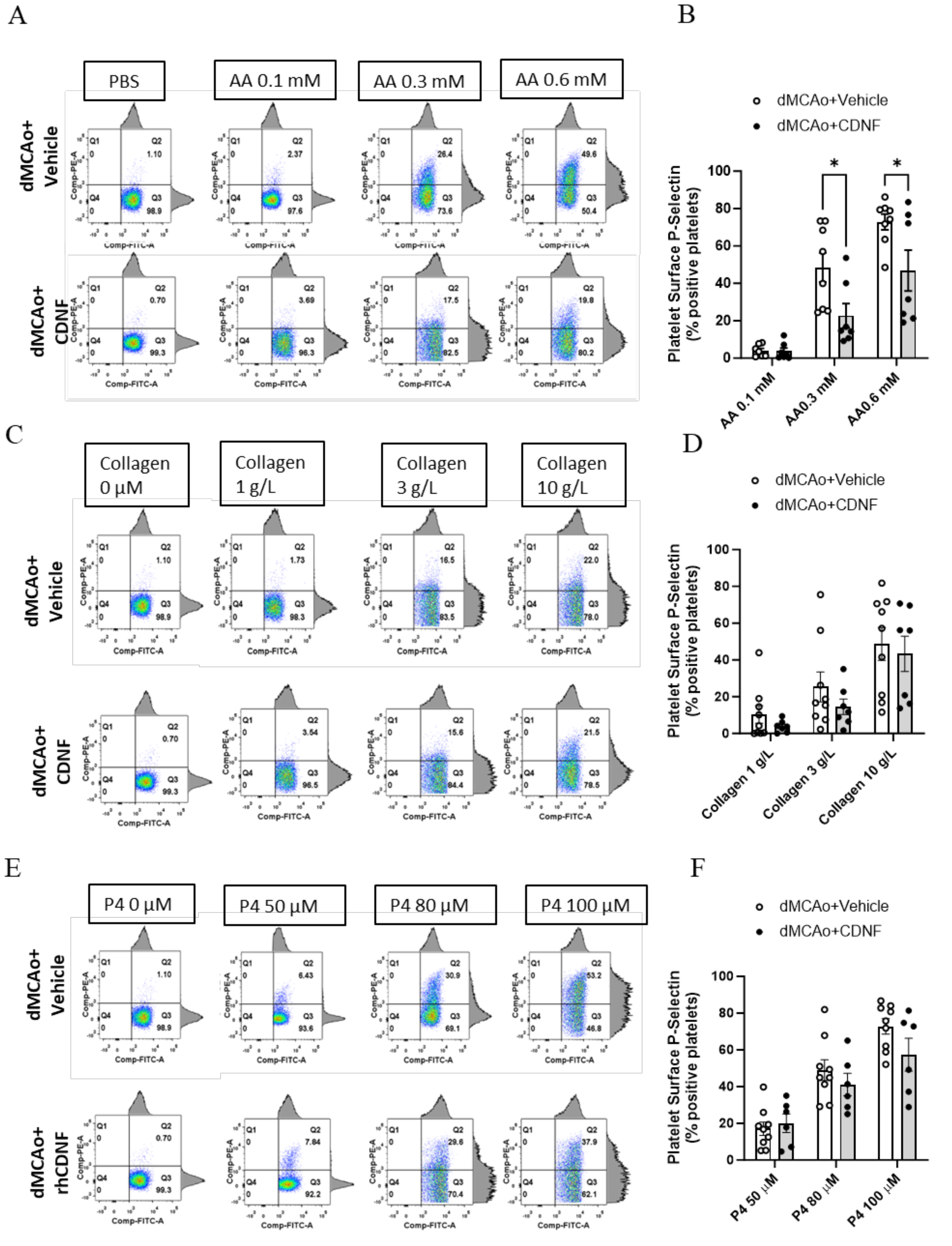

Fig. S7. **The systemic administration of CDNF reduced the AA-increased surface expression of P-selectin on platelets derived from stroked rats.** (A & B) The effect of

CDNF on P-selectin expression of AA-treated PRP derived from stroked rats on day 2 post-dMCAo. (C & D) The effect of CDNF on the collagen-induced expression of P-selectin on platelets from stroked rat on day 2 post-dMCAo. (E & F) The effects of CDNF on the P4-induced expression of P-selectin on platelets from stroked rats on day 2 after dMCAo. *$p<0.05$ by Bonferroni's multiple comparisons test, following two-way ANOVA, compared with the dMCAo+Vehicle group.

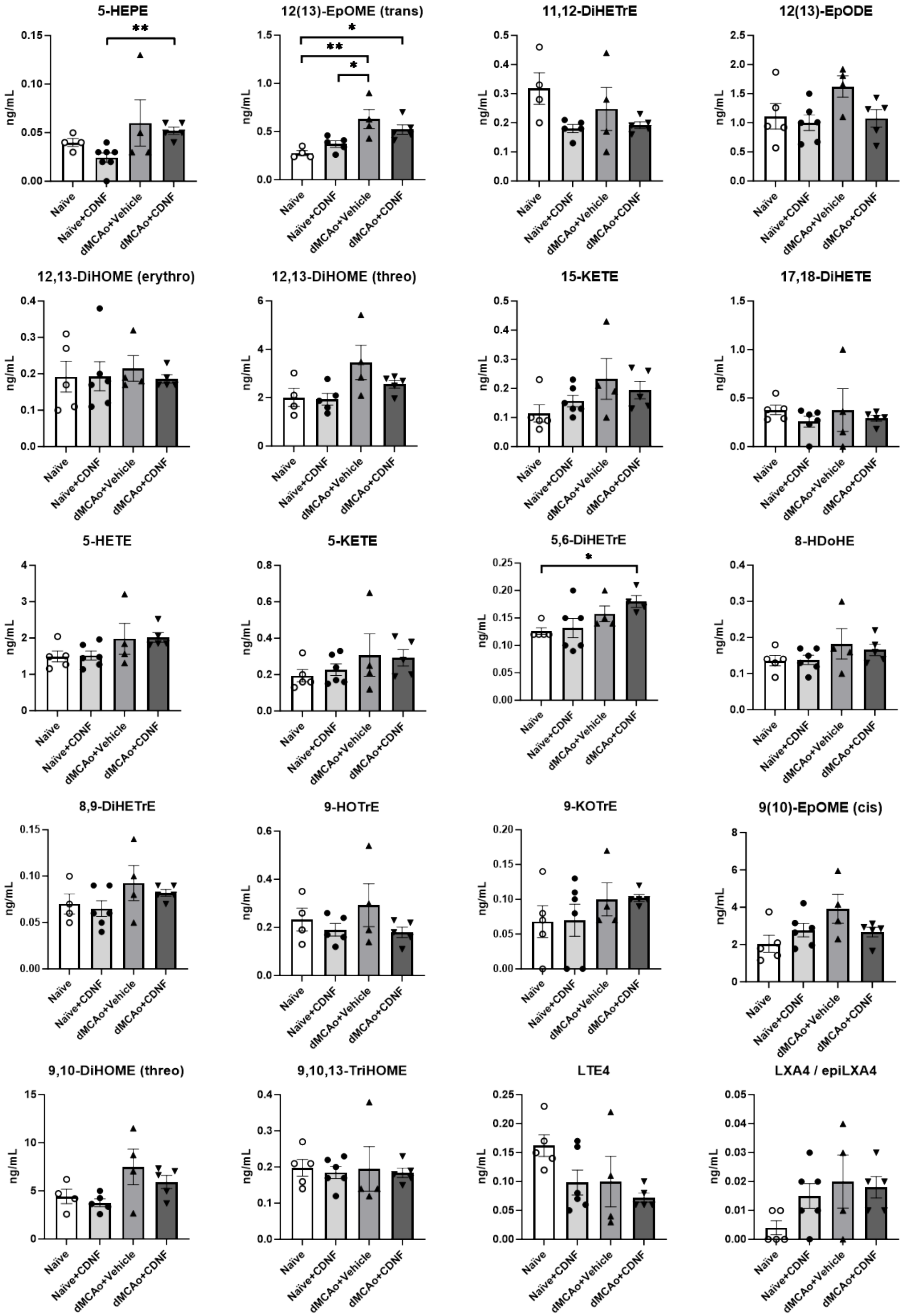
5-HEPE
12(13)-EpOME (trans)
11,12-DiHETrE
12(13)-EpODE
12,13-DiHOME (erythro)
12,13-DiHOME (threo)
15-KETE
17,18-DiHETE
5-HETE
5-KETE
5,6-DiHETrE
8-HDoHE
8,9-DiHETrE
9-HOTrE
9-KOTrE
9(10)-EpOME (cis)
9,10-DiHOME (threo)
9,10,13-TriHOME
LTE4
LXA4 / epiLXA4
ng/mL
Naïve
Naïve+CDNF
dMCAo+Vehicle
dMCAo+CDNF

Fig. S8. The levels of oxylipins in plasma detected 5h after ischemic stroke in a rat model. The rats were divided into four groups randomly (n=4-6 animals/group). Two control groups without stroke received a subcutaneous injection of either CDNF or saline (vehicle). Two stroke groups underwent dMCAo surgery with 60 min occlusion and four hours after reperfusion the animals received a subcutaneous injection of either CDNF or saline (vehicle). Rat plasma was collected 1 h after injection. 5-HEPE, (±)5-hydroxy-6E, 8Z,11Z,14Z,17Z-eicosapentaenoic acid; 12(13)-EpOME(trans), (±)12,13-epoxy-9Z-octadecenoic acid; 11,12-diHETrE, 11,12-dihydroxy-5Z,8Z,14Z-eicosatrienoic acid; 12(13)-EpODE, 12,13-epoxy-9Z,15Z-octadecadienoic acid; 12,13-DiHOME(erythro), (±)12,13-dihydroxy-9Z-octadecenoic acid; 12,13DiHOME(threo), (±)12,13-dihydroxy-9Z-octadecenoic acid; 15-KETE, 15-oxo-5Z,8Z,11Z,13E-eicosatetraenoic acid; 17,18-DiHETE, (±)17,18-dihydroxy-5Z,8Z,11Z,14Z-eicosatetraenoic acid; 5-HETE, (±)5-hydroxy-6E,8Z,11Z,14Z-eicosatetraenoic acid; 5-KETE, 5-oxo-6E,8Z,11Z,14Z-eicosatetraenoic acid; 5,6-DiHETrE, (±)5,6-dihydroxy-8Z,11Z,14Z-eicosatrienoic acid; 8-HDoHE, (±)8-hydroxy-4Z,6E,10Z,13Z,16Z,19Z-docosahexaenoic acid; 8,9-DiHETrE, (±)8,9-dihydroxy-5Z,11Z,14Z-eicosatrienoic acid; 9-HOTrE, 9S-hydroxy-10E,12Z,15Z-octadecatrienoic acid; 9-KOTrE, 9-oxo-10E,12Z,15Z-octadecatrienoic acid; 9(10)-EpOME(cis), (±)9,10-epoxy-12Z-octadecenoic acid; 9,10-DiHOME(threo), (±)9,10-dihydroxy-12Z-octadecenoic acid; 9,10,13-TriHOME, 9S,10S,13S-trihydroxy-11E-octadecenoic acid; LTE_4, 5S-hydroxy-6R-(S-cysteinyl)-7E,9E,11Z,14Z-eicosatetraenoic acid; LXA_4/EpiLXA_4, 5S,6R,15S-trihydroxy-7E,9E,11Z,13E-eicosatetraenoic acid. * $p<0.05$, ** $p < 0.01$ by Tukey's multiple comparisons test, following one-way ANOVA. Mean ± SEM is shown.

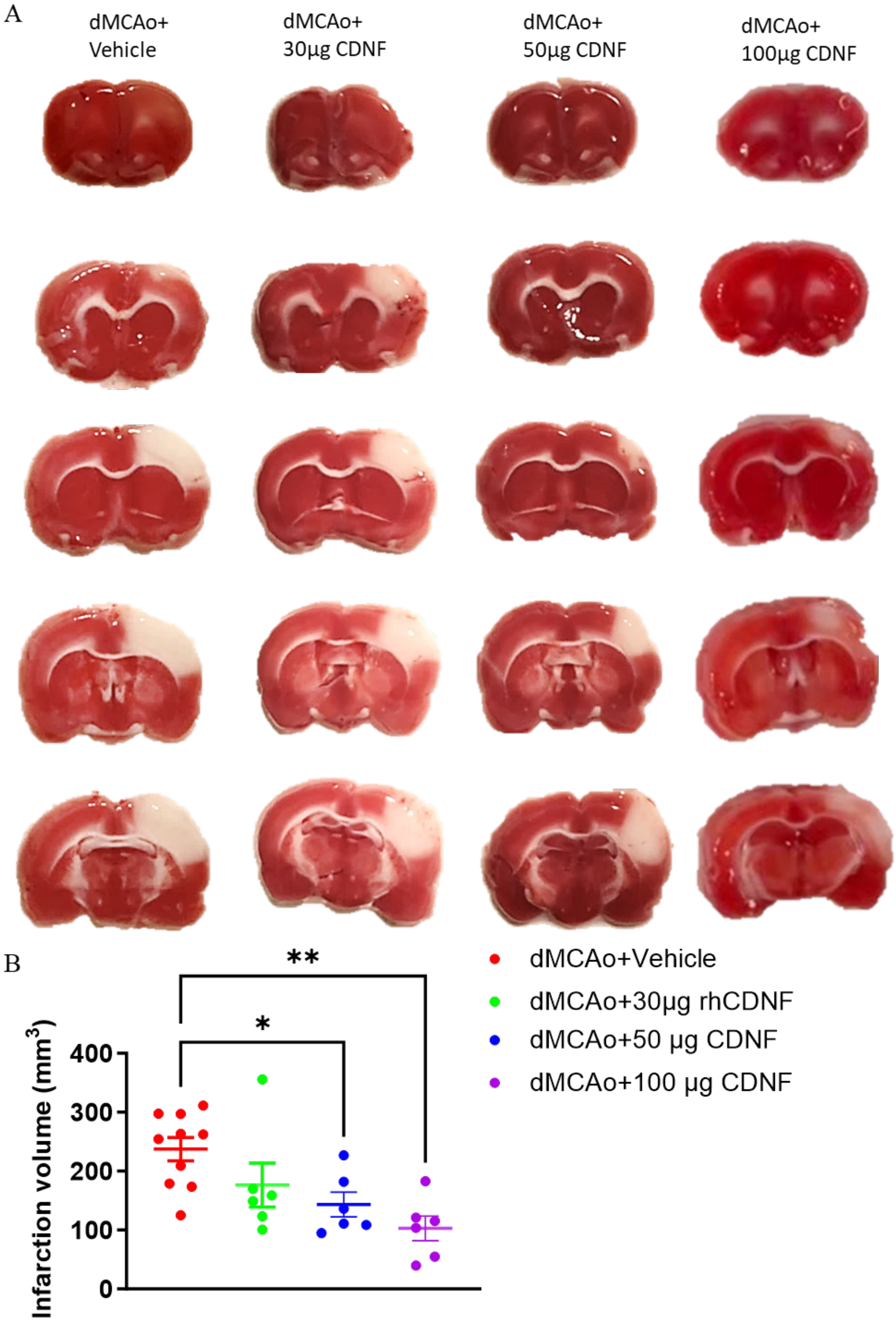
A
dMCAo+ Vehicle
dMCAo+ 30μg CDNF
dMCAo+ 50μg CDNF
dMCAo+ 100μg CDNF
B
**
*
Infarction volume (mm³)
400
300
200
100
0
dMCAo+Vehicle
dMCAo+30μg rhCDNF
dMCAo+50 μg CDNF
dMCAo+100 μg CDNF

Fig. S9. Effects of systemic administration of CDNF on infarction volume on day2 post-dMCAo. A & B). Rats were injected with saline or CDNF (30, 50, and 100 μg, s.c.) at 15 minutes after reperfusion. (A) Photographs of representative brain sections showing the sizes of infarction area on days 2 after ischemia/reperfusion injury. (B) Infarction volume on days 2 post-dMCAo was determined by TTC-stained morphometric measurement. *p <0.05, **p <0.01 vs. dMCAo + vehicle group by one-way ANOVA followed by Dunnett corrections. Mean ± SEM is shown.

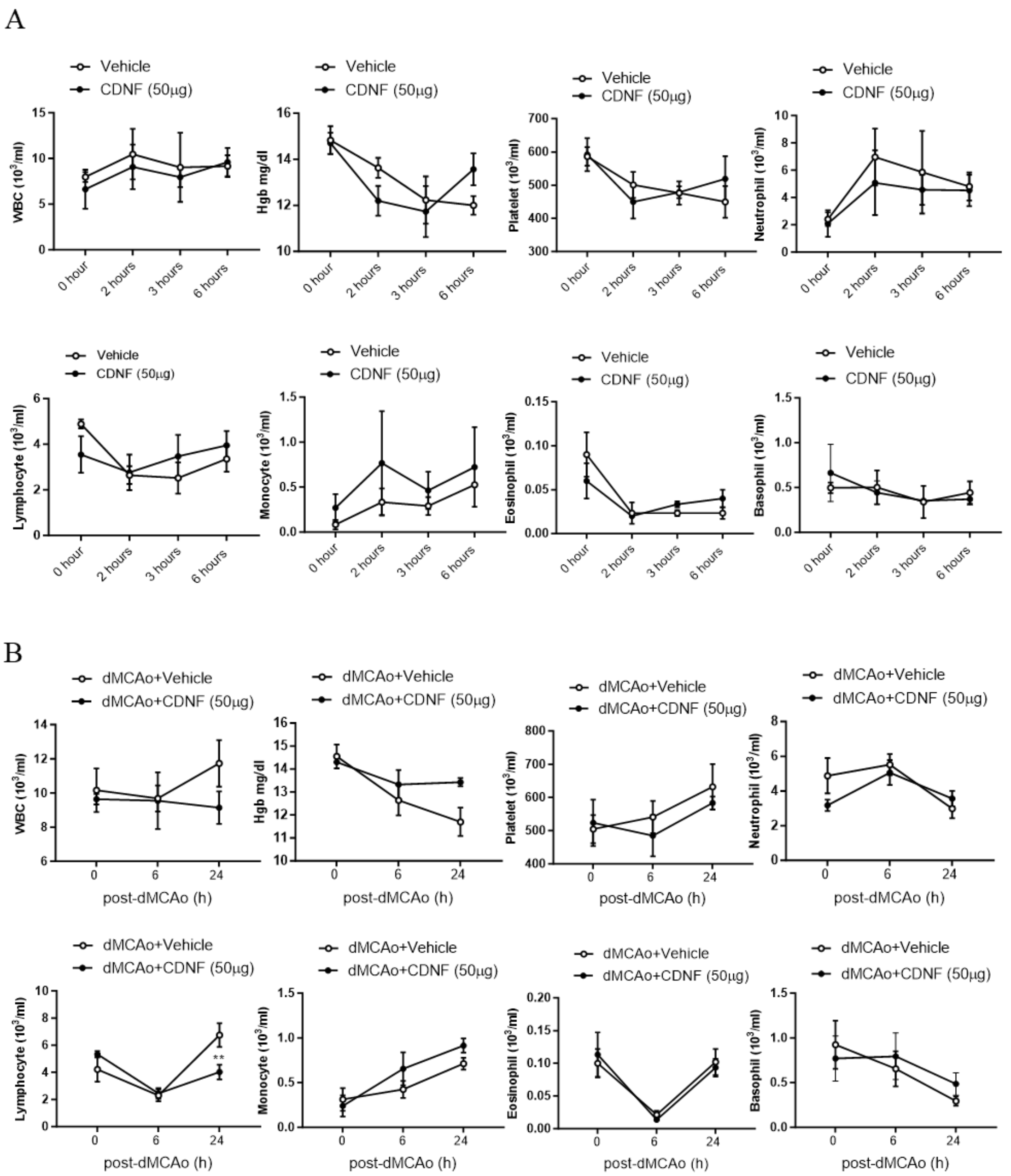

Fig. S10. **The temporal profile of blood parameters after the subcutaneous injection of CDNF in rats with or without dMCAo surgery. (A)** There is no significant difference in the hematologic parameters at different time points between vehicle group and CDNF treatment group. (B) While the number of lymphocytes is decreased on day 1 post-stroke in CDNF-treated group, there is no significant difference in other hematological parameters

between two groups at 6 hours or 24 hours post-dMCAo. **$p < 0.01$ signifies that a comparison with PBS was conducted using Bonferroni's post hoc test, following a two-way ANOVA.

Supplemental Tables

Table S1. Peptides of interest are fragmented repeatedly during their elution times and extracted ion chromatograms of selected fragment ions are then used for protein quantification.

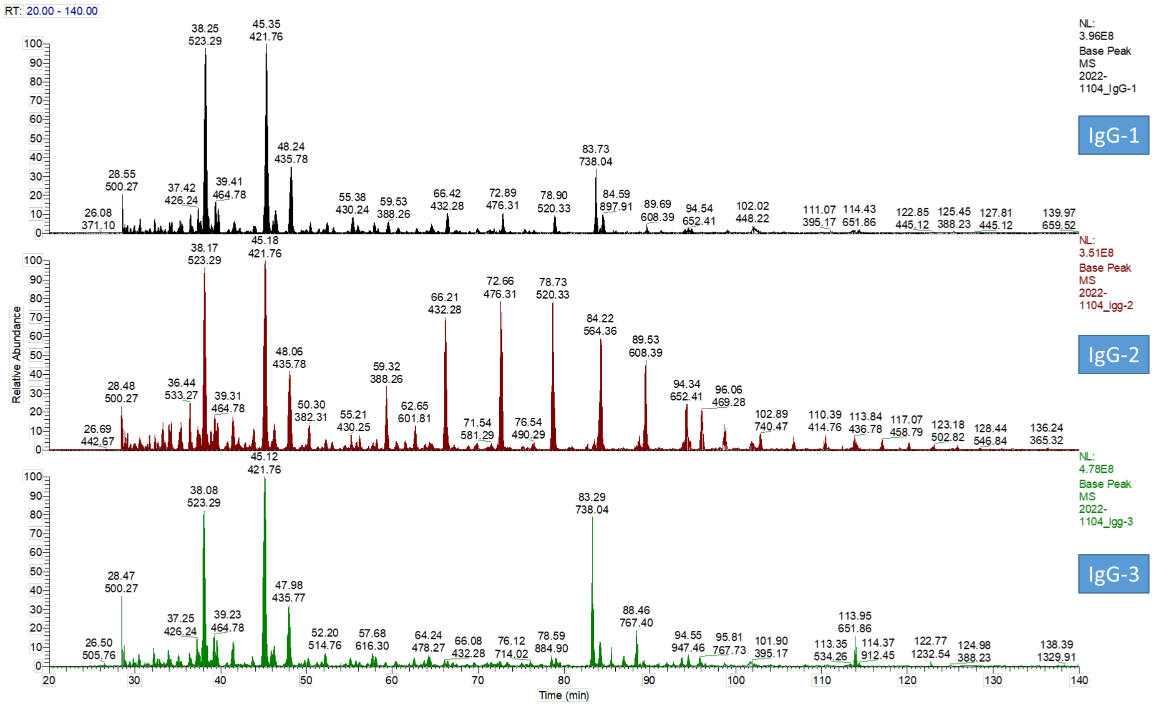

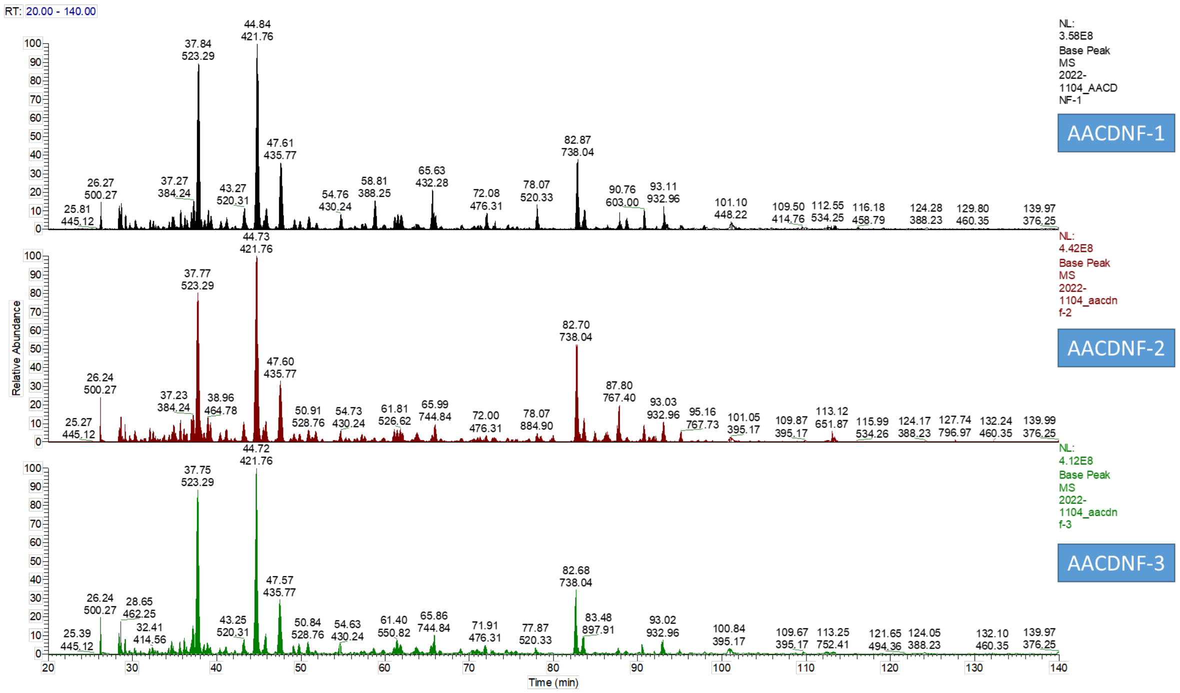

Accession	Description	Gene name	(ACCDNF_mix) / (IgG_mix) Normalized ratio	Trend	Variability [%]	t-test	Coverage [%]	# Peptides	# PSMs	# Unique Peptides	# Protein Groups	# AAs
Q86SG5	Protein S100-A7A OS=Homo sapiens OX=9606 GN=S1	S100A7A	0.004	-			54	7	53	2	1	101
Q14210	Lymphocyte antigen 6D OS=Homo sapiens OX=9606 G	LY6D	0.004	-			15	2	2	2	1	128
P04279	Semenogelin-1 OS=Homo sapiens OX=9606 GN=SEM	SEMG1	0.006	-	0		10	4	5	4	1	462
Q9NZH8	Interleukin-36 gamma OS=Homo sapiens OX=9606 GN	IL36G	0.011	-	0.02		22	3	5	3	1	169
P02452	Collagen alpha-1(I) chain OS=Homo sapiens OX=9606	COL1A1	0.012	-	0.05		4	4	12	4	1	1464
P31151	Protein S100-A7 OS=Homo sapiens OX=9606 GN=S10	S100A7	0.027	-	0.3		55	8	73	3	1	101
P29508	Serpin B3 OS=Homo sapiens OX=9606 GN=SERPINB3	SERPINB3	0.028	-	0.21		24	10	32	10	1	390
Q8WWY7	WAP four-disulfide core domain protein 12 OS=Homo s	WFDC12	0.033	-	0.35		32	3	7	3	1	111
Q01469	Fatty acid-binding protein 5 OS=Homo sapiens OX=960	FABP5	0.037	-	0.48		67	9	22	9	1	135
P08123	Collagen alpha-2(I) chain OS=Homo sapiens OX=9606	COL1A2	0.037	-	0.1		1	2	5	2	1	1366
P05089	Arginase-1 OS=Homo sapiens OX=9606 GN=ARG1 PE	ARG1	0.040	-	0.63		6	3	3	3	1	322
P12273	Prolactin-inducible protein OS=Homo sapiens OX=9606	PIP	0.040	-	0.36		18	2	5	2	1	146
Q02383	Semenogelin-2 OS=Homo sapiens OX=9606 GN=SEM	SEMG2	0.040	-	0.16		12	4	5	4	1	582
P31944	Caspase-14 OS=Homo sapiens OX=9606 GN=CASP1	CASP14	0.048	-	0.58		24	7	22	7	1	242
O75223	Gamma-glutamylcyclotransferase OS=Homo sapiens OX	GGCT	0.064	-	1.12		12	2	4	2	1	188
P25311	Zinc-alpha-2-glycoprotein OS=Homo sapiens OX=9606	AZGP1	0.070	-	2.4		22	5	10	5	1	298
P32119	Peroxiredoxin-2 OS=Homo sapiens OX=9606 GN=PRD	PRDX2	0.070	-	2.18		11	2	2	2	1	198
P04040	Catalase OS=Homo sapiens OX=9606 GN=CAT PE=1	CAT	0.070	-	1.69		9	4	5	4	1	527
Q8N1N4	Keratin, type II cytoskeletal 78 OS=Homo sapiens OX=9	KRT78	0.075	-	2.58		10	6	15	3	1	520
P01040	Cystatin-A OS=Homo sapiens OX=9606 GN=CSTA PE	CSTA	0.085	-	2.04		74	6	51	6	1	98
Q9NZT1	Calmodulin-like protein 5 OS=Homo sapiens OX=9606	CALML5	0.085	-	1.7		32	5	39	5	1	146
O76015	Keratin, type I cuticular Ha8 OS=Homo sapiens OX=960	KRT38	0.089	-	3.65		2	2	19	1	1	456
P13647	Keratin, type II cytoskeletal 5 OS=Homo sapiens OX=96	KRT5	0.092	-	4.56		45	31	175	17	1	590
P02751	Fibronectin OS=Homo sapiens OX=9606 GN=FN1 PE=	FN1	0.093	-	3.27		26	53	287	53	1	2477
Q6UWP8	Suprabasin OS=Homo sapiens OX=9606 GN=SBSN P	SBSN	0.093	-	3.67		27	7	11	7	1	590
P01861	Immunoglobulin heavy constant gamma 4 OS=Homo sap	IGHG4	0.094	-	0		27	6	71	1	1	327
[illegible]	[illegible]	[illegible]	[illegible]	[illegible]	[illegible]	[illegible]	[illegible]	[illegible]	[illegible]	[illegible]	[illegible]	[illegible]

Table S2. IPA analysis based on differentially expressed proteins in AA- and CDNF-treated platelets.

© 2000-2023 QIAGEN. All rights reserved.

Ingenuity Canonical Pathways	-log(p-value)	Ratio	z-score	Molecules
Actin Cytoskeleton Signaling	13.7	0.0583	2.111	ACTA1,ACTB,ACTG1,CFL1,FLNA,GSN,MSN,MYH9,MYL12B,PDGFA,PDGFB,RAP1B,TLN1,VCL
Integrin Signaling	11.8	0.0585	2.714	ACTA1,ACTB,ACTG1,GSN,ILK,LIMS1,MYL12B,PDGFB,RAP1B,TLN1,VASP,VCL
ILK Signaling	10.6	0.0561	1.667	ACTA1,ACTB,ACTG1,CFL1,FLNA,ILK,LIMS1,MYH9,VCL,VEGFC,VIM
Germ Cell-Sertoli Cell Junction Signaling	9.97	0.0602	#NUM!	ACTA1,ACTB,ACTG1,CFL1,GSN,ILK,RAP1B,TGFB1,TUBB1,VCL
Pulmonary Fibrosis Idiopathic Signaling Pathway	8.32	0.0342	2.646	ACTA1,ACTB,ACTG1,CCN2,ILK,PDGFA,PDGFB,PLG,RAP1B,TGFB1,VIM
Agranulocyte Adhesion and Diapedesis	6.79	0.0415	#NUM!	ACTA1,ACTB,ACTG1,CCL5,MSN,MYH9,PF4,PPBP
HIF1α Signaling	6.61	0.0392	2.121	HK1,HSPA5,PDGFB,PKM,RAP1B,TGFB1,VEGFC,VIM
Protein Kinase A Signaling	6.39	0.0254	2.121	FLNA,H1-2,H1-3,H1-5,H3-3A/H3-3B,MYL12B,PDIA3,RAP1B,TGFB1,VASP
VEGF Signaling	6.22	0.0619	1.633	ACTA1,ACTB,ACTG1,RAP1B,VCL,VEGFC
Wound Healing Signaling Pathway	6.1	0.0336	2.828	CCL5,PDGFA,PDGFB,PF4,RAP1B,TGFB1,VEGFC,VIM
Paxillin Signaling	5.99	0.0566	2.449	ACTA1,ACTB,ACTG1,RAP1B,TLN1,VCL
Tight Junction Signaling	5.84	0.0393	#NUM!	ACTA1,ACTB,ACTG1,MYH9,TGFB1,VASP,VCL
Virus Entry via Endocytic Pathways	5.74	0.0513	#NUM!	ACTA1,ACTB,ACTG1,B2M,FLNA,RAP1B
Remodeling of Epithelial Adherens Junctions	5.69	0.0758	#NUM!	ACTA1,ACTB,ACTG1,TUBB1,VCL
Leukocyte Extravasation Signaling	5.67	0.037	1.633	ACTA1,ACTB,ACTG1,MSN,RAP1B,VASP,VCL
Hepatic Fibrosis / Hepatic Stellate Cell Activation	5.65	0.0368	#NUM!	CCL5,CCN2,MYH9,PDGFA,PDGFB,TGFB1,VEGFC
RHOA Signaling	5.63	0.0492	2.449	ACTA1,ACTB,ACTG1,CFL1,MSN,MYL12B
Sertoli Cell-Sertoli Cell Junction Signaling	5.48	0.0347	#NUM!	ACTA1,ACTB,ACTG1,ILK,RAP1B,TUBB1,VCL
Caveolar-mediated Endocytosis Signaling	5.41	0.0667	#NUM!	ACTA1,ACTB,ACTG1,B2M,FLNA
RHOGDI Signaling	5.26	0.0321	-1.633	ACTA1,ACTB,ACTG1,CFL1,MSN,MYH9,MYL12B
Cellular Effects of Sildenafil (Viagra)	5.23	0.042	#NUM!	ACTA1,ACTB,ACTG1,MYH9,MYL12B,PDIA3
Dilated Cardiomyopathy Signaling Pathway	5.11	0.04	#NUM!	ACTA1,ACTB,ACTG1,ILK,MYH9,TPM1
Fcγ Receptor-mediated Phagocytosis in Macropha	4.95	0.0538	2.236	ACTA1,ACTB,ACTG1,TLN1,VASP
Signaling by Rho Family GTPases	4.71	0.0264	2.449	ACTA1,ACTB,ACTG1,CFL1,MSN,MYL12B,VIM
Axonal Guidance Signaling	4.65	0.0183	#NUM!	CFL1,MYL12B,PDGFA,PDGFB,PDIA3,RAP1B,TUBB1,VASP,VEGFC
Regulation of Actin-based Motility by Rho	4.6	0.0455	#NUM!	ACTA1,ACTB,CFL1,GSN,MYL12B
Gap Junction Signaling	4.48	0.0309	#NUM!	ACTA1,ACTB,ACTG1,PDIA3,RAP1B,TUBB1
Neutrophil Extracellular Trap Signaling Pathway	4.48	0.0202	2.828	ATP5F1A,CCL5,GP1BA,H3-3A/H3-3B,H4C1,HMGB1,PDIA3,PF4
PAK Signaling	4.47	0.0427	2	CFL1,MYL12B,PDGFA,PDGFB,RAP1B
Hepatic Fibrosis Signaling Pathway	4.35	0.0194	2.828	CCL5,CCN2,MYL12B,PDGFA,PDGFB,RAP1B,TGFB1,VEGFC
Clathrin-mediated Endocytosis Signaling	4.33	0.0291	#NUM!	ACTA1,ACTB,ACTG1,PDGFA,PDGFB,VEGFC
Glucocorticoid Receptor Signaling	4.32	0.0166	#NUM!	ACTB,ATP5F1A,B2M,CCL5,H3-3A/H3-3B,HMGB1,HSPA5,RAP1B,TGFB1
Calcium Signaling	4.27	0.0284	#NUM!	ACTA1,MYH9,RAP1B,TPM1,TPM2,TPM4
Role of Macrophages, Fibroblasts and Endothelial	4.2	0.022	#NUM!	CCL5,PDGFA,PDGFB,PDIA3,RAP1B,TGFB1,VEGFC
Agrin Interactions at Neuromuscular Junction	4.2	0.0588	2	ACTA1,ACTB,ACTG1,RAP1B
NAD Signaling Pathway	4.03	0.0345	2.236	H1-2,H1-3,H1-5,PDGFB,TGFB1
S100 Family Signaling Pathway	3.96	0.0134	0.333	ATP5F1A,ILK,MYH9,PDIA3,PLG,TGFB1,TPM1,TPM2,TPM4,VEGFC
Crosstalk between Dendritic Cells and Natural Kille	3.71	0.044	#NUM!	ACTA1,ACTB,ACTG1,TLN1
Tumor Microenvironment Pathway	3.61	0.0281	2.236	PDGFA,PDGFB,RAP1B,TGFB1,VEGFC
Mechanisms of Viral Exit from Host Cells	3.54	0.0732	#NUM!	ACTA1,ACTB,ACTG1
Regulation Of The Epithelial Mesenchymal Transit	3.46	0.026	2.236	PDGFA,PDGFB,RAP1B,TGFB1,VIM
ID1 Signaling Pathway	3.38	0.025	2.236	CCN2,RAP1B,TGFB1,VEGFC,VIM
Ephrin Receptor Signaling	3.37	0.0249	#NUM!	CFL1,PDGFA,PDGFB,RAP1B,VEGFC
Role of Tissue Factor in Cancer	3.32	0.0348	#NUM!	CCN2,CFL1,RAP1B,VEGFC
14-3-3-mediated Signaling	3.17	0.0317	#NUM!	PDIA3,RAP1B,TUBB1,VIM
Role of PKR in Interferon Induction and Antiviral R	3.1	0.0303	1	HMGB1,HSPA5,PDGFA,PDGFB
MSP-RON Signaling Pathway	3.09	0.0517	#NUM!	ACTA1,ACTB,ACTG1
MSP-RON Signaling In Cancer Cells Pathway	3.01	0.0288	2	FLNA,RAP1B,VEGFC,VIM
Granzyme A Signaling	2.84	0.0423	#NUM!	H1-2,H1-3,H1-5
Epithelial Adherens Junction Signaling	2.83	0.0256	1	CFL1,MYL12B,RAP1B,VCL
Estrogen Receptor Signaling	2.79	0.015	1.633	ATP5F1A,CFL1,MYL12B,PDIA3,RAP1B,VEGFC
Macropinocytosis Signaling	2.75	0.0395	#NUM!	PDGFA,PDGFB,RAP1B
Chemokine Signaling	2.74	0.039	#NUM!	CCL5,CFL1,RAP1B
Renal Cell Carcinoma Signaling	2.72	0.0385	#NUM!	PDGFB,RAP1B,TGFB1
Glioblastoma Multiforme Signaling	2.71	0.0238	2	PDGFA,PDGFB,PDIA3,RAP1B
Sirtuin Signaling Pathway	2.69	0.0175	2	ATP5F1A,H1-2,H1-3,H1-5,H3-3A/H3-3B
Granulocyte Adhesion and Diapedesis	2.66	0.023	#NUM!	CCL5,MSN,PF4,PPBP
Immunogenic Cell Death Signaling Pathway	2.63	0.0357	#NUM!	HMGB1,HSPA5,PDIA3
Apelin Cardiac Fibroblast Signaling Pathway	2.63	0.087	#NUM!	CCN2,TGFB1
PDGF Signaling	2.58	0.0345	#NUM!	PDGFA,PDGFB,RAP1B
Regulation of Cellular Mechanics by Calpain Prote	2.57	0.0341	#NUM!	RAP1B,TLN1,VCL
Natural Killer Cell Signaling	2.49	0.0207	0	B2M,CFL1,HSPA5,RAP1B
Macrophage Alternative Activation Signaling Pathw	2.49	0.0207	1	H3-3A/H3-3B,H4C1,PF4,TGFB1
Death Receptor Signaling	2.49	0.0319	#NUM!	ACTA1,ACTB,ACTG1
Pulmonary Healing Signaling Pathway	2.46	0.0203	1	CFL1,RAP1B,TGFB1,VEGFC
Human Embryonic Stem Cell Pluripotency	2.46	0.0203	2	PDGFA,PDGFB,RAP1B,TGFB1
Apelin Cardiomyocyte Signaling Pathway	2.44	0.0306	#NUM!	MYL12B,PDIA3,TGFB1
IL-8 Signaling	2.39	0.0194	#NUM!	MYL12B,RAP1B,VASP,VEGFC
PPAR Signaling	2.36	0.0288	#NUM!	PDGFA,PDGFB,RAP1B
Pathogen Induced Cytokine Storm Signaling Pathw	2.36	0.0147	2.236	CCL5,PF4,PPBP,TGFB1,VEGFC
NRF2-mediated Oxidative Stress Response	2.36	0.019	#NUM!	ACTA1,ACTB,ACTG1,RAP1B
EIF2 Signaling	2.35	0.0189	#NUM!	ACTA1,ACTB,HSPA5,RAP1B
Coagulation System	2.27	0.0571	#NUM!	F5,PLG
Sphingosine-1-phosphate Signaling	2.22	0.0256	#NUM!	PDGFA,PDGFB,PDIA3
Antigen Presentation Pathway	2.2	0.0526	#NUM!	B2M,PDIA3
Glioma Signaling	2.17	0.0246	#NUM!	PDGFA,PDGFB,RAP1B
Atherosclerosis Signaling	2.11	0.0234	#NUM!	PDGFA,PDGFB,TGFB1
Iron homeostasis signaling pathway	2.08	0.0227	#NUM!	HBA1/HBA2,PDGFA,PDGFB
Cardiac Hypertrophy Signaling	2.07	0.0157	2	MYL12B,PDIA3,RAP1B,TGFB1
STAT3 Pathway	2.05	0.0222	#NUM!	PDGFB,RAP1B,TGFB1
PFKFB4 Signaling Pathway	2	0.0417	#NUM!	HK1,TGFB1
Transcriptional Regulatory Network in Embryonic S	1.9	0.037	#NUM!	H3-3A/H3-3B,H4C1
CSDE1 Signaling Pathway	1.89	0.0364	#NUM!	TGFB1,VIM
HMGB1 Signaling	1.86	0.0189	#NUM!	HMGB1,RAP1B,TGFB1
HOTAIR Regulatory Pathway	1.86	0.0189	#NUM!	H3-3A/H3-3B,TGFB1,VIM
Endometrial Cancer Signaling	1.81	0.0333	#NUM!	ILK,RAP1B
Glutathione Redox Reactions II	1.81	0.2	#NUM!	PDIA3
Erythropoietin Signaling Pathway	1.8	0.0179	#NUM!	HBA1/HBA2,RAP1B,TGFB1
Neuroinflammation Signaling Pathway	1.8	0.013	2	B2M,CCL5,HMGB1,TGFB1
Myelination Signaling Pathway	1.74	0.0125	2	ILK,PDGFA,PDGFB,RAP1B
Trehalose Degradation II (Trehalase)	1.73	0.167	#NUM!	HK1
IL-17 Signaling	1.72	0.0168	#NUM!	RAP1B,TGFB1,VEGFC
Macrophage Classical Activation Signaling Pathwa	1.72	0.0167	#NUM!	CCL5,PKM,TGFB1
MicroRNA Biogenesis Signaling Pathway	1.7	0.0164	#NUM!	PDGFB,RAP1B,TGFB1
PPARα/RXRα Activation	1.7	0.0164	#NUM!	PDIA3,RAP1B,TGFB1
Glioma Invasiveness Signaling	1.68	0.0282	#NUM!	PLG,RAP1B
Ephrin B Signaling	1.67	0.0278	#NUM!	CAP1,CFL1
Adrenomedullin signaling pathway	1.64	0.0155	#NUM!	CFH,PDIA3,RAP1B
VDR/RXR Activation	1.61	0.026	#NUM!	CCL5,PDGFA
Role of JAK family kinases in IL-6-type Cytokine Si	1.61	0.026	#NUM!	TGFB1,VEGFC
PI3K/AKT Signaling	1.61	0.0152	#NUM!	ILK,LIMS1,RAP1B
Estrogen-Dependent Breast Cancer Signaling	1.58	0.025	#NUM!	HSD17B4,RAP1B
Role of MAPK Signaling in the Pathogenesis of Inf	1.57	0.0247	#NUM!	CCL5,RAP1B

Table S3. Profiling of targeted oxylipins through comprehensive LC-MS/MS analysis.

Reported

Compound Name	Standard	Systematic Name	Supplier	Cat Number	Rt (min)	SRM transition	IS
11,12-DiHETrE	(±)11,12-DiHETrE	11,12-dihydroxy-5Z,8Z,14Z-eicosatrienoic acid	Cayman Chemical	10007266	7,6	337.3 > 166.9	d11-11,12-DiHETrE
11-HEPE	(±)11-HEPE	(±)11-hydroxy-5Z,8Z,12E,14Z,17Z eicosapentaenoic acid	Cayman Chemical	32500	8,5	317.2 > 166.7	d8-12-HETE
11-HETE	(±)11-HETE	11-hydroxy-5Z,8Z,11E,14Z-eicosatetraenoic acid	Cayman Chemical	34500	9,8	319.2 > 166.8	d8-12-HETE
12(13)-EpODE	12,13-EpODE	12,13-epoxy-9Z,15Z-octadecadienoic acid	Larodan	14-1803-16a-1	9,6	293.2 > 183.0	d4-9(10)-EpOME
12,13-DiHOME (erythro)	(±)12,13-DiHOME	(±)12,13-dihydroxy-9Z-octadecenoic acid	Cayman Chemical	10009832	6,0	313.1 > 183.1	d4-12,13-DiHOME
12,13-DiHOME (threo)	(±)12,13-DiHOME	(±)12,13-dihydroxy-9Z-octadecenoic acid	Cayman Chemical	10009832	6,5	313.1 > 183.1	d4-12,13-DiHOME
12-HEPE	(±)12-HEPE	(±)12-hydroxy-5Z,8Z,10E,14Z,17Z-eicosapentaenoic acid	Cayman Chemical	32540	8,8	317.0 > 178.8	d8-12-HETE
12-HETE	(±)12-HETE	(±)12-hydroxy-5Z,8Z,10E,14Z-eicosatetraenoic acid	Cayman Chemical	34550	10,0	319.1 > 179.0	d8-12-HETE
12-HHTrE	12(S)-HHTa	12S-hydroxy-5Z,8E,10E-heptadecatrienoic acid	Cayman Chemical	34590	7,3	279.2 > 179.0	d4-13-HODE
12-KETE	12-oxo-ETE	12-oxo-5Z,8Z,10E,14Z-eicosatetraenoic acid	Cayman Chemical	34580	10,1	317.2 > 153	d6-5-KETE
13-HODE	(±)13-HODE	(±)-13-hydroxy-9Z,11E-octadecadienoic acid	Cayman Chemical	38600	9,1	295.2 > 195.0	d4-13-HODE
13-HOTrE	13(S)-HOTrE	13S-hydroxy-9Z,11E,15Z-octadecatrienoic acid	Cayman Chemical	39620	8,1	293.2 > 195.2	d4-13-HODE
13-HOTrEγ	13(S)-HOTrEγa	13S-hydroxy-6Z,9Z,11E-octadecatrienoic acid	Cayman Chemical	39610	8,3	293.2 > 193.0	d4-13-HODE
13-KODE	13-oxo-ODE	13-oxo-9Z,11E-octadecadienoic acid	Cayman Chemical	38620	9,5	293.2 > 112.9	d3-9-KODE
14(15)-EpETrE	(±)14,15-EpETrE	(±)14,15-epoxy-5Z,8Z,11Z-eicosatrienoic acid	Cayman Chemical	50651	10,8	319.3 > 219.1	d11-11(12)-EpETrE
14,15-DiHETE	(±)14,15-DiHETE	(±)14,15-dihydroxy-5Z,8Z,11Z,17Z-eicosatetraenoic acid	Cayman Chemical	10006998	6,3	335.2 > 207.1	d11-11,12-DiHETrE
14,15-DiHETrE	(±)14,15-DiHETrE	(±)14,15-dihydroxy-5Z,8Z,11Z-eicosatrienoic acid	Cayman Chemical	51651	7,2	337.3 > 207.0	d11-14,15-DiHETrE
14-HDoHE	(±)14-HDoHE	(±)14-hydroxy-4Z,7Z,10Z,12E,16Z,19Z-docosahexaenoic acid	Cayman Chemical	33550	9,8	343.2 > 281.2	d8-15-HETE
15-HETE	(±)15-HETE	(±)15-hydroxy-5Z,8Z,11Z,13E-eicosatetraenoic acid	Cayman Chemical	34700	9,3	319.1 > 174.8	d8-15-HETE
15-KETE	15-oxo-ETE	15-oxo-5Z,8Z,11Z,13E-eicosatetraenoic acid	Cayman Chemical	34730	9,7	317.2 > 112.9	d6-5-KETE
17,18-DiHETE	(±)17,18-DiHETE	(±)17,18-dihydroxy-5Z,8Z,11Z,14Z-eicosatetraenoic acid	Cayman Chemical	10006999	5,9	335.2 > 247.1	d11-14,15-DiHETrE
19(20)-EpDPE	(±)19,20-EpDPE	(±)19,20-epoxy-4Z,7Z,10Z,13Z,16Z-docosapentaenoic acid	Cayman Chemical	10175	10,5	343.3 > 281.2	d11-11(12)-EpETrE
19,20-DiHDPA	(±)19,20-DiHDPA	(±)19,20-dihydroxy-4Z,7Z,10Z,13Z,16Z-docosapentaenoic acid	Cayman Chemical	10007001	7,2	361.2 > 229.1	d11-14,15-DiHETrE
20-HETE	20-HETE	20-hydroxy-5Z,8Z,11Z,14Z-eicosatetraenoic acid	Cayman Chemical	90030	8,4	319.2 > 275.1	d6-20-HETE
4-HDoHE	(±)4-HDoHE	(±)4-hydroxy-5E,7Z,10Z,13Z,16Z,19Z-docosahexaenoic acid	Cayman Chemical	33200	10,7	343.2 > 101.0	d8-5-HETE
5,6-DiHETrE	(±)5,6-DiHETrE	(±)5,6-dihydroxy-8Z,11Z,14Z-eicosatrienoic acid	Cayman Chemical	51211	8,7	337.3 > 144.9	d11-8,9-DiHETrE
5-HEPE	(±)5-HEPE	(±)5-hydroxy-6E,8Z,11Z,14Z,17Z-eicosapentaenoic acid	Cayman Chemical	32200	9,1	317.3 > 114.8	d8-5-HETE
5-HETE	(±)5-HETE	(±)5-hydroxy-6E,8Z,11Z,14Z-eicosatetraenoic acid	Cayman Chemical	34210	10,5	319.1 > 114.9	d8-5-HETE
5-iPF2α-VI	(±)5-iPF2α-VI	5,9α,11α-trihydroxy-(8β)-prosta-6E,14Z-dien-1-oic acid	Cayman Chemical	16300	2,3	353.2 > 114.9	d11-5-iPF2a-VI
5-KETE	5-oxo-ETE	5-oxo-6E,8Z,11Z,14Z-eicosatetraenoic acid	Cayman Chemical	34250	11,0	317.2 > 203.1	d6-5-KETE
8(9)-EpETrE	(±)8,9-EpETrE	(±)8,9-epoxy-5Z,11Z,14Z-eicosatrienoic acid	Cayman Chemical	50351	11,3	319.3 > 155.0	d11-8(9)-EpETrE
8,9-DiHETrE	(±)8,9-DiHETrE	(±)8,9-dihydroxy-5Z,11Z,14Z-eicosatrienoic acid	Cayman Chemical	51351	8,1	337.2 > 126.9	d11-8,9-DiHETrE
8-HDoHE	(±)8-HDoHE	(±)8-hydroxy-4Z,6E,10Z,13Z,16Z,19Z-docosahexaenoic acid	Cayman Chemical	33350	10,2	343.2 > 189.0	d8-5-HETE
8-HETrE	8(S)-HETrEa	8S-hydroxy-9E,11Z,14Z-eicosatrienoic acid	Cayman Chemical	36360	10,5	321.3 > 157.0	d8-5-HETE
9,10,13-TriHOME	9(S),10(S),13(S)-TriHOMEa	9S,10S,13S-trihydroxy-11E-octadecenoic acid	Larodan	14-1802-15	2,5	329.0 > 139.0	d4-PGE2
9,10-DiHOME (erythro)	(±)9,10-DiHOME	(±)9,10-dihydroxy-12Z-octadecenoic acid	Cayman Chemical	53400	6,5	313.2 > 201.1	d4-9,10-DiHOME
9,10-DiHOME (threo)	(±)9,10-DiHOME	(±)9,10-dihydroxy-12Z-octadecenoic acid	Cayman Chemical	53400	6,9	313.2 > 201.1	d4-9,10-DiHOME
9,12,13-TriHOME	9(S),12(S),13(S)-TriHOME	9S,12S,13S-trihydroxy-10E-octadecenoic acid	Larodan	14-1802-14	2,4	329.1 > 211.0	d4-PGE2
9-HETE	(±)9-HETE	(±)9-hydroxy-5Z,7E,11Z,14Z-eicosatetraenoic acid	Cayman Chemical	34400	10,2	319.2 > 179.1	d8-12-HETE
9-HODE	(±)9-HODE	(±)9-hydroxy-10E,12Z-octadecadienoic acid	Cayman Chemical	38400	9,2	295.3 > 171.0	d4-9-HODE
9-HOTrE	9(S)-HOTrEa	9S-hydroxy-10E,12Z,15Z-octadecatrienoic acid	Cayman Chemical	18500	7,9	293.2 > 170.9	d4-9-HODE
9-KODE	9-KODE	9-oxo-10E,12Z-octadecadienoic acid	Cayman Chemical	38420	9,7	293.2 > 185.0	d3-9-KODE
9-KOTrE	9-KOTrE	9-oxo-10E,12Z,15Z-octadecatrienoic acid	Cayman Chemical	10546	8,6	291.2 > 185	d3-9-KODE
12(13)-EpOME (cis)	(±)12,13-EpOME	(±)12,13-epoxy-9Z-octadecenoic acid	Cayman Chemical	52450	10,6	295.2 > 194.9	d4-9(10)-EpOME
9(10)-EpOME (cis)	(±)9,10-EpOME	(±)9,10-epoxy-12Z-octadecenoic acid	Cayman Chemical	52400	10,8	295.2 > 170.9	d4-9(10)-EpOME
LTE4	LTE4	5S-hydroxy-6R-(S-cysteinyl)-7E,9E,11Z,14Z-eicosatetraenoic acid	Cayman Chemical	20410	5,4	438.4 > 333.3	d5-LTE4
LXA4 / epiLXA4	LXA4	5S,6R,15S-trihydroxy-7E,9E,11Z,13E-eicosatetraenoic acid	Cayman Chemical	20410	3,7	351.2 > 115	d5-LXA4
PGD2	PGD2	9α,15S-dihydroxy-11-oxo-prosta-5Z,13E-dien-1-oic acid	Cayman Chemical	12010	3,0	351.2 > 271.1	d4-PGD2
PGE1	PGE1	9-oxo-11α,15S-dihydroxy-prost-13E-en-1-oic acid	Cayman Chemical	13010	2,8	353.3 > 317.3	d4-PGD1
PGE2	PGE2	9-oxo-11α,15S-dihydroxy-prosta-5Z,13E-dien-1-oic acid	Cayman Chemical	14010	2,7	351.2 > 271.1	d4-PGE2
PGF2α	PGF2α	9α,11α,15S-trihydroxy-prosta-5Z,13E-dien-1-oic acid	Cayman Chemical	16010	2,5	353.3 > 193.0	d4-8-epi-PGF2a
12(13)-EpOME (trans)	(±)12,13-EpOME	(±)12,13-epoxy-9Z-octadecenoic acid	Cayman Chemical	52450	10,8	295.2 > 194.9	d4-9(10)-EpOME
9(10)-EpOME (trans)	(±)9,10-EpOME	(±)9,10-epoxy-12Z-octadecenoic acid	Cayman Chemical	52400	11,0	295.2 > 170.9	d4-9(10)-EpOME
TXB2	TXB2	9α,11,15S-trihydroxythromba-5Z,13E-dien-1-oic acid	Cayman Chemical	19030	2,0	369.2 > 168.9	d4-TBX2

Screened but not reported

Compound Name	Standard	Systematic Name	Supplier	Cat Number	Rt (min)	SRM transition	IS
11-HDoHE	(±)11-HDoHE	(±)11-hydroxy-4Z,7Z,9E,13Z,16Z,19Z-docosahexaenoic acid	Cayman Chemical	33450	10,0	343.3 > 121.0	d8-12-HETE
11-HEDE	(±)11-HEDE	(±)11-hydroxy-12E,14Z-eicosadienoic acid	Cayman Chemical	37500	11,1	323.2 > 199.0	d4-9-HODE
15-HEDE	(±)15-HEDE	(±)15-hydroxy-11Z,13E-eicosadienoic acid	Cayman Chemical	37700	11,1	323.2 > 223.1	d4-13-HODE
15-HEPE	(±)15-HEPE	(±)15-hydroxy-5Z,8Z,11Z,13E,17Z-eicosapentaenoic acid	Cayman Chemical	32700	8,5	317.1 > 219.1	d8-15-HETE

15-HETrE	15(S)-HETrEa	15S-hydroxy-8Z,11Z,13E-eicosatrienoic acid	Cayman Chemical	36720	10,1	321.3 > 221.1	d8-15-HETE
16(17)-EpDPE	(±)16,17-EpDPE	(±)16,17-epoxy-4Z,7Z,10Z,13Z,19Z-docosapentaenoic acid	Cayman Chemical	10174	10,9	343.3 > 233.2	d11-11(12)-EpETrE
17-HDoHE	(±)17-HDoHE	(±)17-hydroxy-4Z,7Z,10Z,13Z,15E,19Z-docosahexaenoic acid	Cayman Chemical	33650	9,5	343.2 > 281.2	d8-15-HETE
18-HEPE	(±)18-HEPE	(±)18-hydroxy-5Z,8Z,11Z,14Z,16E-eicosapentaenoic acid	Cayman Chemical	32840	8,1	317.2 > 215.1	d8-15-HETE
19-HETE	19(S)-HETE	(5Z,8Z,11Z,14Z)-19-hydroxy-5,8,11,14-icosatetraenoic acid	Cayman Chemical	10007766	8,2	319.2 > 275.1	d6-20-HETE
5-HETrE	5(S)-HETrEa	5S-hydroxy-6E,8Z,11Z-eicosatrienoic acid	Cayman Chemical	36230	11,9	321.3 > 205.1	d8-5-HETE
7,17-hydroxy-DPA	7(S),17(S)-hydroxy DPAa	7,17-dihydroxy-8E,10Z,13Z,15E,19Z-docosapentaenoic acid	Cayman Chemical	37730	6,4	361.2 > 143.0	d11-14,15-DiHETrE
8-HEPE	(±)8-HEPE	(±)8-hydroxy-5Z,9E,11Z,14Z,17Z-eicosapentaenoic acid	Cayman Chemical	32340	8,7	317.0 > 154.9	d8-12-HETE
8-HETE	(±)8-HETE	(±)8-hydroxy-5Z,9E,11Z,14Z-eicosatetraenoic acid	Cayman Chemical	34340	10,0	319.0 > 154.9	d8-12-HETE
8-iso-PGE2	8-iso-PGE2	9-oxo-11α,15S-dihydroxy-(8β)-prosta-5Z,13E-dien-1-oic acid	Cayman Chemical	14350	2,6	351.2 > 271.1	d4-isoPGE2
LXA5	LXA5	5S,6R,15S-trihydroxy-7E,9E,11Z,13E,17Z-eicosapentaenoic acid	Cayman Chemical	90410	2,7	349.2 > 114.9	d5-LXA4
PGD1	PGD1	9α,15S-dihydroxy-11-oxo-prost-13E-en-1-oic acid	Cayman Chemical	12000	3,0	353.3 > 317.3	d4-PGD2
PGD3	PGD3	9α,15S-dihydroxy-11-oxo-prosta-5Z,13E,17Z-trien-1-oic acid	Cayman Chemical	20110	2,2	349.3 > 269.3	d4-PGD1
PGE3	PGE3	9-oxo-11α,15S-dihydroxy-prosta-5Z,13E,17Z-trien-1-oic acid	Cayman Chemical	14990	2,0	349.3 > 269.3	d4-PGE2
TXB1	TXB1	9,11,15-trihydroxy-thrombox-13-en-1-oic acid	Cayman Chemical	1006610	1,9	371.2 >171.0	d4-TBX2
TXB3	TXB3	9α,11,15S-trihydroxy-thromba-5Z,13E,17Z-trien-1-oic acid	Cayman Chemical	19990	1,6	367.2 > 195.15	d4-TBX2
